# Anti‐Inflammatory Mechanisms of Selenium Nanosheets in Ulcerative Colitis: Protein Corona, GP130 Interaction, and Transcriptomic Profile

**DOI:** 10.1002/advs.202501832

**Published:** 2025-06-29

**Authors:** Dingyi Shen, Li Gong, Wei Yang, Jiaqi Luo, Zhen Jin, Youzhi Tang

**Affiliations:** ^1^ Guangdong Laboratory for Lingnan Modern Agriculture, College of Veterinary Medicine South China Agriculture University Guangzhou Guangdong 510642 China; ^2^ Instrumental Analysis Research Center Sun Yat‐sen University Guangzhou Guangdong 510275 China

**Keywords:** anti‐inflammatory, GP130, protein corona, selenium nanosheet, ulcerative colitis

## Abstract

Ulcerative colitis (UC) is a complex inflammatory bowel disease characterized by multiple factors. Alleviating inflammation is the primary therapeutic approach. However, currently employed anti‐inflammatory treatments have limited efficacy and cause side effects. Safer, more effective therapies are needed. Selenium nanosheets (SeNSs) are biocompatible, anti‐inflammatory, and low‐toxicity nanomaterials with high surface areas and abundant active sites, making them potential therapeutic agents for UC. This study indicates that SeNSs can interact with macrophages and adhere to their cell membranes, significantly increasing their internalization into cells. Proteomic analysis reveals that the main components of the SeNS protein corona are proteins involved in cell proliferation and migration, including those associated with the AKT/PI3K and NF‐κB signaling pathways. SeNSs hydrophobically interact with GP130, inhibiting its expression. This interaction downregulates the proteins involved in the aforementioned pathways. In addition, a transcriptomic analysis confirms that SeNSs inhibit apoptosis, cytokine–cytokine receptor interactions, and the chemokine and TNF signaling pathways. In dextran sulfate sodium (DSS)‐induced UC model mice, SeNSs significantly decrease IL‐1β, IL‐6, and TNF‐α levels, alleviate tissue damage, and lower the disease activity index. These findings suggest that SeNSs can be a safe and effective treatment strategy for UC, offering a novel approach for managing inflammatory diseases.

## Graphical abstract

SeNSs provide a biocompatible, anti‐inflammatory UC therapy. SeNSs form protein coronas enriched with AKT/PI3K/NF‐κB pathway proteins, suppress GP130 via hydrophobic interactions, and inhibit pro‐inflammatory cytokines. In DSS‐induced UC mice, SeNSs reduce inflammation, tissue damage, and disease activity by modulating cytokine, chemokine, and apoptosis pathways, making them a safe and effective UC treatment strategy.

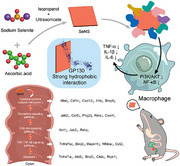

## 1 Introduction

Ulcerative colitis (UC) is a complex inflammatory bowel disease influenced by genetics, environmental influences, immunological regulation, and infections.^[^ ^1^ ^]^ In the last 25 years, UC has become increasingly common in North America and Europe,^[^ ^2^, ^3^, ^4^, ^5^ ^]^ and its incidence has increased rapidly in developing countries, particularly in Asia.^[^ ^6^, ^7^, ^8^ ^]^ An excessive inflammatory response in the colon is the predominant characteristic of UC. Inflammation is often associated with pain, intestinal hemorrhage, and dysbiosis, significantly impairing the quality of life of UC patients.^[^ ^9^ ^]^

Currently, small‐molecule anti‐inflammatory drugs, such as mesalazine and corticosteroids, are commonly used to mitigate intestinal inflammation.^[^ ^10^ ^]^ However, long‐term use of these anti‐inflammatory agents may lead to side effects, including allergic reactions and exacerbated symptoms, which decrease the efficacy of the treatment.^[^ ^11^, ^12^ ^]^ Immunosuppressive drugs and anti‐TNF‐α monoclonal antibodies have been developed to prevent these side effects. However, their effectiveness varies from person to person.^[^ ^13^ ^]^ In recent years, the potential of nanomaterials (Mo_3_Se_4_ nanoparticles, turmeric‐derived exosome‐like nanovesicles, and chondroitin sulfate‐wrapped poly(β‐aminoester)‐SA‐PAPE copolymer nanoparticles) to treat inflammatory diseases has received increasing attention.^[^ ^14^, ^15^, ^16^ ^]^ Nevertheless, these nanomaterials have not yet entered clinical trials to treat UC. The underlying reasons may include excessive immune responses caused by some nanomaterials, limited therapeutic efficacy, or unclear mechanisms of action.^[^ ^17^ ^]^

Selenium is an essential trace element for both humans and animals.^[^ ^18^ ^]^ Amino acids containing selenium are closely related to oxidoreductases with various biological effects, such as anti‐inflammatory, antioxidant, and anti‐iron toxicity effects.^[^ ^19^, ^20^ ^]^ Selenium nanoparticles (SeNPs), as a potential material for treating UC, have anti‐inflammatory properties, high biocompatibility, low toxicity, and high bioavailability.^[^ ^21^, ^22^ ^]^ Many studies have shown important roles of SeNPs in immune regulation, nervous system disease therapy, cancer treatment, alleviation of diabetes, and mitigation of oxidative stress.^[^ ^22^, ^23^ ^]^ The small size, multiple surface charges, and biocompatible coating of SeNPs influence their interactions with cells and biomacromolecules, modulating cell metabolism and signaling.^[^ ^24^, ^25^ ^]^ Thus, SeNPs are a promising drug candidate for treating UC. Moreover, the functionalization of SeNPs could increase their biological activity.^[^ ^26^, ^27^, ^28^, ^29^ ^]^ Compared with SeNPs, selenium nanosheets (SeNSs), a type of 2D nanomaterial, exhibit a greater optical response, excellent electro‐optical performance, and a larger specific surface area.^[^ ^30^ ^]^ The potential applications of SeNSs are broad and include biosensing, drug loading, and bioimaging research.^[^ ^30^ ^]^ This two‐dimensional material has a larger surface area and more active sites than traditional SeNPs, which are significantly different in structure and performance. However, whether SeNSs exhibit greater biological activity than SeNPs is unclear. Moreover, the mechanism through which SeNSs exert their effects in vivo remains to be clarified.

**Figure 1:**
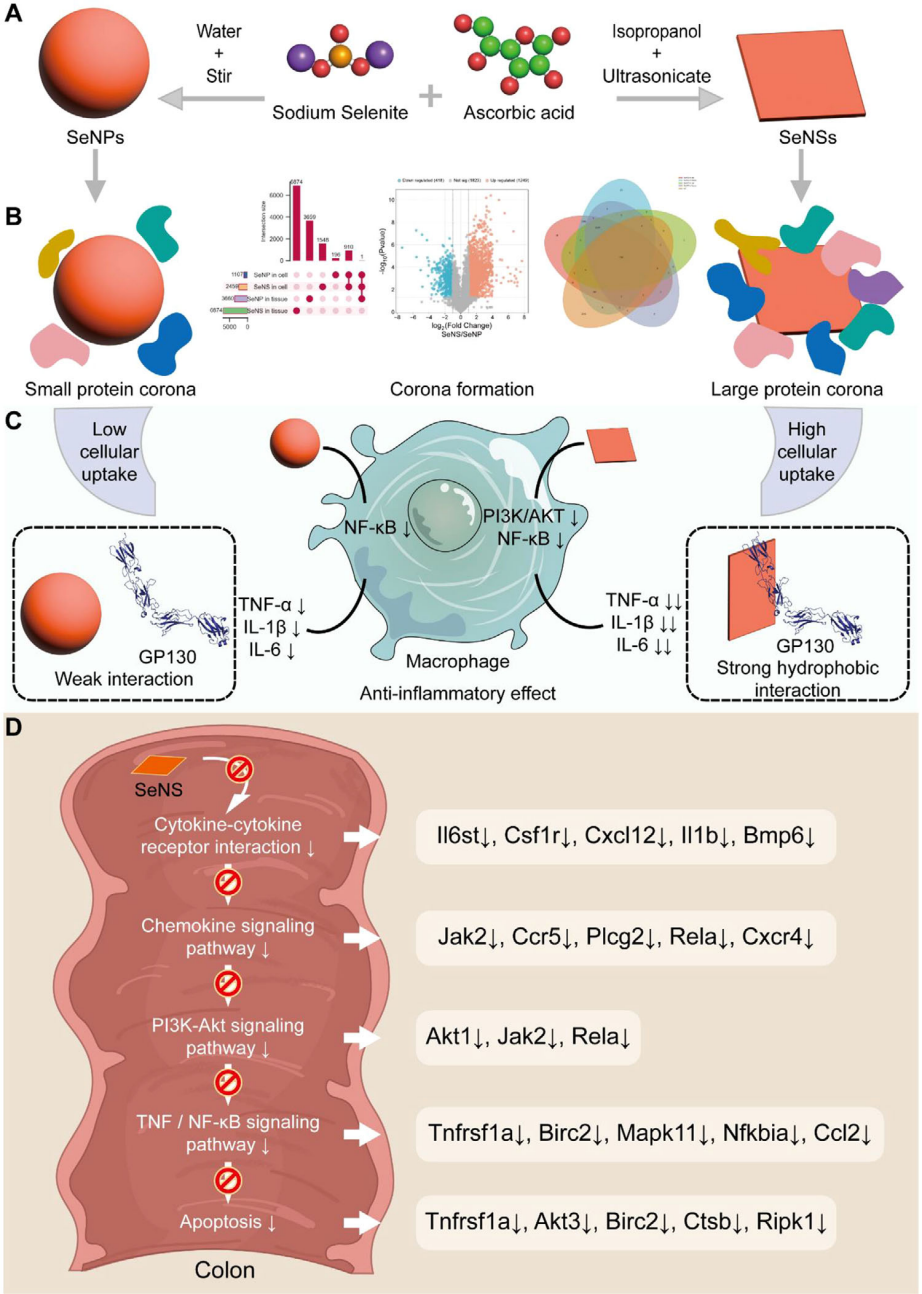
Schematic diagram of the synthesis and anti‐inflammatory mechanisms of SeNSs and SeNPs. A) Process used to synthesize the SeNPs and SeNSs. B) Proteomic analysis of the SeNS and SeNP protein corona. C) Anti‐inflammatory effects of the SeNSs and SeNPs on macrophages. D) Transcriptomic analysis of mouse colon tissues to elucidate the anti‐inflammatory mechanism of SeNSs.

Here, we constructed and analyzed ultrathin SeNSs with anti‐inflammatory properties and in vivo and in vitro activities. These SeNSs had low toxicity and good dispersion and stability (**Figure** **1**A). Compared with ordinary SeNPs, SeNSs exhibited greater anti‐inflammatory activity. They adsorbed large amounts of protein and formed a protein corona, which facilitated their uptake by macrophages (Figure 1B). We characterized the SeNS protein coronas via proteomics and investigated the interaction between SeNSs and GP130 in cells and colon tissue. The SeNSs hydrophobically interacted with the membrane protein GP130, which specifically adsorbed and downregulated proteins in the AKT/PI3K and NF‐κB pathways (Figure 1C). Moreover, SeNSs inhibited cytokine‒cytokine receptor interaction, chemokine signaling pathway, PI3K‐Akt signaling network, TNF/NF‐κB signaling pathway, and apoptosis in the colon tissues of UC model mice, thereby exerting multiple anti‐inflammatory effects (Figure 1D). These multiple effects greatly enhanced the anti‐inflammatory activity of these SeNSs, suggesting their use as a safe and effective treatment for UC. This study provides new insights into the design of nanomedicines and strong evidence that SeNSs may be a promising treatment for UC.

## 2 Results and Discussion

### 2.1 Synthesis and Characterization of the SeNSs and SeNPs

**Figure 2:**
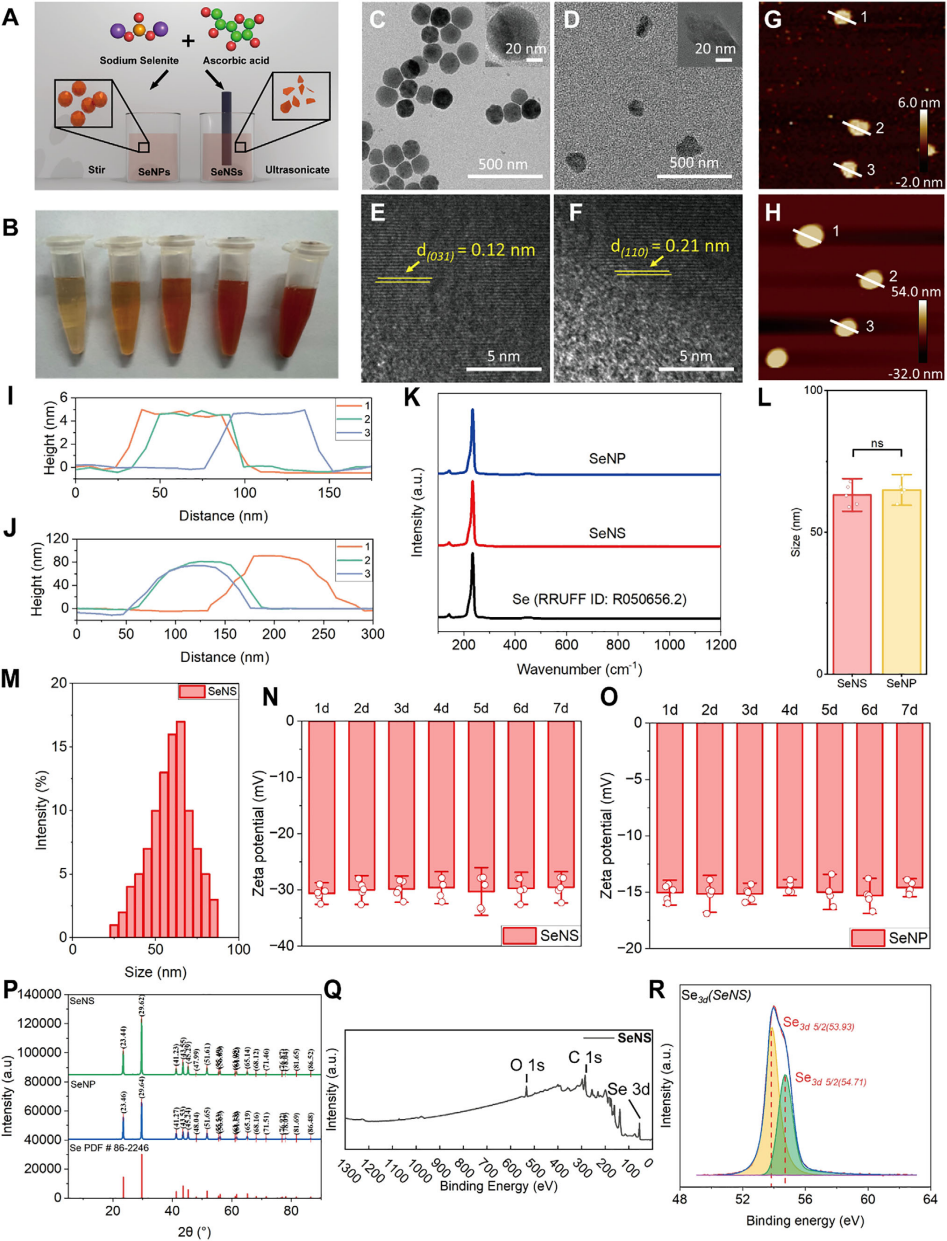
Characterization of SeNSs and SeNPs. A) Schematic diagram of the methods used to synthesize the SeNSs and SeNPs. B) Color of aqueous solutions of SeNSs at different concentrations (left to right: 1, 2, 3, 4, and 5 mM). C,D) TEM images of the SeNPs C) and SeNSs D). E, F) HRTEM images of the SeNPs E) and SeNSs F).G,H) AFM images of the SeNSs G) and SeNPs H).I, J) Thicknesses of the SeNSs I) and SeNPs J). K) Raman spectra.L) Comparison of particle sizes.M) DLS data for the SeNSs.N, O) Changes in the zeta potential of the SeNSs N) and SeNPs O) over 7 days.P)XRD patterns of the SeNSs and SeNPs.Q) XPS spectra of the SeNSs.R) Se*_3d_* spectra of the SeNSs. (*P < 0.05, **P < 0.01, ***P < 0.001, ****P < 0.0001; data are expressed as mean ± standard deviation, *n* = 5 independent experiments, student‐t test, and one‐way ANOVA).

SeNSs and SeNPs were prepared via the oxidation‒reduction of ascorbic acid and sodium selenite (**Figure** **2**A). The method and conditions for nanoselenium synthesis were optimized (Table S1). SeNPs and SeNSs of similar sizes but different thicknesses were efficiently obtained. The SeNS solution was orange‒red in color and changed to dark red as the SeNS concentration increased at room temperature (Figure 1B). Transmission electron microscopy (TEM) images revealed that the SeNSs had an irregular sheet morphology and high dispersibility, whereas the SeNPs appeared as spherical particles (Figure 2C–F). The average thicknesses of the SeNSs and SeNPs were 4.75 and 79.41 nm, respectively, as measured by atomic force microscopy (AFM) (Figure 2G–J). The Raman spectroscopy results shown in Figure 2K further confirmed the structures of the SeNPs and SeNSs. The resonance peaks of the SeNSs were observed at 144.2 and 235.3 cm⁻¹, whereas those of the SeNPs peaked at 144.3 and 236.3 cm⁻¹, respectively. This result is consistent with the transverse optical phonon mode and the stretching mode of the t‐Se chain structure.^[^ ^31^ ^]^

Dynamic light scattering (DLS) measurements revealed no significant difference in the mean diameter between the SeNSs and SeNPs (Figure 2L,M; Figure S1, Supporting Information). The differences in the diameter and thickness of the SeNSs compared with those of the SeNPs demonstrate their anisotropy. Anisotropy is achieved by liquid‐phase exfoliation, which is attributed to the strong Se‒Se covalent bond in the horizontal direction and the relatively weak van der Waals interactions in the vertical direction.^[^ ^32^ ^]^ In addition, the zeta potential results confirmed the stability of the SeNPs and SeNSs in solution. The zeta potentials of the SeNSs and SeNPs were stable at −30 and −15 mV, respectively, for 7 consecutive days (Figure 2N,O). Notably, on the 10th and 20th days, the particle size of SeNPs increased by 8.72% and 35.17%, respectively, whereas that of SeNSs remained stable on the 20th day, indicating the greater stability of SeNSs (Figure S2, Supporting Information). SeNSs and SeNPs aggregated in gastric juice and adsorbed proteins, forming protein coronas (Figure S3, Supporting Information). As the pH increases, the particle size of SeNS and SeNP decreases, the surface potential increases, and the stability improves (Figure S3, Supporting Information). As the pH increased, the particle size of SeNSs and SeNPs decreased, the surface potential increased, and the stability improved (Figure S4, Supporting Information).

The lattice fringes of the SeNSs and SeNPs matched the lattice fringe values of the trigonal Se phase (t‐Se), as observed using high‐resolution transmission electron microscopy (HRTEM). The lattice fringe value of the SeNPs was ≈0.12 nm, corresponding to the (031) plane, whereas that of the SeNSs was ≈ 0.21 nm, corresponding to the (110) plane. These findings were consistent with the X‐ray diffraction (XRD) results (Figure 2P, Figure S5, Supporting Information). The diffraction peaks of the SeNSs and the SeNPs clearly indicated that they were both in the t‐Se phase.^[^ ^31^ ^]^ The high surface area of the SeNSs resulted in a greater zeta potential and more protein adsorption sites. The X‐ray photoelectron spectroscopy (XPS) results indicated that the SeNSs presented peaks at 54.71 and 53.93 eV (Figure 2Q,R), which were attributed to Se─Se bonds (zero‐valent crystalline Se). The absence of signals at 59 eV suggested that the SeNSs were not oxidized during synthesis, which was consistent with the results for the SeNPs (Figure S6, Supporting Information).

### 2.2 Cellular Uptake and Cytotoxicity of the SeNSs and SeNPs

**Figure 3:**
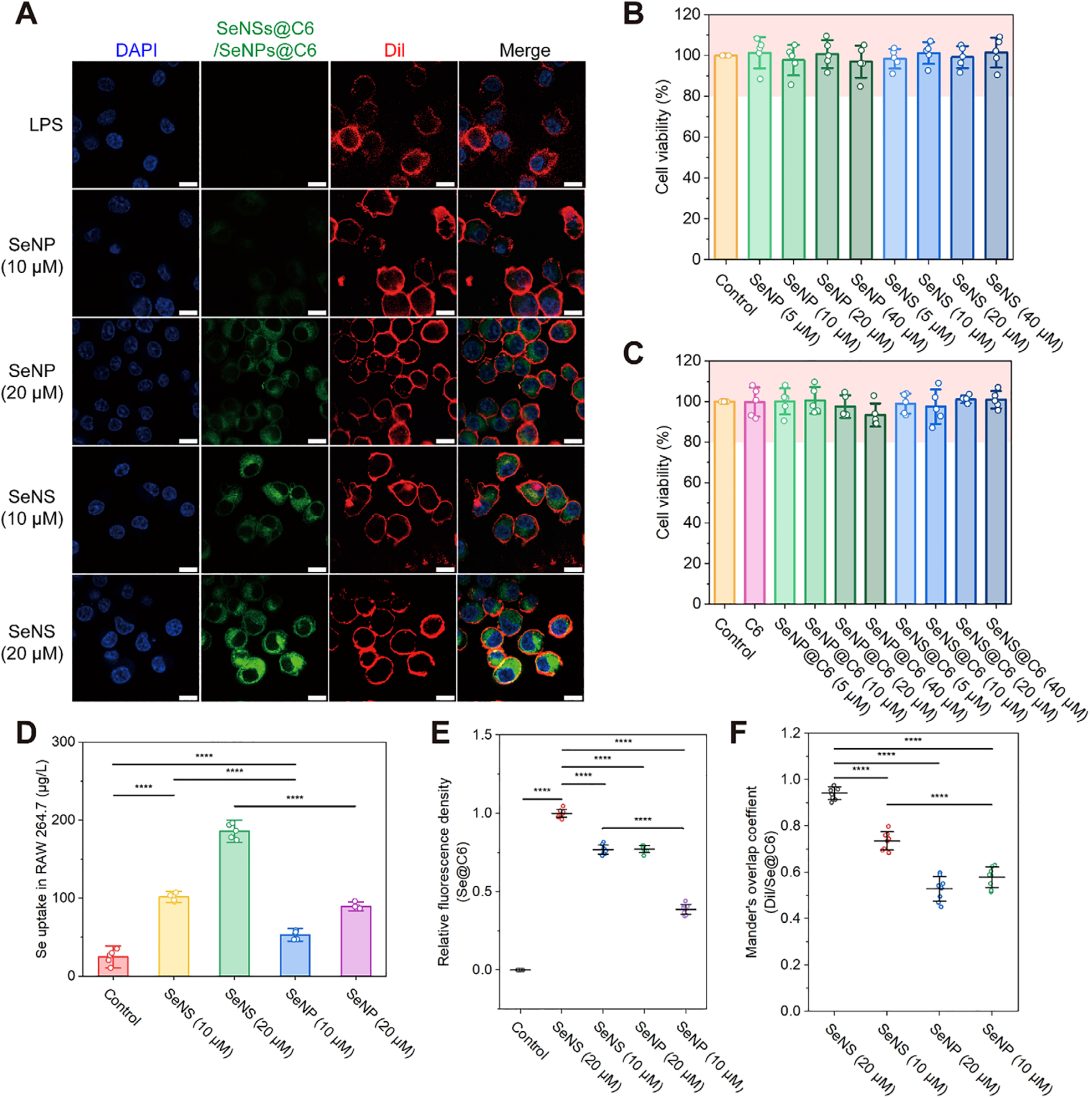
Cellular uptake and cytotoxicity of SeNSs and SeNPs. A) CLSM images depicting the internalization of SeNSs and SeNPs in RAW264.7 cells. Scale bar = 10 µm. B) Cytotoxicity assay of SeNSs and SeNPs in RAW264.7 cells. C) Evaluation of the toxicity of SeNS@C6 and SeNP@C6 to RAW264.7 cells. D) Internalization of SeNSs and SeNPs by RAW264.7 cells was determined via ICP‒MS analysis. E) Fluorescence intensities of SeNSs and SeNPs in RAW264.7 cells. F) Colocalization of SeNSs and SeNPs with the cell membrane. (*p < 0.05, **p < 0.01, ***p < 0.001, ****p < 0.0001; data are expressed as mean ± standard deviation, *n* = 5 independent experiments, student‐t test, and one‐way ANOVA).

To investigate the biological activity of the nanomaterials, we evaluated the toxicity of the SeNSs and SeNPs to RAW264.7 cells as well as their uptake by these cells in vivo. The SeNPs and SeNSs were labeled with coumarin 6 (C6) to observe their cellular uptake. Confocal imaging analysis revealed a concentration‐dependent increase in the intensity of cellular green fluorescence for both SeNP@C6 and SeNS@C6, which was indicative of progressive nanoparticle uptake (**Figure** **3**A). Notably, the fluorescence intensity of SeNS@C6‐treated cells was significantly greater than that of their SeNP@C6 counterparts at equivalent concentrations, suggesting the superior cellular internalization efficiency of the nanosheet formulation.

The MTT assay results demonstrated that after both the SeNS and SeNP treatment, cell viability was maintained above 80% across the tested concentration range (5–40 µm) (Figure 3B), indicating the safety of these materials for cells. Similar observations were also made in Caco‐2 and HT29 cells (Figure S7, Supporting Information). The cell viability treated with the C6‐labeled SeNPs (SeNP@C6) and SeNSs (SeNS@C6) was similar to that of the cells treated with the unlabeled SeNSs and SeNPs, suggesting that C6 labeling had no effect on cell viability (Figure 3C). The ICP‒MS results further confirmed that RAW264.7 cells internalized more SeNSs than SeNPs. When exposed to 20 µm Se, the uptake of SeNS was 1.85 times greater than that of SeNPs (Figure 3D). This trend is consistent with the fluorescence quantification results (Figure 3E). Fluorescence colocalization analysis revealed that SeNSs bound more strongly to the cell membrane than did SeNPs (Figure 3F). Consistent with the TEM results, SeNSs not only accumulated in the cytoplasm and lysosomes after being internalized by RAW164.7 but also adsorbed to the surface of the cell membrane (Figure S8, Supporting Information).

Similar to two‐dimensional nanomaterials such as graphene and bismuthene nanosheets, SeNSs have a high aspect ratio, with an average lateral size of 60.03 nm and a thickness of 4.75 nm. The excellent planar flexibility of these materials may promote the targeting of specific membrane receptor proteins, increasing their endocytosis and cellular uptake. In contrast, spherical nanoparticles have an uneven surface charge density and isotropic dimensions, which hinder macrophage recognition and result in lower uptake efficiency.^[^ ^33^, ^34^ ^]^ In addition, we also measured the distributions of SeNSs and SeNPs in vivo and found that SeNSs and SeNPs accumulated mainly in the liver and colon of mice, and their levels decreased slowly over time. However, the content of SeNSs in these tissues was greater than that of SeNPs (Figure S9, Supporting Information).

### 2.3 Evaluation of the Anti‐Inflammatory Activity of SeNSs and SeNPs

**Figure 4:**
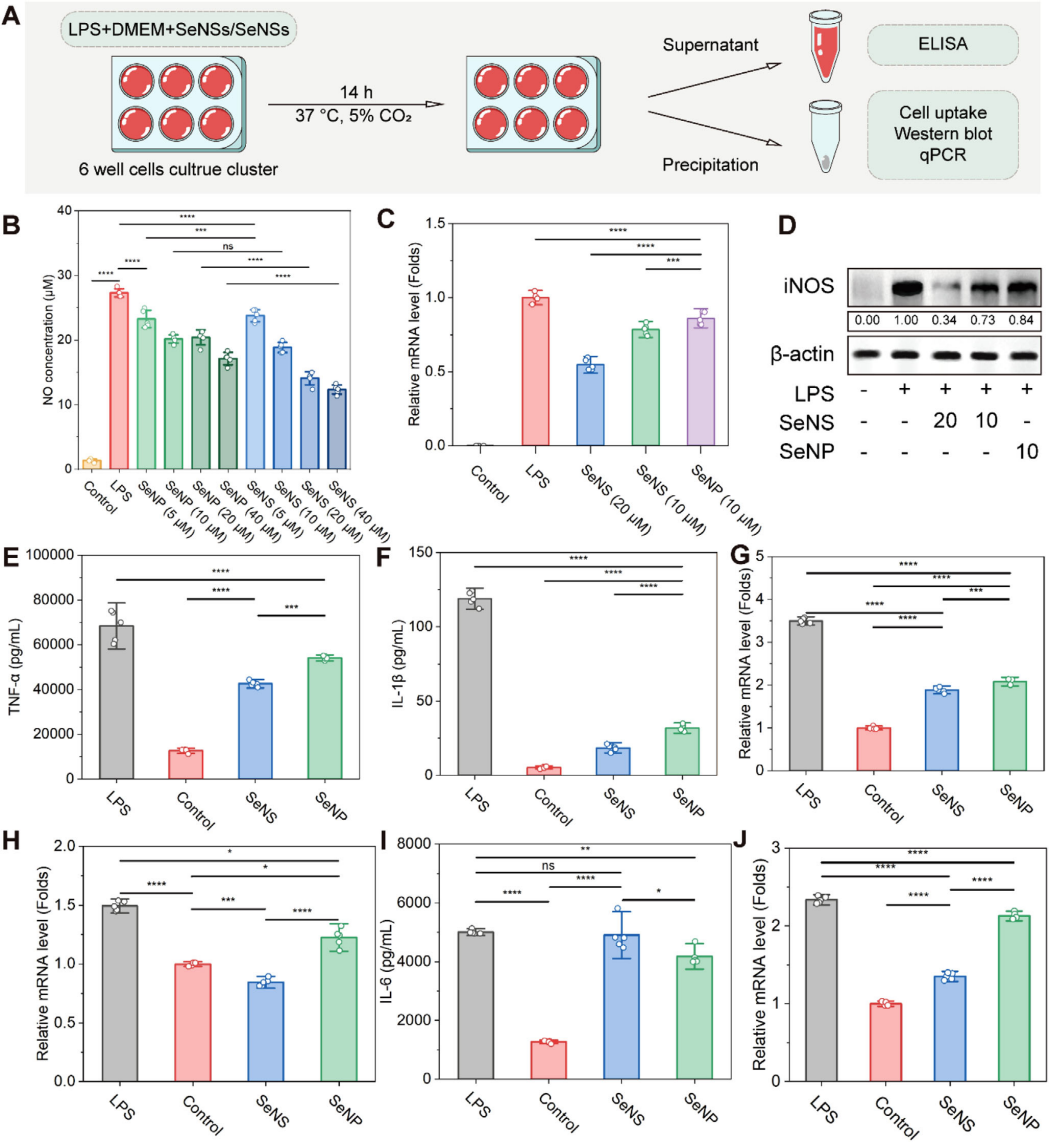
Evaluation of the anti‐inflammatory activity of the SeNSs and SeNPs in vitro. A) Schematic diagram. B) NO concentration under different treatments (control, 1 µm LPS, 5, 10, 20, and 40 µm SeNPs or SeNSs). C) mRNA expression levels of iNOS. D: Western blot analysis of iNOS levels. E,F) Concentrations of TNF‐α Ε) and IL‐1β F). G,H) mRNA expression levels of TNF‐α G) and IL‐1β H). I,J) Concentration I) and mRNA expression level of IL‐6 J). (*p < 0.05, **p < 0.01, ***p < 0.001, ****p < 0.0001; data are expressed as mean ± standard deviation, *n* = 5 independent experiments, student‐t test, and one‐way ANOVA).

Macrophages can promote or inhibit inflammatory responses, which play crucial roles in the development of inflammatory diseases.^[^ ^35^, ^36^ ^]^ RAW264.7 cells were used to evaluate the anti‐inflammatory effects of the SeNSs and SeNPs in vitro. Lipopolysaccharide (LPS) was added to the cell culture medium to induce M1 polarization of the macrophages.^[^ ^37^ ^]^ Different concentrations of SeNSs and SeNPs were coincubated with the cells for 14 h. **Figure** **4**A shows a schematic diagram of the in vitro experimental protocol. After LPS induction, the cells polarized into M1 macrophages and produced high levels of proinflammatory cytokines (TNF‐α, IL‐6, and IL‐1β) and NO.^[^ ^37^ ^]^ SeNSs inhibited NO production in a concentration‐dependent manner within the 5–40 µm range, reducing NO levels by 12.89–54.77%, which was significantly greater than the NO clearance rate of SeNPs (14.77–37.21%) at the same concentrations (Figure 4B). Notably, the NO scavenging ability of the SeNSs and SeNPs was unaffected by C6 labeling (Figure S10, Supporting Information).

Excessive NO production can lead to tissue damage and a proinflammatory response, serving as an inflammatory biomarker.^[^ ^38^ ^]^ The inhibitory effect of SeNSs on NO was related to their ability to inhibit the expression of inducible nitric oxide synthase (iNOS) and cyclooxygenase‐2 (COX‐2).^[^ ^38^ ^]^ In subsequent experiments, the cells were treated with 10 µm SeNPs or SeNSs, after which iNOS mRNA and protein expression were evaluated. SeNSs inhibited the expression of the iNOS mRNA and reduced its protein level. This inhibitory effect increased with increasing SeNS concentration (Figure 4C,D, Figure S11 (Supporting Information). Additionally, SeNSs demonstrated significantly greater GPX enzymatic activity and superior ROS scavenging efficiency than did SeNPs (Figures S12, S13, Supporting Information).

Compared with the SeNP treatment group, the SeNS treatment group presented significantly lower expression of TNF‐α and IL‐1β when the cells were exposed to the same concentrations of the nanomaterials. This trend was consistent with the mRNA expression levels, confirming the increased anti‐inflammatory activity of SeNS (Figure 4E–H). Notably, although the mRNA expression level of IL‐6 decreased, the protein level of IL‐6 increased after SeNS treatment (Figure 4I,J). This observation might be related to the regulation of IL‐6‐related receptor activity by SeNSs, leading to the ineffective degradation of IL‐6. Similar to immunoglobulin (IVIg), anti‐IL‐6 autoantibodies (aAb IL‐6) interfere with the binding of the IL‐6 receptor (GP130) to the IL‐6/IL‐6R complex, preventing the cellular uptake and degradation of IL‐6, which increases IL‐6 protein levels.^[^ ^39^ ^]^

**Figure 5:**
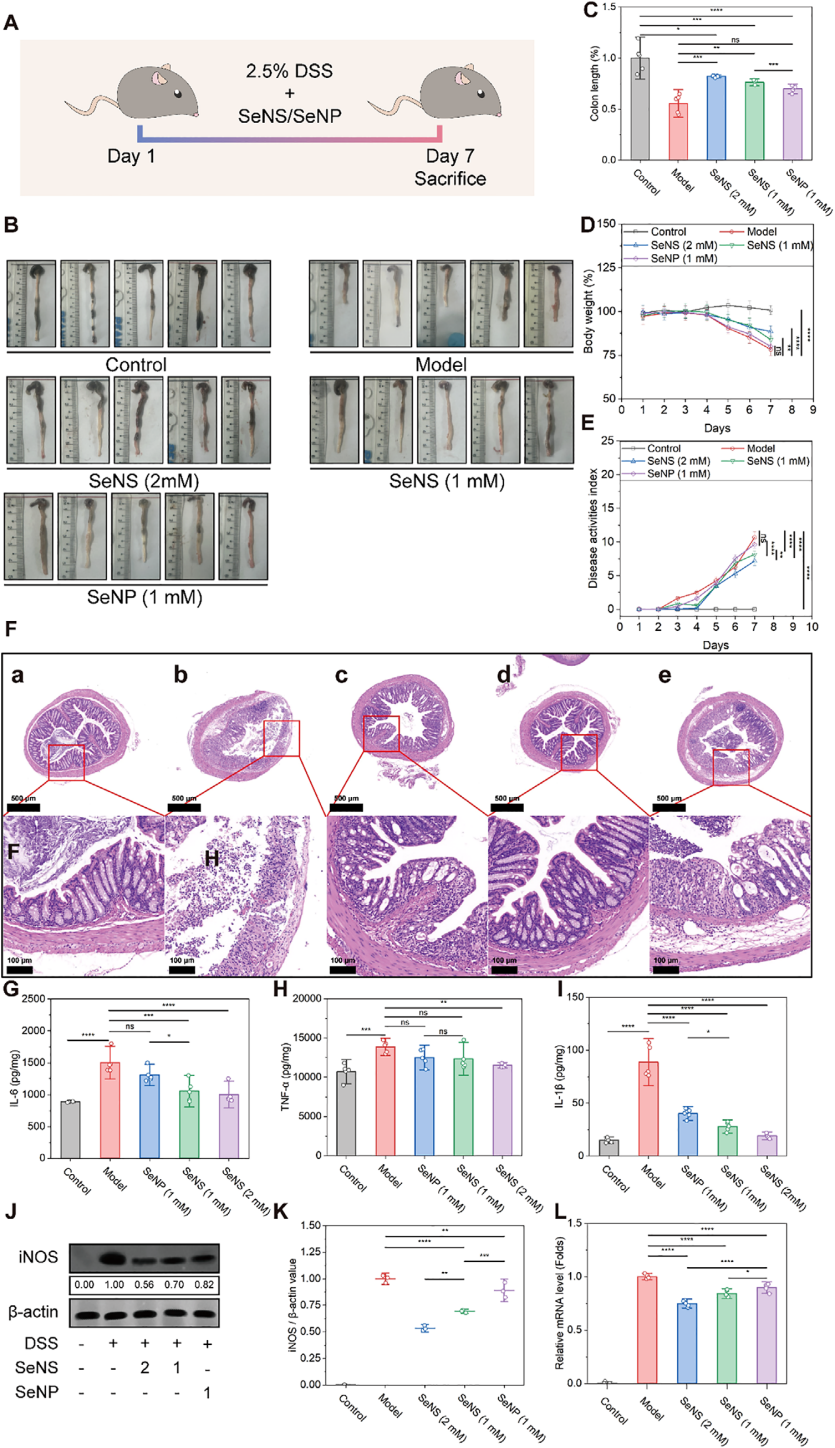
Evaluation of the anti‐inflammatory activity of SeNSs and SeNPs in vivo. A) Schematic diagram of SeNS/SeNP therapy in a UC mouse model. B) Mouse colon length was measured on day 7 after sacrifice. C) Quantification of colon length; D) body weight of the mice during the 7‐day treatment. E) Disease activity index (DAI) of the mice. The DAI is the sum of the stool consistency index (0–3), the fecal bleeding index (0–3), and the weight loss index (0–4). F) H&E‐stained colonic tissue sections from mice in the a) control group, b) model group, c) 1 mm SeNS treatment group, d) 2 mm SeNS treatment group, and e) 1 mm SeNP treatment group. G,I) Concentrations of IL‐6 G), TNF‐α Η), and IL‐1β Ι) in the mice subjected to different treatments in vivo, respectively. J) Western blot results for iNOS. K) Quantification of the Western blot results for iNOS. L) mRNA expression level of iNOS in colon tissues. (*p < 0.05, **p < 0.01, ***p < 0.001, ****p < 0.0001; data are expressed as mean ± standard deviation, *n* = 5 independent experiments, student‐t test, and One‐way ANOVA).

Next, SeNSs and SeNPs were used to treat DSS‐induced acute UC model mice to evaluate their in vivo anti‐inflammatory effects. The SeNSs and SeNPs were administered to the mice by gavage for 7 consecutive days. **Figure** **5**A illustrates the treatment regimen for the acute UC model mice. Since acute UC causes weight loss in mice, the potential impact of the SeNS and SeNP treatments on weight loss should be excluded.^[^ ^40^ ^]^ The SeNS and SeNP monotherapy groups were used as controls. As shown in Figure S14 (Supporting Information), no significant decrease in body weight was observed in the mice after the oral administration of SeNSs or SeNPs. Moreover, H&E staining and blood biochemical indicators revealed that SeNSs and SeNPs did not cause any damage to the heart, liver, spleen, lungs, or kidneys after 7 days of continuous oral administration, nor did they impair liver or kidney function (Figures S15, S16, Supporting Information).

Compared with those in the healthy group, the mice in the model group had a shortened colon length of 3.5–4.5 cm (Figure 5B,C) and a body weight loss of 16.29%–28.62% (Figure 5D). The SeNSs effectively alleviated weight loss, mitigated colon shortening, and decreased the disease activity index in UC model mice (Figure 5C–E). Increasing the concentration of SeNSs further reduced the disease severity of UC. The hematoxylin and eosin (H&E) staining results revealed colonic tissue damage in the model group. The colonic mucosa exhibited congestion, edema, inflammatory cell infiltration in the submucosa, a loss of goblet cells and crypts, and severe tissue damage (Figure 5F). The SeNS and SeNP groups presented reduced congestion, edema, and inflammatory cell infiltration. The intestinal crypts remained intact in the SeNS group, indicating a better therapeutic effect on UC.

In addition, the concentrations and mRNA levels of TNF‐α, IL‐6, and IL‐1β were increased in the model group (Figure 5G–I; Figure S17, Supporting Information). Compared with SeNP treatment, SeNS treatment inhibited the expression of inflammatory cytokines in the UC model mice. SeNS treatment also reduced iNOS expression, which was consistent with the decrease in iNOS mRNA levels (Figure 5J–L). These findings highlight the significant anti‐inflammatory effects of SeNSs in the UC model mice.

### 2.4 Characterization of the Protein Coronas

Nanoparticles adsorb many proteins in the biological environment and form a protein corona.^[^ ^41^ ^]^ SeNSs and SeNPs form protein coronas with varying concentrations in gastric juice, bile, pancreatic fluid, and small intestine fluid, increasing in that order (Figure S18, Supporting Information). Notably, metabolism‐related proteins adsorbed by SeNSs and SeNPs are more abundant in gastric juice and pancreatic juice than in bile and small intestine fluid. Both the formation and quantity of the protein corona influence the bioactivity of nanoparticles.^[^ ^41^ ^]^ The protein coronas formed by the SeNSs in RAW264.7 cells and colon tissues were analyzed to further explore the anti‐inflammatory effects of the SeNSs. The SeNSs and SeNPs were incubated with RAW264.7 cells for 2 h, followed by cell lysis and centrifugation to isolate the protein coronas attached to the surfaces of the SeNSs and SeNPs. This process simulates the protein corona formation process by SeNSs and SeNPs in macrophages. Similarly, SeNSs and SeNPs were incubated with digested colon tissue for 2 h to simulate protein corona formation by SeNSs and SeNPs in the colon. The protein coronas were isolated via centrifugation.

**Figure 6:**
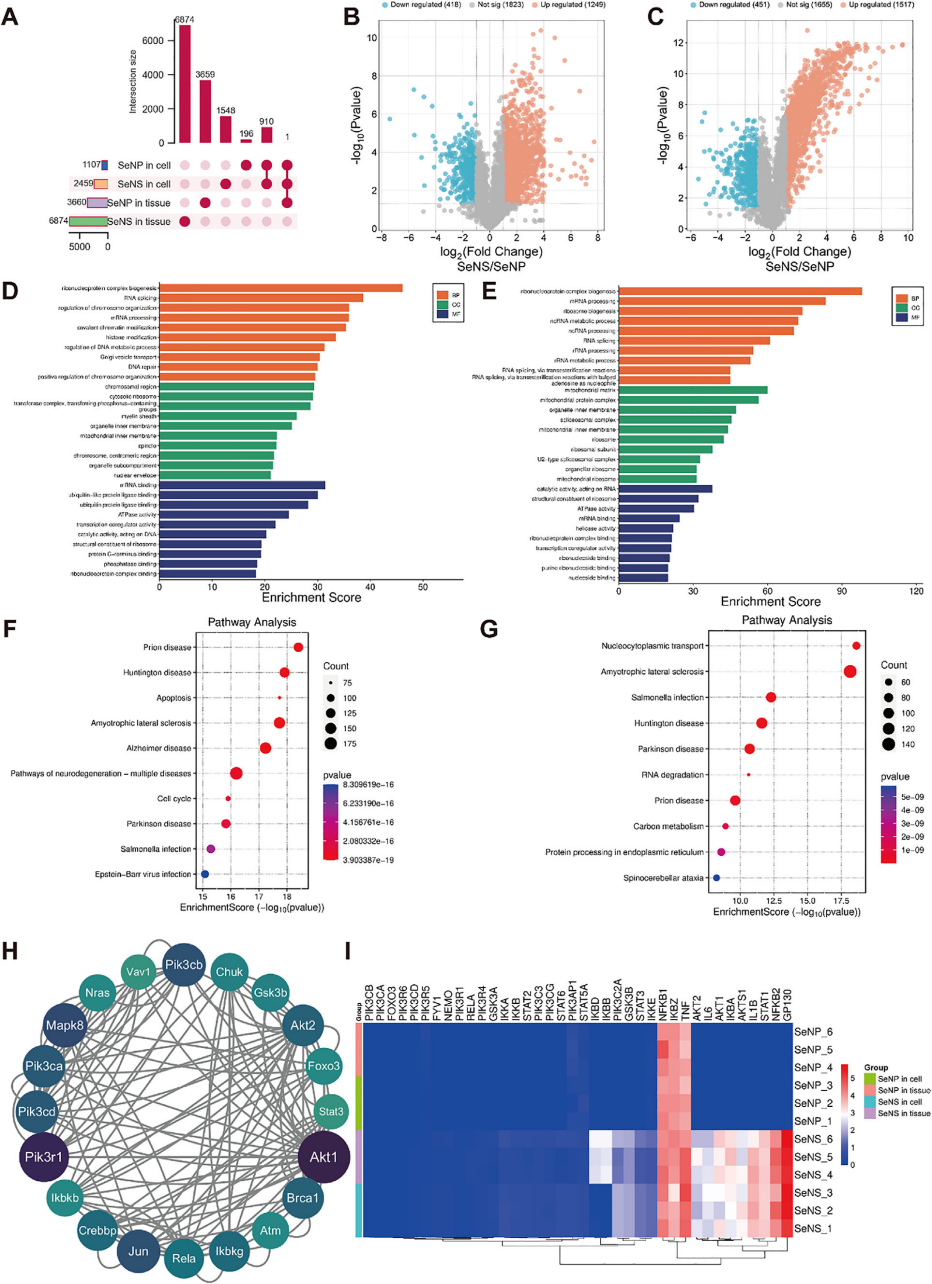
Proteomic analysis of SeNS and SeNP protein coronas. A) UpSet plot showing shared proteins in cells and tissues. B,C) Volcano plots in cells B) and colon tissues C). D,E) GO enrichment analysis of the differentially expressed proteins in cells D) and colon tissues E). F,G) KEGG pathway enrichment analysis of the differentially expressed proteins in cells F) and colon tissues G). H) The PPI network of the top 20 UC‐related proteins in the SeNS protein corona in cells. I) Heatmap of the protein levels. (*p < 0.05, **p < 0.01, ***p < 0.001, ****p < 0.0001; data are expressed as mean ± standard deviation, *n* = 3 independent experiments, student‐t test, and one‐way ANOVA).

The sequential window acquisition of all theoretical fragment ions (SWATH) quantitative proteomic method was used to identify the quantities and types of different proteins in the protein coronas formed by the SeNSs and SeNPs (abundance ratio > 1.5‐fold; *p* < 0.05). In RAW264.7 cells, the protein coronas formed by the SeNSs and SeNPs contained 2459 and 1107 proteins, respectively, whereas in colon tissue, the corresponding values were 6874 and 3660 proteins, respectively (**Figure** **6**A). The greater numbers of proteins adsorbed by the SeNSs and SeNPs in the colon may be explained by the high pH of the colon tissue.^[^ ^42^ ^]^ The concentration of the SeNS protein corona (287.98 µg mg^−1^ Se) was greater than that of the SeNP protein corona (263.81 µg mg^−1^ Se) (Figure S19, Supporting Information). SeNSs can interact with more proteins due to their larger surface area.^[^ ^43^ ^]^

In the cells, 1249 proteins were present at higher concentrations, and 418 proteins were present at lower concentrations in the SeNS protein corona than in the SeNP corona (Figure 6B). Similarly, in the colon tissues, 1517 proteins were present at higher concentrations, and 451 were present at lower concentrations in the SeNS protein corona than in the SeNP protein corona (Figure 6C). The differences in shape and protein interaction areas between SeNSs and SeNPs triggered the adsorption of different proteins (Figures S20, S21, Supporting Information).^[^ ^42^ ^]^ Moreover, the proteins absorbed are involved in different cellular pathways, contributing to the biological activity of SeNSs and SeNPs.^[^ ^44^ ^]^

Next, the proteins differentially expressed between the SeNS and SeNP protein coronas formed in macrophage cells and colon tissues were subjected to Gene Ontology (GO) enrichment analysis. In cells, SeNSs predominantly adsorbed proteins related to mRNA binding, ubiquitin‐like protein ligase binding, and ubiquitin‐protein ligase binding. These proteins were distributed in the chromosome, cytoplasmic ribosome, and phosphotransferase complex regions of the cell and were involved mainly in the biogenesis of ribonucleoprotein complexes, RNA splicing, and the regulation of chromosome organization (Figure 6D).

In contrast, the proteins differentially expressed between the SeNS and SeNP protein coronas in the colon tissue were located mainly in the mitochondrial matrix, mitochondrial protein complex, and inner organelle membrane. These proteins were associated with molecular functions such as RNA catalytic activity, ribosome structural component, and ATPase activity (Figure 6E) and were primarily involved in the biogenesis of ribonucleoprotein complexes, mRNA processing, and ribosome biogenesis.

Compared with SeNPs, SeNSs may alleviate inflammation in cells by affecting mRNA stability and translation processes and regulating protein and ribosome functions.^[^ ^45^, ^46^ ^]^ In the colon, SeNSs may reduce inflammation by modulating cellular immune responses and mitochondrial metabolism in epithelial cells.^[^ ^47^ ^]^ The differences in the protein coronas formed by SeNSs in the cellular and colonic environments may be due to the different cell types present in these physiological environments.^[^ ^44^ ^]^

An enrichment analysis using the Kyoto Encyclopedia of Genes and Genomes (KEGG) pathway database revealed that the differentially expressed proteins between the SeNS and SeNP protein coronas that formed in macrophages were predominantly associated with pathways such as prion diseases, Huntington's disease, and apoptosis (Figure 6F). Additionally, the differentially expressed proteins between the SeNS and SeNP protein coronas that formed in the colon were associated mainly with cell pathways related to nucleocytoplasmic transport, amyotrophic lateral sclerosis, and *Salmonella* infection (Figure 6G). All the identified cellular pathways were associated mainly with inflammatory responses.^[^ ^48^, ^49^, ^50^, ^51^ ^]^ The upstream modulators of neurodegenerative diseases, including prion diseases, Huntington's disease, and amyotrophic lateral sclerosis, are involved in inflammatory responses.^[^ ^48^, ^50^, ^51^ ^]^ Moreover, inflammatory responses and budding are typical features of apoptosis and key pathogenic mechanisms in *Salmonella* infection.^[^ ^52^, ^53^ ^]^

The development of inflammation relies on protein‐ and RNA‐dependent nucleocytoplasmic transport, which is important for activating signaling pathways and effector molecules.^[^ ^54^ ^]^ These results suggest that the differentially expressed proteins in the SeNS protein corona are closely associated with inflammatory responses.

A comparative analysis of the UC‐related proteins identified in the DisGeNET database and the differentially expressed genes in the SeNS protein corona revealed that 1582 UC‐related proteins were present in the SeNS protein corona in RAW264.7 cells, whereas only 799 among them were present in the SeNP protein corona. In the colon tissues, the SeNS protein corona contained 1565 UC‐related proteins, and 802 shared proteins appeared in the SeNP protein corona (Figure S22, Supporting Information). These results indicate that SeNSs can absorb more types of proteins than can SeNPs, and the protein types shared by SeNSs and SeNPs are similar in different environments.

As shown in Figures S23–S28 (Supporting Information), UC‐related proteins in the protein corona were used to construct a protein‒protein interaction (PPI) network with STRING. Proteins related to the AKT/PI3K pathway were found only in the SeNS protein corona, with the top 20 proteins ranked by the PPI score (Figure 6H). The proteins with the highest scores found in cells and colon tissues were ribosomal proteins and tumor necrosis factor (Figures S25, S28, Supporting Information). Proteins with high PPI scores are crucial participants in the PPIs reported in the network.^[^ ^55^ ^]^ The identification of the key functional proteins that reduce inflammatory responses in the SeNS protein corona could reveal the potential molecular mechanisms underlying the anti‐inflammatory effects of SeNSs. The PI3K/AKT signaling pathway is a major regulator of inflammation.^[^ ^56^ ^]^ Activation of the PI3K/AKT pathway promotes the activation and migration of inflammatory cells, thus increasing the release of proinflammatory cytokines from these cells.^[^ ^57^ ^]^ Moreover, proinflammatory cytokines can activate the NF‐κB signaling pathway. Once activated, phosphorylated NF‐κB proteins are translocated to the nucleus, where they amplify the inflammatory response.^[^ ^37^ ^]^ The SeNS protein corona contained many proteins involved in the AKT/PI3K and NF‐κB signaling pathways, which could modulate these pathways and contribute to the reduction in inflammation in individuals with UC. Figure 6I shows the proteins related to the AKT/PI3K and NF‐κB signaling pathways in the SeNS and SeNP protein coronas. Some NF‐κB pathway‐related proteins were found in both the SeNS and SeNP protein coronas, whereas proteins related to the AKT/PI3K pathway were found only in the SeNS protein corona. Notably, GP130, a membrane receptor protein of the AKT/PI3K pathway,^[^ ^58^ ^]^ was highly enriched in the SeNS protein corona, which indicated a strong interaction between SeNSs and GP130. The GP130 in the SeNS protein corona comes from both the gastrointestinal lumen during inflammation and cell membrane fragments adhered to SeNSs (Figure 3A; Figure S3, Supporting Information).^[^ ^59^ ^]^ The membrane fragments were collected when SeNSs were isolated, as the cell membrane was disrupted during the process. These results suggest that the absence of AKT/PI3K pathway‐related proteins in the SeNP protein corona may limit the ability of SeNPs to regulate the AKT/PI3K pathway, thereby weakening their ability to inhibit cellular inflammatory responses. Thus, the SeNSs may have greater anti‐inflammatory activity and effectiveness in treating UC.

### 2.5 Modulatory Effects of SeNSs and SeNPs on the AKT/PI3K and NF‐κB Signaling Pathways

**Figure 7:**
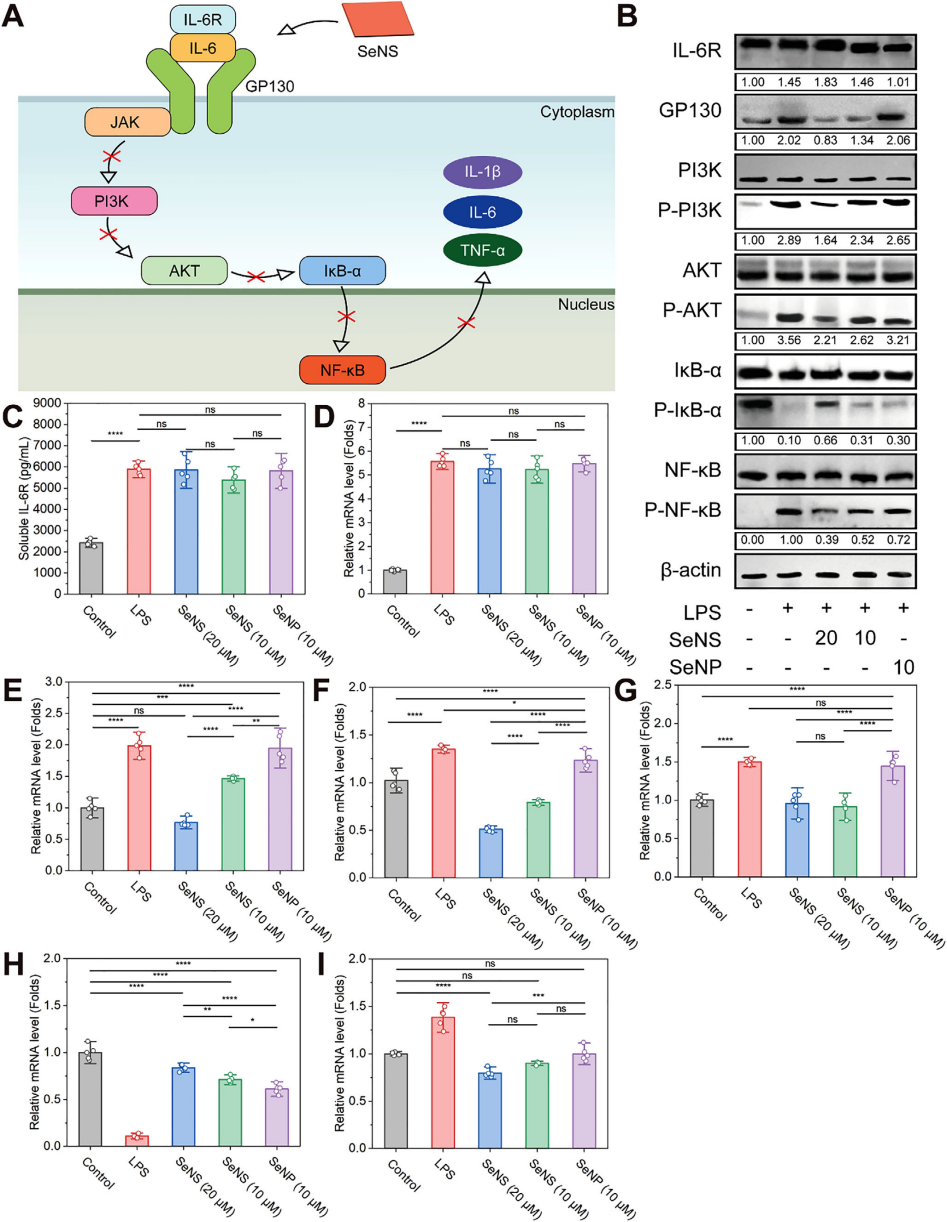
Anti‐inflammatory mechanism of SeNSs. A) Schematic diagram of SeNS‐mediated inhibition of the AKT/PI3K and NF‐κB signaling pathways. B) Western blot analyses of PI3K/AKT and NF‐𝜅B signaling pathway components in RAW264.7 cells incubated with different preparations for 14 h. C) Concentration of soluble IL‐6R measured by ELISA. D–I) mRNA expression levels of IL‐6R D), GP130 E), PI3K F), AKT G), IκB‐α H), and NF‐κB I). (*p < 0.05, **p < 0.01, ***p < 0.001, ****p < 0.0001; data are expressed as mean ± standard deviation, *n* = 5 independent experiments, student‐t test, and One‐way ANOVA).

Protein expression analyses were performed in RAW264.7 cells to verify that the SeNSs inhibited the AKT/PI3K and NF‐κB pathways (**Figure** **7A**). Western blot results revealed that compared with LPS treatment, SeNSs did not affect the expression of membrane‐bound IL‐6 receptor (mIL‐6R), inhibited GP130 expression, and decreased the levels of phosphorylated PI3K, AKT, and NF‐κB. However, SeNSs increased the level of the phosphorylated IκB‐α protein (Figure 7B; Figure S29, Supporting Information). IL‐6R can specifically bind to IL‐6 to form a complex that interacts with GP130 and activates the AKT/PI3K signaling pathway.^[^ ^58^ ^]^ GP130 is a common β‐receptor for the IL‐6 family of cytokines and plays a critical role in maintaining the inflammatory response.^[^ ^60^, ^61^, ^62^ ^]^

In previous studies, the inhibition of IL‐6R reduced the activation of GP130, leading to anti‐inflammatory activity.^[^ ^63^ ^]^ The above results confirmed that the inhibitory effect of SeNSs on GP130 expression was not due to the suppression of IL‐6/IL‐6R expression but rather to the direct inhibitory effect of SeNSs on GP130.^[^ ^64^ ^]^ IL‐6R is a cytokine involved in inflammation and immune responses and consists of membrane‐bound (mIL‐6R) and soluble (sIL‐6R).^[^ ^58^ ^]^ ELISA results indicated that the level of sIL‐6R was not influenced by SeNSs, similar to that of mIL‐6R (Figure 7C). The changes in the mRNA expression levels were consistent with those in the protein levels (Figure 7D–I), further confirming the inhibitory effects of SeNSs on the AKT/PI3K and NF‐κB pathways and their significant anti‐inflammatory activity. A similar anti‐inflammatory mechanism of SeNSs was observed in the colon tissue of UC mice subjected to different treatments (Figure S30, Supporting Information).

Sander et al. reported that the absence of the GP130 protein could prevent severe colitis in mice by reducing mucosal inflammation and local cytokine, chemokine, and adhesion molecule expression.^[^ ^65^ ^]^ Chrysin (5,7‐dihydroxyflavone) also alleviated DSS‐induced UC in mice by reducing GP130 expression and inhibiting the NF‐κB and PI3K/AKT pathways, which is in agreement with our results.^[^ ^66^, ^67^ ^]^ The SeNS‐mediated regulation of different proteins depends on the components of the protein corona. Here, SeNSs not only inhibited the expression of GP130 but also suppressed the PI3K/AKT and NF‐κB signaling pathways. Notably, GP130 expression was not reduced in the SeNP group, suggesting that SeNSs directly inhibited GP130. In subsequent experiments, the interaction between SeNSs and GP130 was further explored.

### 2.6 Interaction of SeNSs with GP130

**Figure 8:**
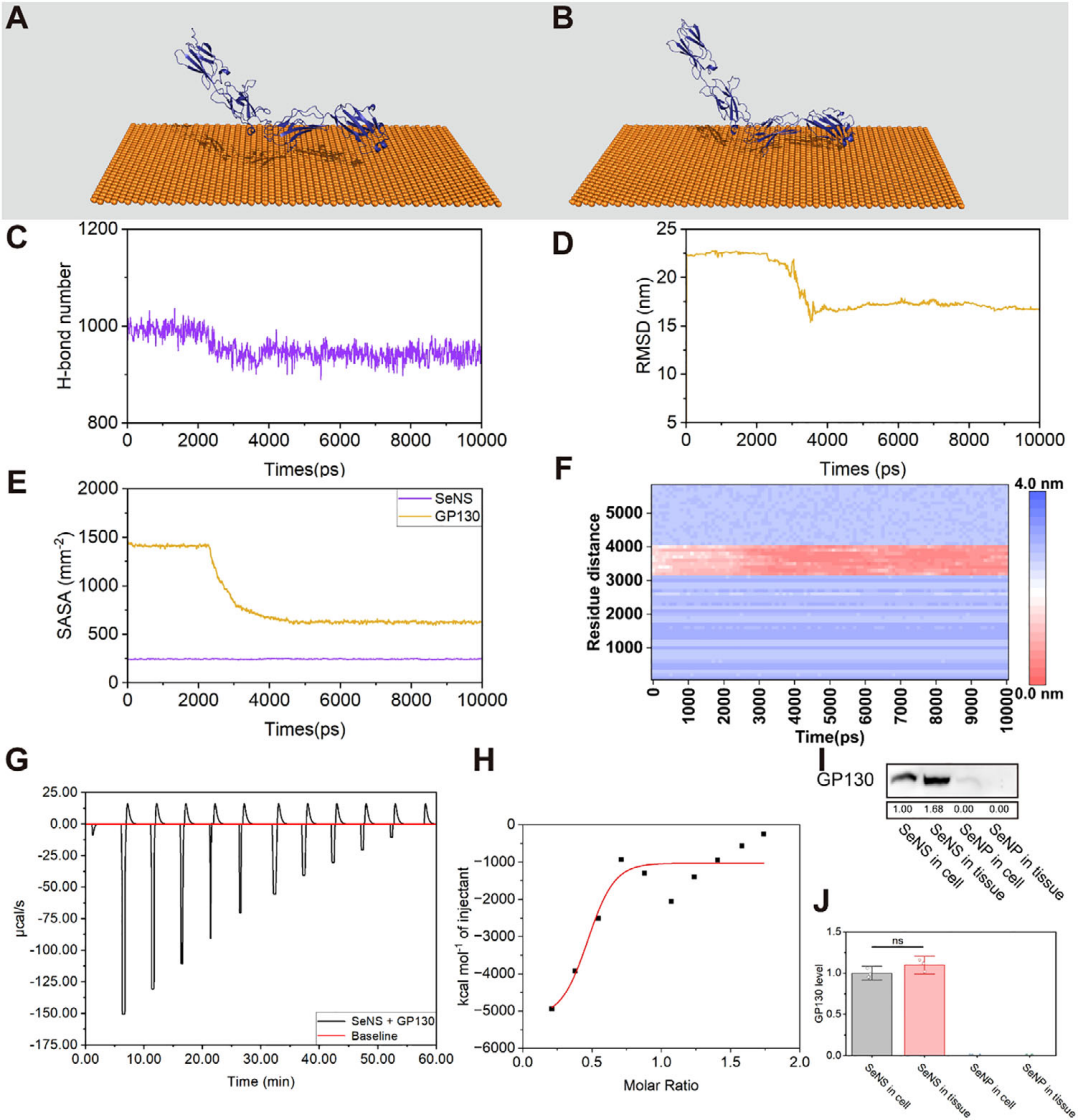
Interaction between GP130 and the SeNS. A,B) Visualized structures of the SeNS and GP130 at 0 ps A) and 10 000 ps B). C) Temporal evolution of the number of hydrogen bonds. D) RMSD values of GP130 as a function of the simulation time. E) SASA of GP130 and the SeNS. F) Distance between the residue of GP130 and the SeNS from 0 to 10 000 ps. G) Raw heat data obtained after injecting the SeNSs into the GP130 solution. H) Binding isotherms generated by plotting the areas under each peak in (G) against the SeNS‐to‐GP130 molar ratio. I,J) Western blot analysis of GP130 expression in the protein corona I) and its quantification J). (*p < 0.05, **p < 0.01, ***p < 0.001, ****p < 0.0001; data are expressed as mean ± standard deviation, *n* = 3 independent experiments, student‐t test, and one‐way ANOVA).

A change in the shape of a nanoparticle can modify its capacity to bind to different proteins.^[^ ^68^ ^]^ Factors such as the spatial hindrance of the protein, the surface charge of the material, and hydrophobicity affect the interactions of SeNSs and SeNPs with GP130.^[^ ^69^ ^]^ Compared with SeNPs, SeNSs have a larger surface area and more active surface sites, thus reducing steric hindrance and promoting interaction with GP130.^[^ ^69^ ^]^ Molecular dynamics simulations were used to explore the mechanism of the interaction between GP130 and SeNSs. First, a SeNS model was constructed using the unit cell of t‐Se (#mp‐14) as the basic unit, with the positions of each selenium atom fixed at the bottom of the model. The structure of the GP130 monomer (PDB ID: 3L5H) was obtained for separate molecular simulations and a molecular simulation system for interaction with SeNS.^[^ ^70^ ^]^ The single GP130 protein simulation system was utilized as a control group to evaluate the stability of the protein (Figure S31, Supporting Information). In the 1000‐ps molecular dynamics simulation of the single GP130 protein, the root mean square deviation (RMSD) was ± 0.2 nm (Figure S32, Supporting Information). The number of hydrogen bonds and the solvent‐accessible surface area (SASA) varied around 1022 and 241, respectively (Figures S33, S34, Supporting Information). The above results indicated the suitability of this approach for simulating interaction with the SeNS. The molecular dynamics simulation results revealed that GP130 moved toward the bottom of the simulation system and its distance to the SeNS decreased at 10 000 ps compared with 0 ps of simulation time (**Figure** **8**A,B). From 2270 to 3630 ps, the number of hydrogen bonds between GP130 and the solution slowly decreased (Figure 8C). This reduction indicated that the protein might have undergone displacement or conformational changes.^[^ ^71^ ^]^ Notably, from 2270 to 3630 ps, the RMSD of GP130 also decreased from 22.44 to 16.33 nm and then remained stable at 15.75 ± 0.2 nm from 3630 to 10 000 ps (Figure 8D). The results indicated that GP130 moved from 2270 to 3630 ps before gradually stabilizing.

Protein displacement can be caused by factors such as hydrophobic interactions, hydrogen bonding, and electrostatic forces between the nanomaterials and proteins in solution.^[^ ^69^ ^]^ The decrease in hydrogen bonding indicated that hydrogen bonding was not responsible for the interaction between the SeNS and GP130. The SASA was determined to evaluate the aggregation of GP130 with the SeNS and its contact area with the solvent.^[^ ^72^ ^]^ As shown in Figure 8E, the SASA of the SeNS and GP130 were recorded during the simulation. With the displacement of GP130, the SASA gradually decreased, indicating a reduction in the surface area of GP130 in contact with the solvent. The aggregation of GP130 with the SeNS suggested that the GP130–SeNS interaction was driven primarily by hydrophobic interactions. Consistent with Nishiyama's findings, after interacting with a 2D Si substrate, the protein‒solvent contact area decreased, demonstrating strong hydrophobic interactions between the Si substrate and the protein.^[^ ^73^ ^]^ Figure 8F shows the distance between the residue of GP130 and the SeNS in the 10 000‐ps simulation. The hydrophobic interaction between GP130 and the SeNS occurred within amino acid residues 3100–4100. Side chains with nonpolar or weakly polar amino acids have greater hydrophobicity and are more likely to interact with hydrophobic materials.^[^ ^74^ ^]^ Se atoms form fewer hydrogen bonds in biological environments and exhibit strong hydrophobicity.^[^ ^19^ ^]^ The 3100–4100 amino acid residues of GP130 are speculated to have many side chains with nonpolar or weakly polar amino acids.

An isothermal titration calorimetry (ITC) experiment was conducted to obtain detailed thermodynamic information, such as the binding enthalpy (∆H), binding entropy contribution (T∆S), and free energy change (∆G), during the SeNS–GP130 interaction via the equation Δ*G*  =  −*RTlnK_a_* =  Δ*H* − *T*Δ*S*.^[^ ^75^ ^]^ Representative calorimetric curves for the titration of GP130 with the SeNSs and SeNPs are shown in Figure 8G and Figure S35 (Supporting Information), respectively. In the ITC experiment, the heat released during each injection of the SeNSs into the GP130 was measured. The injection of the SeNSs into the buffer solution was used as a control to correct for the heat effects caused by the dilution of the nanoparticles. As shown in Figure 8H, the resulting values were plotted against the molar ratio of SeNS/GP130 and fitted to a site model via nonlinear least squares. During the SeNS‐GP130 interaction, the enthalpy change (ΔH), entropy contribution (TΔS), and free energy change (ΔG) were −4.14, −2.15, and −6.29 kcal mol^−^¹, respectively (Table S2, Supporting Information). Furthermore, the K_a_ value (2.13 × 10^−3^ m ^−^¹) and the number of binding sites (0.15 sites) for the SeNS–GP130 interaction were greater than those for the SeNP–GP130 interaction, where the K_a_ value was 1.35 × 10^−3^ M^−^¹ and the number of binding sites was 0.01 (Table S2, Supporting Information). The above results indicated that the interaction process between the SeNSs and GP130 was an exothermic reaction. Compared with the SeNPs, the SeNSs bound more easily to GP130. Exothermic binding of the SeNSs to GP130 indicated that the interaction between GP130 and the SeNSs might be driven by hydrogen bonding, van der Waals forces, hydrophobic interactions, and other forces, which was consistent with the results of the molecular dynamics simulation.^[^ ^76^ ^]^ The adsorption of proteins onto the surface of nanoparticles is a complex process involving multiple physicochemical interactions.^[^ ^77^ ^]^ Proteins with a greater affinity for nanoparticles can form a “hard corona” that is less affected by changes in the environment.^[^ ^78^ ^]^ In contrast, lower‐affinity binding results in a “soft corona” that is unstable and takes a long time to form.^[^ ^77^ ^]^ In this study, SeNS showed a stronger affinity for GP130 (binding free energy: −7.87 kcal mol^−1^) than the GP130‐specific inhibitor bazedoxifene (BZA) (binding free energy: −5.51 kcal mol^−1^) (Figure S36 and Table S3, Supporting Information). The results proved that GP130 not only formed the “hard corona” of SeNS but also specifically bound to SeNS. The SeNSs were able to adsorb GP130 at high levels in both colon tissues and cells. Consistent with the Western blot results for the protein corona, the concentration of GP130 was greater in the SeNS protein corona than in the SeNP protein corona (Figure 8I,J). The knockdown of GP130 expression and the inhibition of GP130 activity both effectively downregulate AKT signaling pathway, leading to anti‐inflammatory effects (Figures S37, S38, Supporting Information). However, the removal of GP130 protein from the SeNS protein corona (via polyethylene glycol modification to obtain PEG‐SeNS) weakened SeNS's anti‐inflammatory ability (Figure S37, Supporting Information). The above results confirm SeNS's key role in targeting and inhibiting GP130 expression during the anti‐inflammatory process (Figures S37, S38, Supporting Information).

### 2.7 Transcriptomic Analysis of UC

**Figure 9:**
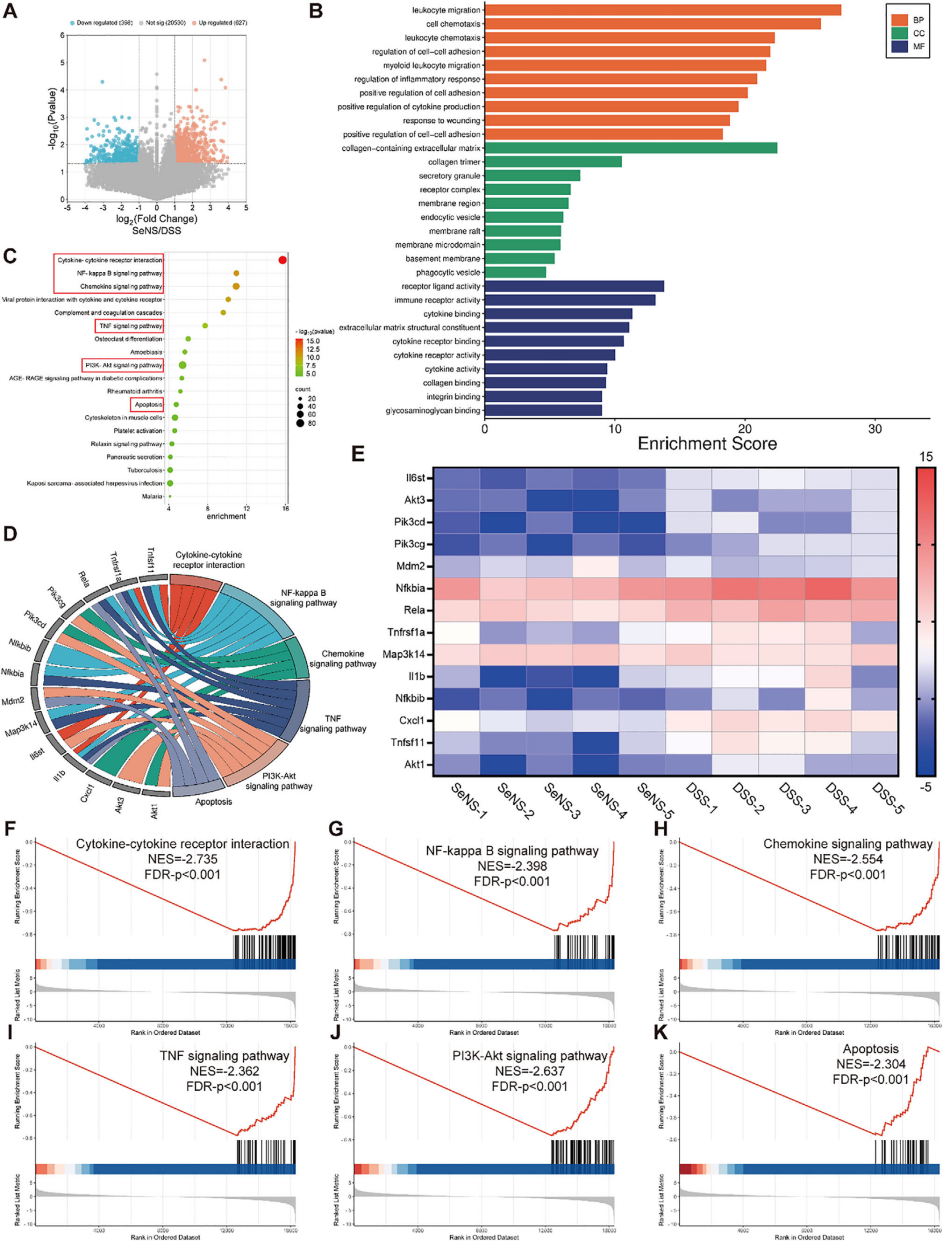
Transcriptomic sequencing analysis. A) Volcano plots comparing gene expression between the SeNS and DSS groups. B) GO enrichment analysis of differentially expressed genes in the DSS group compared with the SeNS group. C) KEGG pathway enrichment analysis of the differentially expressed genes between the DSS group and the SeNS group. D) Enrichment analysis of the differentially expressed genes. E) Heatmap depicting the differential expression of genes. F‒K) Gene set enrichment analysis (GSEA) of cytokine‒cytokine receptor interactions F), the NF‒κB signaling pathway G), the chemokine signaling pathway H), the TNF signaling pathway I), the PI3K‒AKT signaling pathway J), and pathways related to apoptosis (K). (*p < 0.05, **p < 0.01, ***p < 0.001, ****p < 0.0001; data are expressed as mean ± standard deviation, *n* = 5 independent experiments, student‐t test, and one‐way ANOVA).

Transcriptome sequencing of colon tissues from DSS‐induced acute UC model mice was performed to explore the regulatory effects of SeNSs on mRNA expression. Abnormal gene expression was compared among the following groups: SeNS group versus DSS group, SeNS group versus SeNP group, DSS group versus control group, and SeNP group versus DSS group. The volcano plots in **Figure** **9**A revealed that, compared with the DSS group, the SeNS group presented 627 upregulated genes and 396 downregulated genes. Comparative analysis of differentially expressed genes revealed distinct transcriptional profiles across the experimental groups (Figures S39–S41, Supporting Information). Compared with the SeNP group, the SeNS group presented 71 upregulated and 181 downregulated genes, indicating moderate transcriptional reprogramming. In contrast, compared with control mice, DSS‐treated mice presented substantial genomic alterations, with 702 upregulated and 832 downregulated genes, demonstrating the strongest differential expression pattern. Notably, SeNP administration partially reversed the DSS‐induced changes, resulting in 380 upregulated and 499 downregulated genes compared with those in the DSS group.

A Venn diagram of the differentially expressed genes in the colon tissues of the mice in the DSS, SeNS and SeNP groups versus the control group is shown in Figure S42 (Supporting Information). Among these groups, the SeNS group presented the fewest abnormally expressed genes, whereas the DSS group presented the most abnormally expressed genes.

GO enrichment analysis revealed that SeNS treatment alleviated UC by regulating genes involved in cell membrane components, extracellular matrix, and collagen trimer formation, thereby modulating immune‐related processes such as leukocyte migration, cell chemotaxis, cytokine secretion, cell adhesion, and signal transduction (Figure 9B). Compared with SeNPs, SeNSs specifically affected cytokine receptor binding, phospholipid synthesis, and lipid metabolic processes (Figures S43–S46, Supporting Information). These changes suggest that SeNSs may more effectively restore cellular function, modulate immune responses, and repair epithelial barriers in DSS‐induced colitis.

KEGG pathway enrichment confirmed that SeNSs regulate several dysregulated pathways in UC, such as cytokine–cytokine receptor interactions, NF‐κB activity, chemokine signaling, TNF and AKT/PI3K activity, and apoptosis (Figure 9C; Figure S47, Supporting Information). Notably, SeNSs, but not SeNPs, also modulated Th cell differentiation (Figures S48, S49, Supporting Information), a process regulated by AKT via mTORC signaling.^[^ ^79^ ^]^ Additionally, SeNSs regulated key dysregulated pathways (Figure 9D), such as the NF‐κB, TNF, and AKT/PI3K pathways, and modulated Th cell differentiation, contributing to immune regulation and anti‐inflammatory effects in UC (Figures S50, S51, Supporting Information). These findings suggest that SeNSs exert anti‐inflammatory effects by regulating Th cell differentiation and macrophage polarization (M1/M2), ultimately contributing to immune regulation.^[^ ^80^ ^]^

The expression levels of the differentially expressed genes in the different treatment groups are shown in Figure 9E and Figures S52–S54 (Supporting Information). Downregulation of the representative genes Il1b and Il6st was observed in the SeNS group compared with the SeNP group. This change might block the interaction between cytokine receptors and cytokines, leading to reduced secretion of proinflammatory cytokines, less tissue damage and cell death, and ultimately suppression of the inflammatory response.^[^ ^77^, ^81^, ^82^ ^]^

DSS treatment activated inflammatory pathways, including cytokine–cytokine receptor interactions and chemokine signaling (Figure S55, Supporting Information). Both SeNSs and SeNPs suppressed the above pathways (Figure 9F,G; Figures S56, S57, Supporting Information), whereas SeNSs also inhibited the NF‐κB, TNF, and AKT/PI3K signaling pathways and apoptosis‐related pathways (Figure 9H–K). SeNSs alleviated UC by modulating immune‐related processes, including cytokine secretion and cell adhesion, and enhancing cellular function and epithelial barrier repair. The potent anti‐inflammatory effects of SeNSs were attributed to the ability of SeNSs to adsorb proteins associated with these pathways, forming a protein corona that modulates signaling cascades. These findings underscore the complex and multitarget anti‐inflammatory mechanisms of SeNSs in the treatment of UC.

## 3 Conclusions 

In this work, ultrathin SeNSs were developed for effective anti‐inflammatory treatment, and their therapeutic efficacy in a DSS‐induced acute UC mouse model was observed. The SeNSs adhered to the surface of the macrophages and exhibited a high cellular uptake rate. Moreover, SeNSs inhibited the secretion of the cytokines IL‐1β, IL‐6, and TNF‐α by macrophages, reduced the production of the harmful oxygen free radical NO, and downregulated the expression of iNOS. The bioactivity of SeNSs was closely associated with its protein corona. The SeNS protein corona is formed by the adsorption of many proteins. Proteomic analysis revealed that the composition of the SeNS protein corona in macrophages was different from that in colon tissue. The SeNS protein corona in macrophages was involved mainly in ribosome‐related biological processes. However, in colon tissue, it was associated mainly with mitochondrial metabolism. The SeNS protein corona contained UC‐related proteins, such as AKT/PI3K and NF‐κB pathway proteins, which were absent from the SeNP protein corona. The SeNSs inhibited the AKT/PI3K and NF‐κB pathways and bound to the cytokine receptor GP130 through hydrophobic interactions, reducing its expression. This inhibitory effect reduced inflammatory signaling, downregulated the expression of inflammation‐related genes, and alleviated the inflammatory response.

In DSS‐induced acute UC model mice, transcriptome analysis revealed that SeNSs suppressed the inflammatory response in colonic tissues by modulating multiple cellular pathways, including cytokine‒cytokine receptor interactions, the NF‒κB signaling pathway, the chemokine signaling pathway, the TNF signaling pathway, the AKT‒PI3K signaling pathway, and pathways related to apoptosis. These actions reduced congestion, edema, and inflammatory cell infiltration in colon tissues, thus promoting recovery from colon damage and leading to the effective treatment of UC. The results of both in vitro and in vivo experiments confirmed the anti‐inflammatory activity of the SeNSs. SeNSs are powerful anti‐inflammatory agents that may be used to overcome the challenges in UC therapy. This study highlights the key role of the nanoparticle protein corona in regulating protein expression in biological environments. The successful application of SeNSs may pave the way for the precise and efficient treatment of inflammatory diseases via protein corona modulation in the future.

## 4 Experimental Section

### Materials

Na_2_SeO_3_, ascorbic acid, isopropyl alcohol, guanidine hydrochloride, 3‐(4,5‐dimethyl‐2‐thiazolyl)‐2,5‐diphenyl‐2‐H‐tetrazolium bromide, hematoxylin, and eosin staining solutions were purchased from Sigma‒Aldrich (Missouri, USA). Tween 20 and lipopolysaccharide (LPS) were obtained from Thermo Fisher Scientific (Massachusetts, USA). Newborn calf serum (16010159), RIPA lysis buffer, and Dulbecco's modified Eagle's medium were obtained from Thermo Fisher Scientific (Massachusetts, USA). A 4% paraformaldehyde fixative solution (P0099), immunostaining permeabilization buffer with Triton X‐100 (P0096), a protease inhibitor, a phosphatase inhibitor, 4,6‐diamino‐2‐phenyl indole (C1005), and a BCA protein assay kit were acquired from Shanghai Beyotime Biotech. IL‐6, IL‐1β, TNF‐α, and IL‐6R ELISA kits were obtained from Proteintech (Illinois, USA). C6 was acquired from Macklin. The goat anti‐rabbit IgG H&L (ab6702), goat anti‐mouse IgG H&L (ab6708), anti‐iNOS antibody [EPR16635] (ab178945), anti‐GP130 antibody [EPR2405238] (ab97505), anti‐IL‐6R antibody, anti‐PI3K p85 alpha (phospho Y607) antibody (ab182651), anti‐PI3K alpha antibody [EPR18702] (ab191606), anti‐AKT1 (phospho S473) antibody [EP2109Y] (ab81283), anti‐AKT1 antibody [BLR245L] (ab314110), anti‐IKB alpha (phospho S32) antibody [EPR3148] (ab92700), anti‐IKB alpha antibody [E130] (ab32518), anti‐NF‐κB p65 antibody [E379] (ab32536), anti‐NF‐κB p65 (phospho S536) antibody [EP2294Y] (ab76302), and anti‐beta actin antibody [AC‐15] (ab6276) used in Western blot assays were purchased from Abcam (Cambridge, UK). All reagents were of analytical grade and were not purified prior to use.

### Synthesis of SeNSs

A total of 0.01 g of Na_2_SeO_3_ and 0.03 g of ascorbic acid were dissolved in 5 mL of isopropanol. The pH of the solution was adjusted to 7.4. The solution was subjected to ultrasonic treatment at 0 °C for 4 h to obtain the SeNS solution. The SeNS solution was centrifuged at 12 000 rpm for 1 h. The supernatant was discarded, and the precipitate was collected. The precipitate was washed with isopropanol three times. After washing, the resulting SeNS precipitate was collected for further use.

### Synthesis of SeNPs

First, 0.01 g of Na_2_SeO_3_ and 0.03 g of ascorbic acid were dissolved in 5 mL of ultrapure water. The pH of the solution was adjusted to 7.4. A 0.1% solution of Tween 20 was added to prevent aggregation of the nanoparticles. The solution was stirred at 0 °C for 3 h to obtain the SeNPs. The SeNP‐containing solution was centrifuged at 12 000 rpm for 1 h, and the precipitate was collected. After three washes, the SeNP precipitate was used as a control.

### Synthesis of SeNS@C6 and SeNP@C6

SeNS@C6 was obtained by dissolving 0.01 g of Na_2_SeO_3_, 0.005 g of C6, and 0.03 g of ascorbic acid in 5 mL of isopropanol, followed by ultrasonic treatment at 0 °C for 4 h. The resulting SeNS@C6 was separated by centrifugation at 12 000 rpm for 1 h. After three washes, the SeNS@C6 precipitate was stored at 4 °C in the dark.

SeNP@C6 was prepared by dissolving 0.01 g of Na_2_SeO_3_, 0.005 g of C6, 0.1% Tween 20, and 0.03 g of ascorbic acid in 5 mL of ultrapure water and stirring at 0 °C for 3 h. SeNP@C6 was collected via a method similar to that used for SeNS@C6. After centrifugation, the precipitate was stored in the dark.

### Characterization of SeNSs and SeNPs

A total of 0.01 g of SeNS or SeNP precipitate was dissolved in 1 mL of ultrapure water. The particle size distribution and ς potential were measured with a Malvern Zetasizer Nano ZS analyzer (Worcestershire, UK).

Mica sheets were impregnated with a 0.01% guanidine hydrochloride aqueous solution and dried to obtain positively charged modified mica sheets. The SeNS and SeNP aqueous solutions were dripped onto the mica sheets. After drying, the thicknesses of the SeNSs and the heights of the SeNPs were measured via AFM (Oxford MFP‐3D Bio).

Typical images of SeNSs and SeNPs were obtained via a TEM (JEM‐1230) at an accelerating voltage of 200 kV. A high‐resolution transmission electron microscope (HRTEM) (FEI Tecnai G2 F30) with an accelerating voltage of 300 kV was used to obtain high‐resolution images of the SeNSs and SeNPs.

A high‐resolution confocal Raman microscope (HORIBA Lab RAM HR800) was used to obtain the Raman spectra of the SeNSs and SeNPs at room temperature.

XPS results for the SeNSs and SeNPs were obtained via a PHI‐5000 Versa Probe II instrument (ULVAC‐PHI). The full survey scan of the samples ranged from 1300 to 0 eV. Monochromatic Al Kα radiation was used. In addition, core‐level scans of the Se 3d regions were performed for the SeNSs and SeNPs. The XRD patterns of the SeNSs and SeNPs were determined via a Bruker D8 instrument.

The SeNS and SeNP powders were previously lyophilized, and scans were performed at a scan speed of 1 min^−1^ with a scan voltage of 40 kV and a current of 40 mA.

### Cell Culture

The murine RAW264.7 macrophages were obtained from the China Center for Type Culture Collection (CCTCC) (Shanghai, China). The cells were cultured in DMEM supplemented with 10% FBS and maintained in a 5% CO_2_ humidified incubator at 37 °C. After 1 day of incubation, the cells were prepared for use.

### Cytotoxicity Assay

An MTT assay was used to assess cell viability following different treatments to evaluate the cytotoxicity of the SeNSs and SeNPs. RAW264.7 cells were seeded in 96‐well plates. The cells were incubated with serum‐free DMEM at 37 °C for 4 h. The SeNSs and SeNPs at final concentrations of 0, 5, 10, 20, and 40 µm were then added to the culture medium. The cells were cocultured with SeNSs or SeNPs for 24 h. After the incubation, the supernatants in the 96‐well plates were discarded, and 3‐(4,5‐dimethyl‐2‐thiazolyl)‐2,5‐diphenyl‐2‐H‐tetrazolium bromide (MTT) and DMSO were added. The absorbance of each sample at 570 nm was measured using a microplate reader.

### In Vitro Cellular Uptake Assay

Uptake experiments were performed using RAW264.7 cells to study the cellular uptake of SeNSs and SeNPs. RAW264.7 cells were pretreated with serum‐free medium and seeded into laser confocal dishes for the cellular uptake experiment. The interference of serum with the cells was eliminated by the use of serum‐free medium. SeNS@C6 and SeNP@C6 at a concentration of 10 and 20 µm, respectively, were incubated with the cells for 2 h. After incubation, the medium was discarded, and the cells were washed three times with PBS. Then, 4% paraformaldehyde was used to fix the cells. After the cells were stained with DAPI and DiI, they were imaged via CLSM.

Inductively coupled plasma‒mass spectrometry (ICP‒MS) was used to measure the cellular uptake of the SeNSs and SeNPs. Briefly, RAW264.7 cells were coincubated with SeNSs or SeNPs at concentrations of 10 and 20 µm, respectively. The cells were dissolved in nitric acid and heated in a microwave for 2 h. The resulting solution was then diluted and analyzed via ICP‒MS (Shimadzu ICP‒2030).

### In Vivo Cell Uptake Assay

The Se contents in the heart, liver, spleen, colon, and kidney of the mice were measured via ICP‒MS to evaluate the absorption of the SeNSs and SeNPs in vivo. C57BL/6 mice aged 5–7 weeks were administered SeNSs or SeNPs by gavage at a concentration of 1 or 2 mm. At 0, 4, 12, and 24 h after dosing, the mice were anesthetized and euthanized. Heart, liver, spleen, kidney, and colon tissues were collected for analysis. The collected tissues were dried at 50 °C for 4 h. The samples were then digested with nitric acid and microwave‐digested for 2 h before measurement. The resulting solution was analyzed via ICP‒MS (Shimadzu ICP‒MS‐2030).

### Nitric Oxide Assay

NO production was measured by quantifying nitrite levels. Nitrite is a stable oxidation product of NO. RAW264.7 cells were first seeded into 96‐well plates. LPS (1 µm) and different concentrations of SeNSs or SeNPs (0, 5, 10, 20, and 40 µm) dissolved in serum‐free cell culture medium were added to the cells. After 24 h of incubation, the supernatant was mixed with 100 µL of Griess reagent (1% sulfanilamide, 0.1% N‐[naphthyl]ethylenediamine dihydrochloride, and 5% phosphoric acid) and incubated for 10 min at room temperature. The absorbance was measured using a microplate reader.

### ELISA

Cytokine levels were measured to assess the inflammatory response of cells and tissues, and ELISA was performed to measure cytokine levels in cells and tissues treated with SeNSs and SeNPs. RAW264.7 cells were treated with 10 ng mL^−1^ LPS, SeNSs or SeNPs for 14 h. The culture supernatant was subsequently collected for analysis. Cytokine levels in mouse colon tissues were also measured via IL‐1β, IL‐6, and TNF‐α ELISA kits. Colon tissues were lysed with tissue lysis buffer and centrifuged. The supernatant was collected for subsequent analysis.

### Western Blot Analysis

RAW264.7 cells were first incubated with LPS and different concentrations of SeNSs (10 and 20 µm) or SeNPs (10 µm) at 37 °C for 14 h. Next, the cells were collected and processed for protein extraction. The protein concentration was determined via a BCA protein assay kit. Protein samples were mixed with 5× loading buffer and then boiled at 100 °C for 5 min. SDS‒PAGE (12.5% gels) was used to separate the protein samples. The separated proteins were transferred onto a PVDF membrane. The PVDF membrane was blocked with 5% skim milk at room temperature for 1.5 h. The blocked PVDF membrane was incubated with the primary antibody (1:1000) overnight at 4 °C. The membrane was washed three times with PBST, followed by incubation with a goat anti‐rabbit/mouse IgG (H+L)–HRP conjugate (1:1000). Finally, Western blot images were obtained via a Western blot imaging system and analyzed with Image J.

### Proteomic Analysis of Protein Coronas

The SeNSs and SeNPs were coincubated with RAW264.7 cells and colon tissue for 2 h. The SeNS and SeNP protein coronas were then separated via centrifugation, extracted, and analyzed by HPLC–MS. The quantified proteomic profiles were obtained using a SWATH mass spectrometer. The data were analyzed using the SRplot platform.

### In Vivo Anti‐Inflammatory Activity Assays

Young, healthy female C57BL/6 mice (5–6 weeks old) were housed in a barrier environment under a 12‐h light/dark cycle with ad libitum access to food and water. A total of 25 mice were randomly assigned to five groups (*n* = 5): a) the control group, b) model group, c) 1 mm SeNS treatment group, d) 2 mm SeNS treatment group, and e) 1 mm SeNP treatment group. Except for the control group, which was provided only sterile water, all the other groups were treated with 2.5% DSS dissolved in sterile water for 7 consecutive days to induce colitis. The mice in the SeNS and SeNP treatment groups were orally administered different concentrations of SeNSs (0.01 mL g^−1^ day^−1^) or SeNPs (0.01 mL g^−1^ day^−1^) daily throughout the experiment. During the DSS induction period, the disease activity index (DAI) and body weight of the mice were monitored.

On day 7 of the experiment, the mice were anesthetized and euthanized, and colon samples were collected for colon length measurement, gene expression analysis, cytokine concentration assays, and histological evaluation. Fecal occult blood was detected using a fecal occult blood qualitative detection kit (Shanghai YuanYe Biotechnology Co., Ltd.).

### Ethical Statement

All animal experiments were approved by the Department of Science and Technology of Guangdong Province, China, and conducted in accordance with the Guidelines for the Care and Use of Laboratory Animals. The laboratory was accredited under the license number SYXK(Yue)2022‐0136. All efforts were made to minimize animal suffering.

### RNA Sequencing of Mouse Colon Tissue

Total RNA was extracted from the colon tissue mice subjected to different treatments (2.5% DSS, 1 mM SeNS, or 1 mm SeNPs) via TaKaRa RNAiso Plus reagent. Majorbio (Shanghai, CHN) conducted library construction and RNA sequencing. mRNA was purified via magnetic beads conjugated with oligonucleotides (dTs). cDNA was synthesized by reverse transcription initiated with random hexamers and second‐strand cDNA synthesis. The final library was amplified to generate DNA nanoballs (DNBs), which were subsequently loaded onto patterned nanoarrays. Sequencing was performed on the Illumina platform, and the data were filtered and aligned via Trimmomatic and Bowtie2. The data were analyzed using the SRplot platform.

### qPCR

RNA was extracted from mouse colon tissue and RAW264.7 cells via TaKaRa RNAiso Plus reagent. The obtained RNA was reverse transcribed with a PrimeScript RT‒PCR kit. The primer sequences are provided in Table S3 (Supporting Information). The data were processed using the Bio‐Rad iQ5 standard‐edition optical system. The assay was performed in duplicate and repeated three times on separate days.

### Molecular Dynamics Simulation

The SeNS was modeled via single‐layer repeating t‐Se unit cells (#mp‐14). The SeNS model used in this study consisted of 5400 selenium atoms. GP130 (PDB ID: 3L5H) was initially placed 2 nm above the SeNS surface. The system box size was set to 13.46 × 23.11 × 12.18 nm^3^. Water molecules and 0.15 m NaCl were added to simulate physiological conditions.

The GROMACS software package with the CHARMM36 force field was used for the simulations. The SeNS atoms were fixed during the simulation. PyMOL software was used for analysis and visualization of the simulation results. The TIP3P water model was applied to treat the water molecules. During the simulation, a 2.0‐fs time step was used, with data collected every 10 ps. The LINCS algorithm was applied to calculate van der Waals (vdW) interactions with a cutoff distance of 1.2 nm. The system ran for 10 000 ps.

### Statistical Analysis

Each experiment was performed at least three times, and all the data are presented as the means ± standard deviations (SDs). The statistical analysis was performed via Origin (2025b), and the results were evaluated via analysis of variance (ANOVA). P values of 0.05 or less were considered statistically significant. The differences were considered significant when the *p* values were * < 0.05, ** < 0.01, *** < 0.001, and **** < 0.0001.

## Author Contributions

Conceptualization: Dingyi Shen and Youzhi Tang. Methodology: Dingyi Shen, Li Gong, and Wei Yang. Investigation: Dingyi Shen, Li Gong, Jiaqi Luo, and Zhen Jin. Visualization: Dingyi Shen. Supervision: Youzhi Tang. Writing – original draft: Dingyi Shen. Writing – review and editing: Dingyi Shen, Wei Yang, and Youzhi Tang.

## Conflict of Interest

The authors declare no conflict of interest.

## Supporting information



Supporting Information

## Data Availability

Data sharing is not applicable to this article as no new data were created or analyzed in this study.
