# Supplementary material for: Anti‐Inflammatory Mechanisms of Selenium Nanosheets in Ulcerative Colitis: Protein Corona, GP130 Interaction, and Transcriptomic Profile
Source: Adv Sci (Weinh). 2025 Jun 29;12(36):e01832. doi: 10.1002/advs.202501832 (PMC12462919; doi:10.1002/advs.202501832)
Supplement: Supplementary file 1 — Supporting Information [file ADVS-12-e01832-s001.docx]

Supporting Information

**Anti-Inflammatory Mechanisms of Selenium Nanosheets in Ulcerative Colitis: Protein Corona, GP130 Interaction, and Transcriptomic Profile**

Dingyi Shen^1^, Li Gong^2^, Wei Yang^1^, Jiaqi Luo,^1^ Zhen Jin^1^, Youzhi Tang^1^*

**
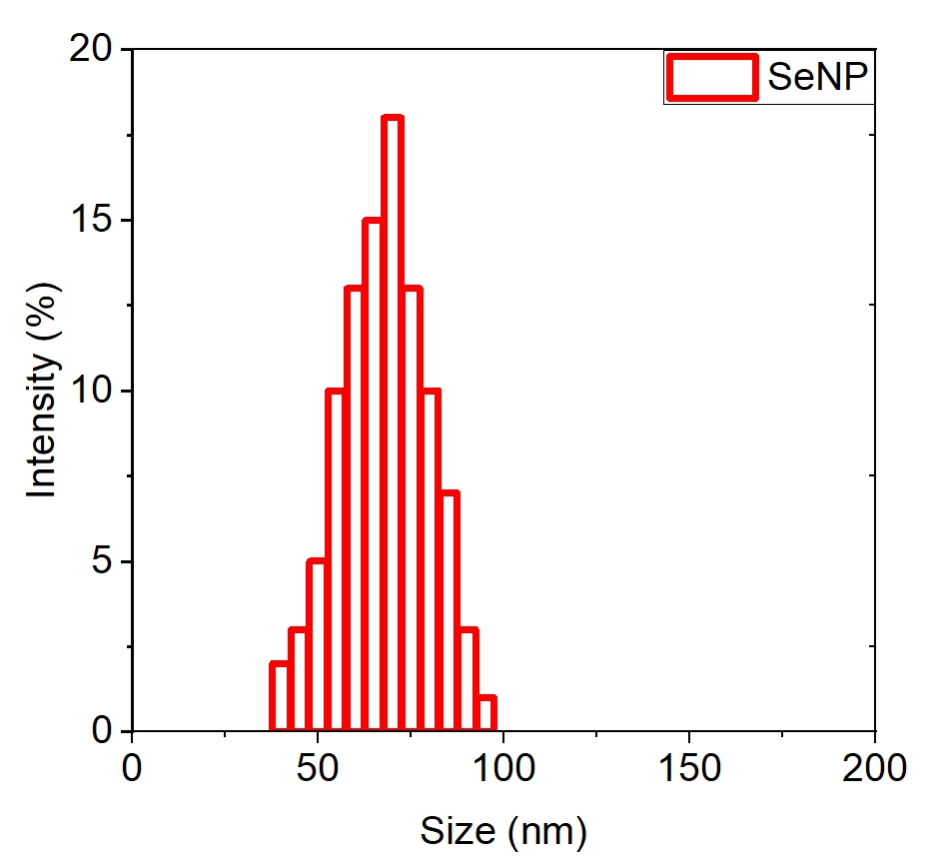
**

**Figure S1.** DLS data for SeNPs. (n = 3 independent experiments).


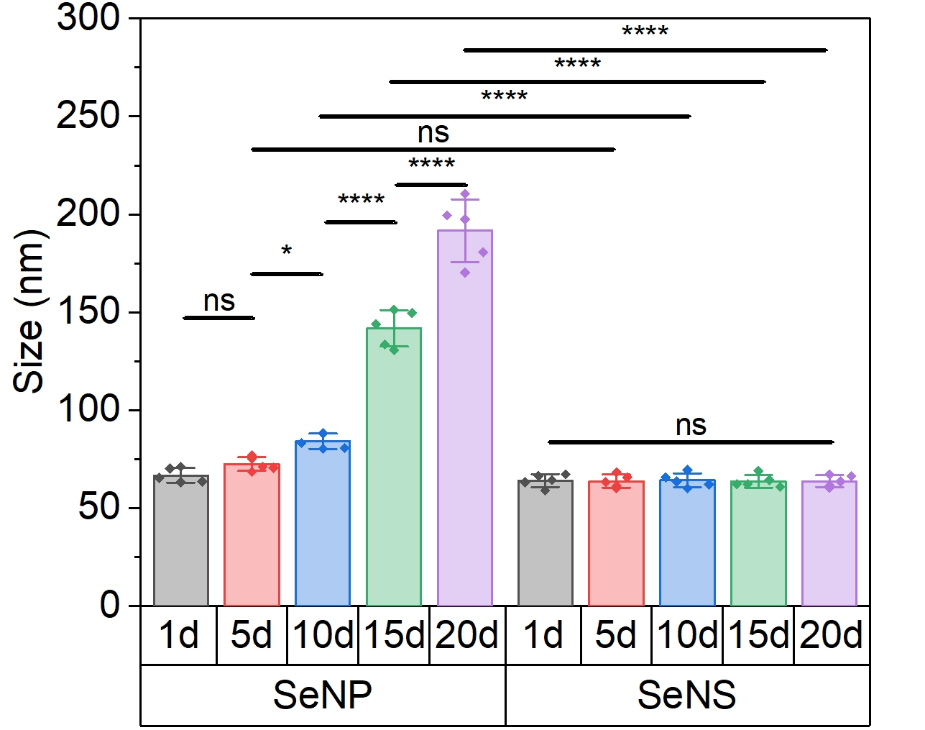


**Figure S2.** Particle size of SeNP and SeNS at different settling days. (**P* < 0.05, ***P* < 0.01, ****P* < 0.001, *****P* < 0.0001; data are expressed as mean ± standard deviation, n = 5 independent experiments, One–way ANOVA).


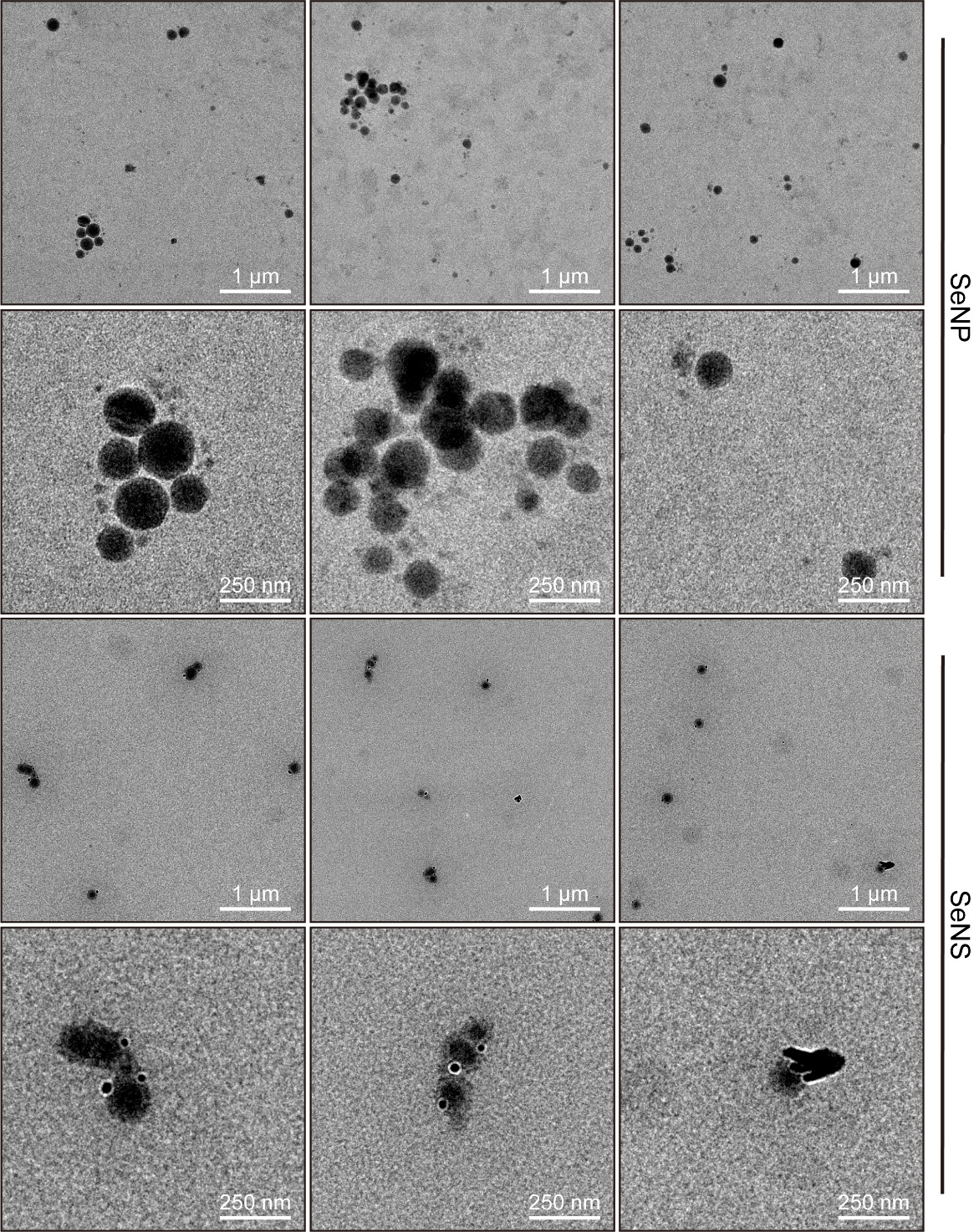


**Figure S3.** TEM images of SeNPs and SeNSs in gastrointestinal fluid for 7 days. (n = 3 independent experiments)


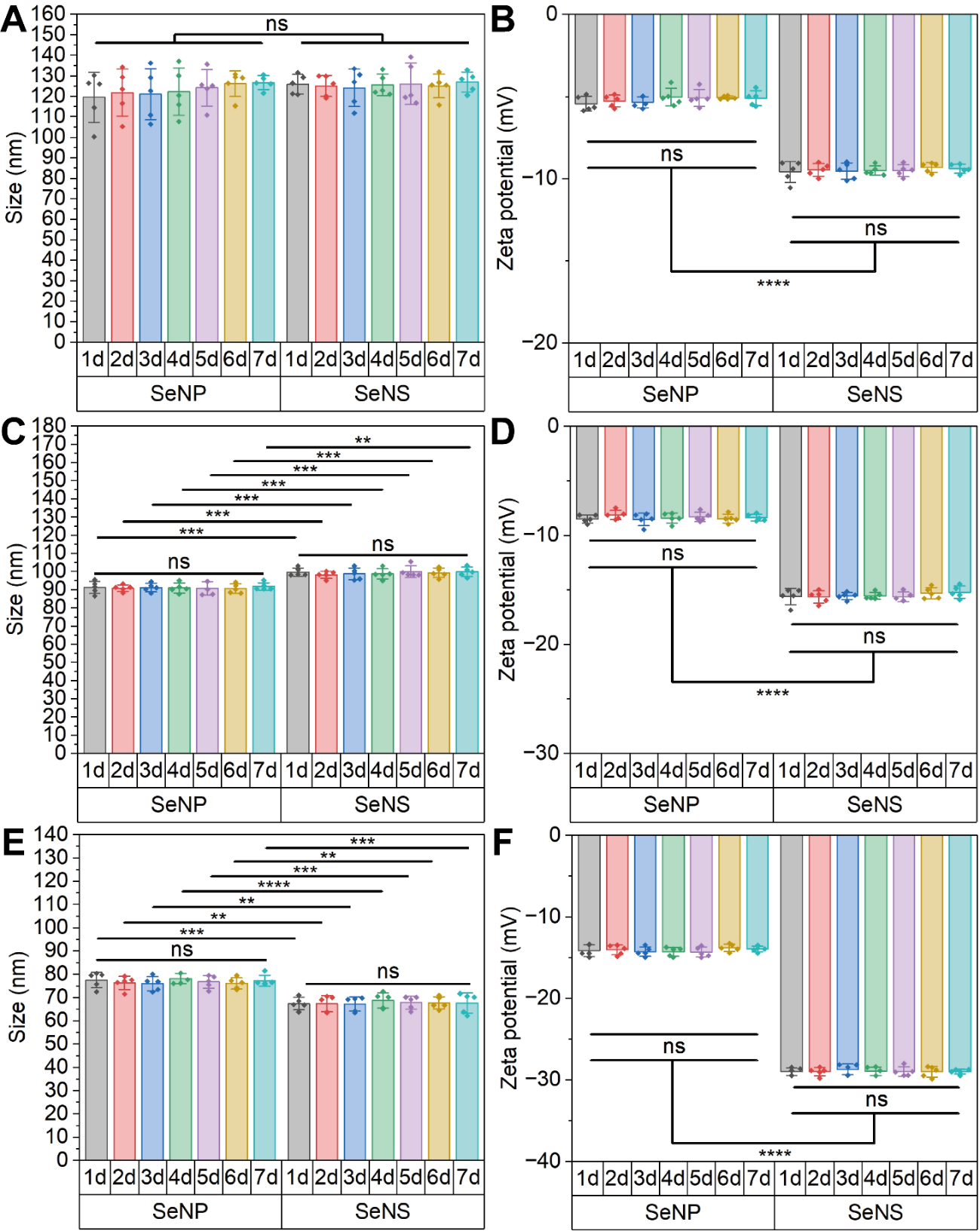


**Figure S4.** Particle sizes and zeta potential of SeNSs and SeNPs in gastric juice. A-B: Particle sizes (A) and zeta potential (B) SeNS and SeNP in simulated gastric juice at pH 1.2. C-D: Particle sizes (C) and zeta potential (D) SeNS and SeNP in simulated gastric juice at pH 3.0. E-F: Particle sizes (E) and zeta potential (F) SeNS and SeNP in simulated gastric juice at pH 6.8. (**P* < 0.05, ***P* < 0.01, ****P* < 0.001, *****P* < 0.0001; data are expressed as mean ± standard deviation, n = 5 independent experiments, One–way ANOVA).


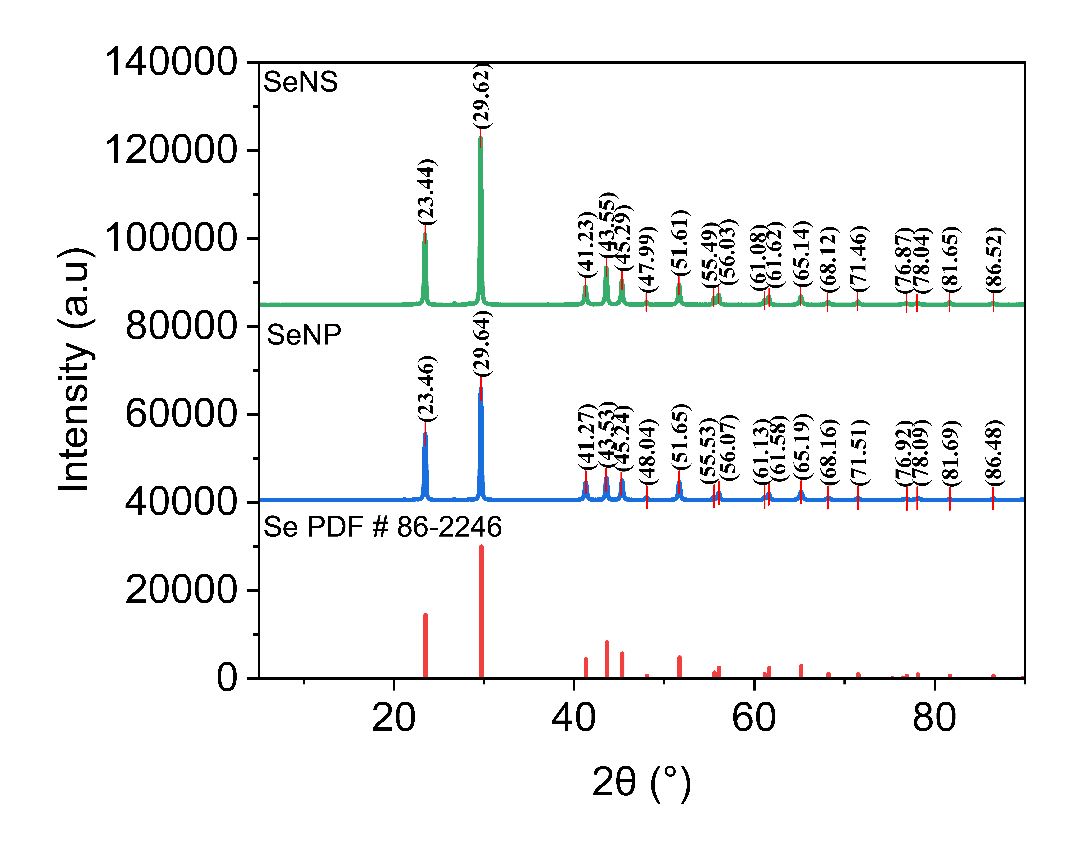


**Figure S5.** XRD patterns of the SeNSs and SeNPs.


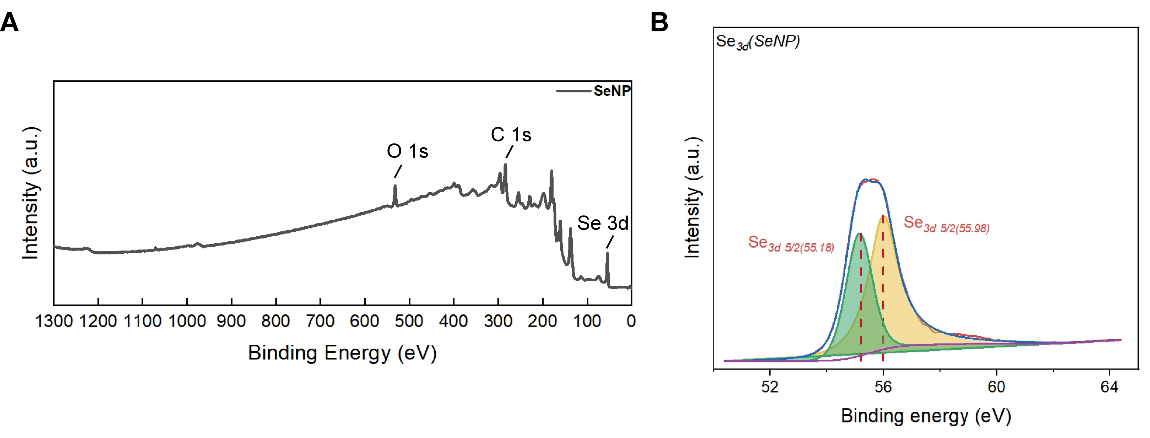
**Figure S6.** XPS spectra of SeNSs (A) and Se*_3d_* spectra of SeNSs (B).


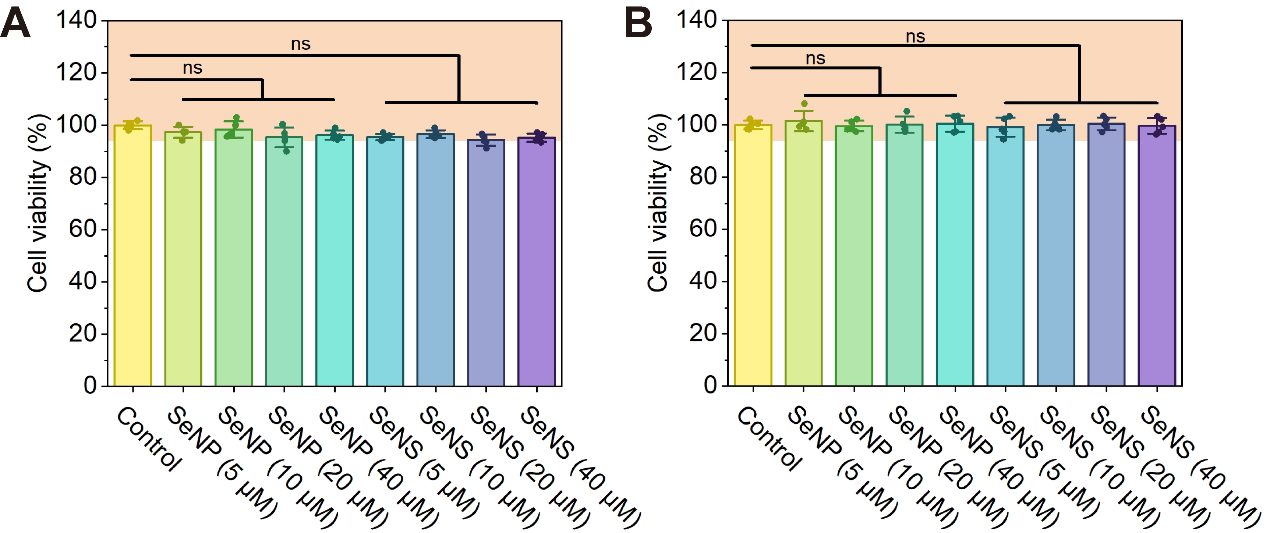


**Figure S7.** Cytotoxicity assay of the SeNSs and SeNPs in Caco-2 (A) and HT29 (B) cells. (**P* < 0.05, ***P* < 0.01, ****P* < 0.001, *****P* < 0.0001; data are expressed as mean ± standard deviation, n = 5 independent experiments, One–way ANOVA).


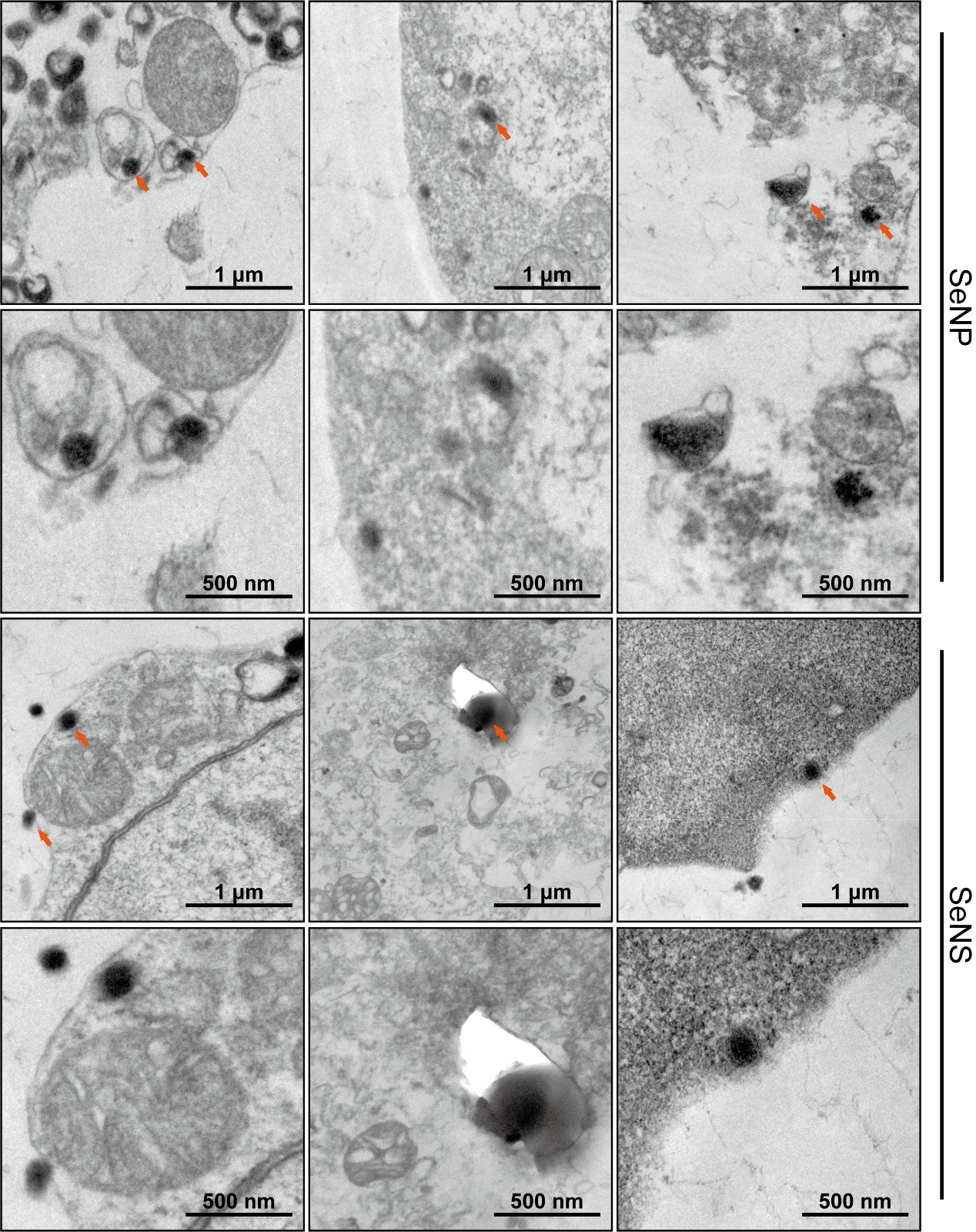


**Figure S8.** TEM images of SeNSs and SeNPs in RAW264.7. (n = 3 independent experiments).

**
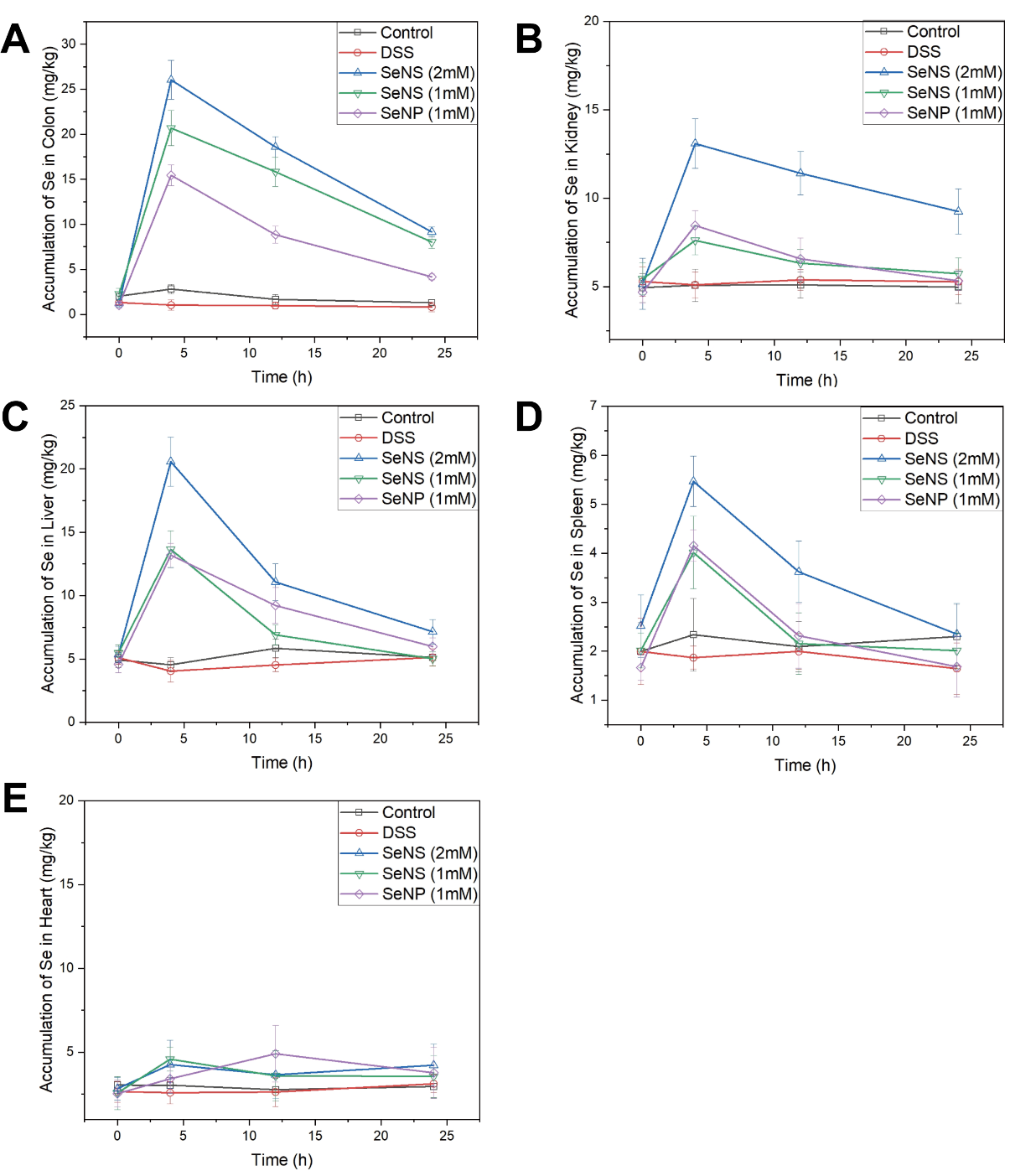
**

**Figure S9.** Accumulation of selenium in various tissues: A: colon; B: kidney; C: liver; D: spleen; and E: heart. (data are expressed as mean ± standard deviation, n = 5 independent experiments, One–way ANOVA).

**
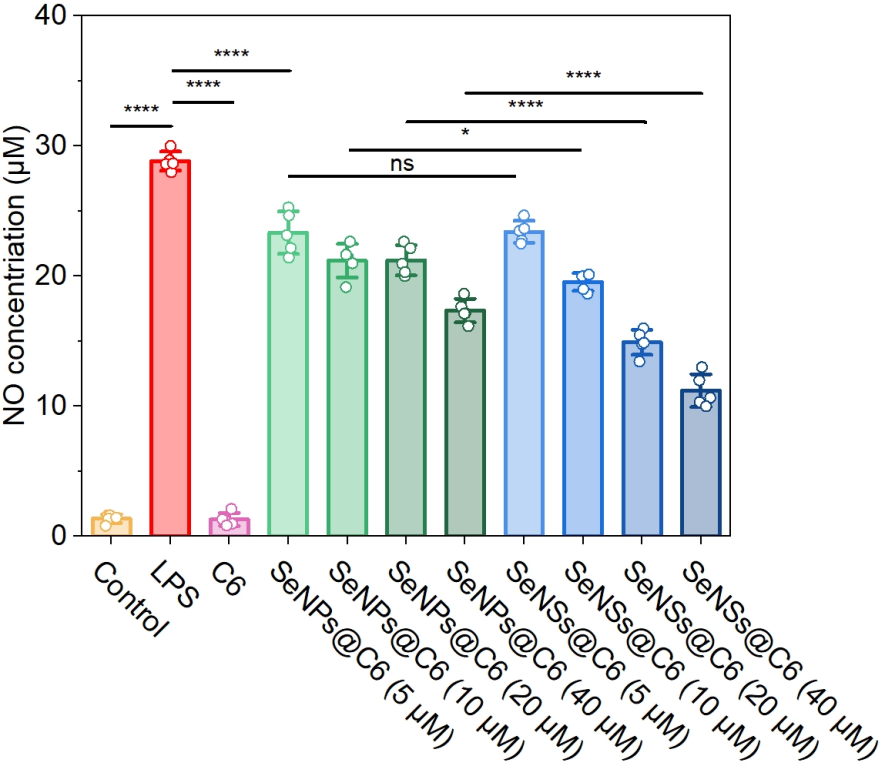
**

**Figure S10.** NO concentration under different treatments. (**P* < 0.05, ***P* < 0.01, ****P* < 0.001, *****P* < 0.0001; data are expressed as mean ± standard deviation, n = 5 independent experiments, One–way ANOVA).

**
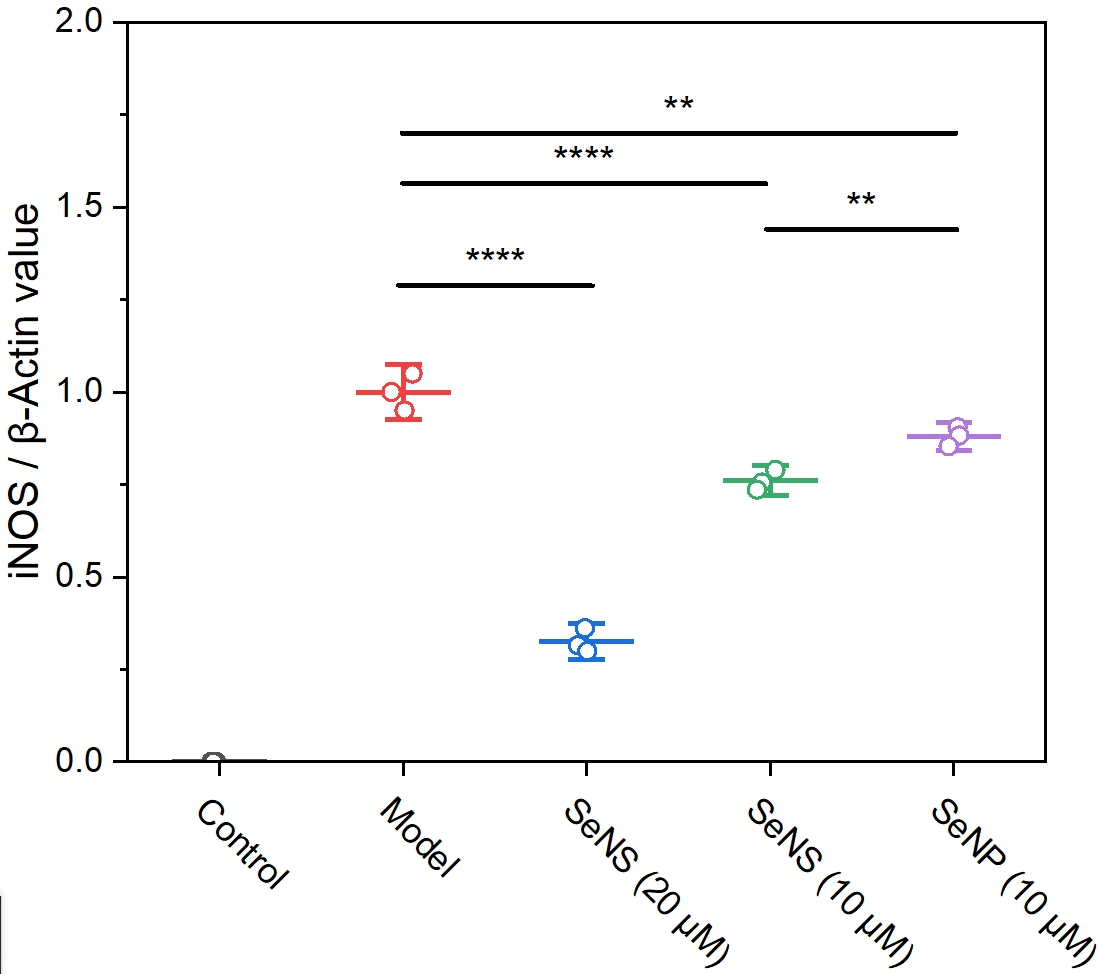
**

|  | Control | LPS | SeNS  (20 μM) | SeNS  (10 μM) | SeNP  (10 μM) |
| --- | --- | --- | --- | --- | --- |
| #1 | 0.00 | 1.07 | 0.36 | 0.78 | 0.90 |
| #2 | 0.00 | 1.00 | 0.32 | 0.75 | 0.88 |
| #3 | 0.00 | 0.93 | 0.29 | 0.74 | 0.85 |

**Figure S11.** Quantification of the Western blot results and protein data for iNOS in RAW264.7 cells. (**P* < 0.05, ***P* < 0.01, ****P* < 0.001, *****P* < 0.0001; data are expressed as mean ± standard deviation, n = 3 independent experiments, One–way ANOVA).


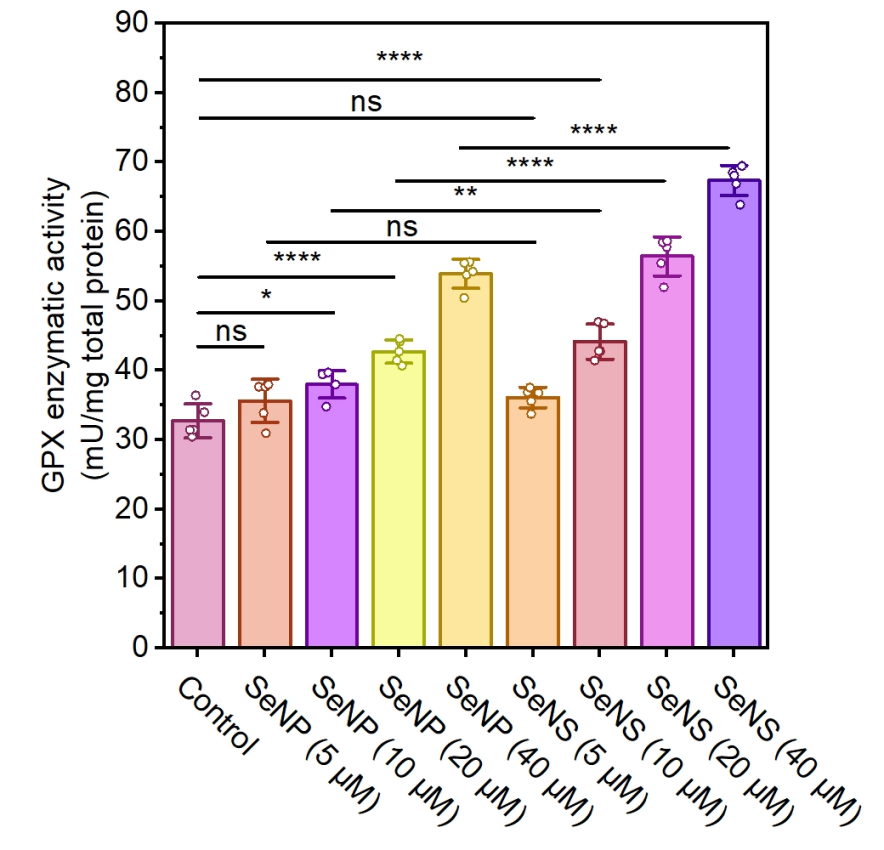


**Figure S12.** Effect of different nano-selenium treatments on the activity of GPX in RAW264.7 cells. (**P* < 0.05, ***P* < 0.01, ****P* < 0.001, *****P* < 0.0001; data are expressed as mean ± standard deviation, n = 5 independent experiments, One–way ANOVA).


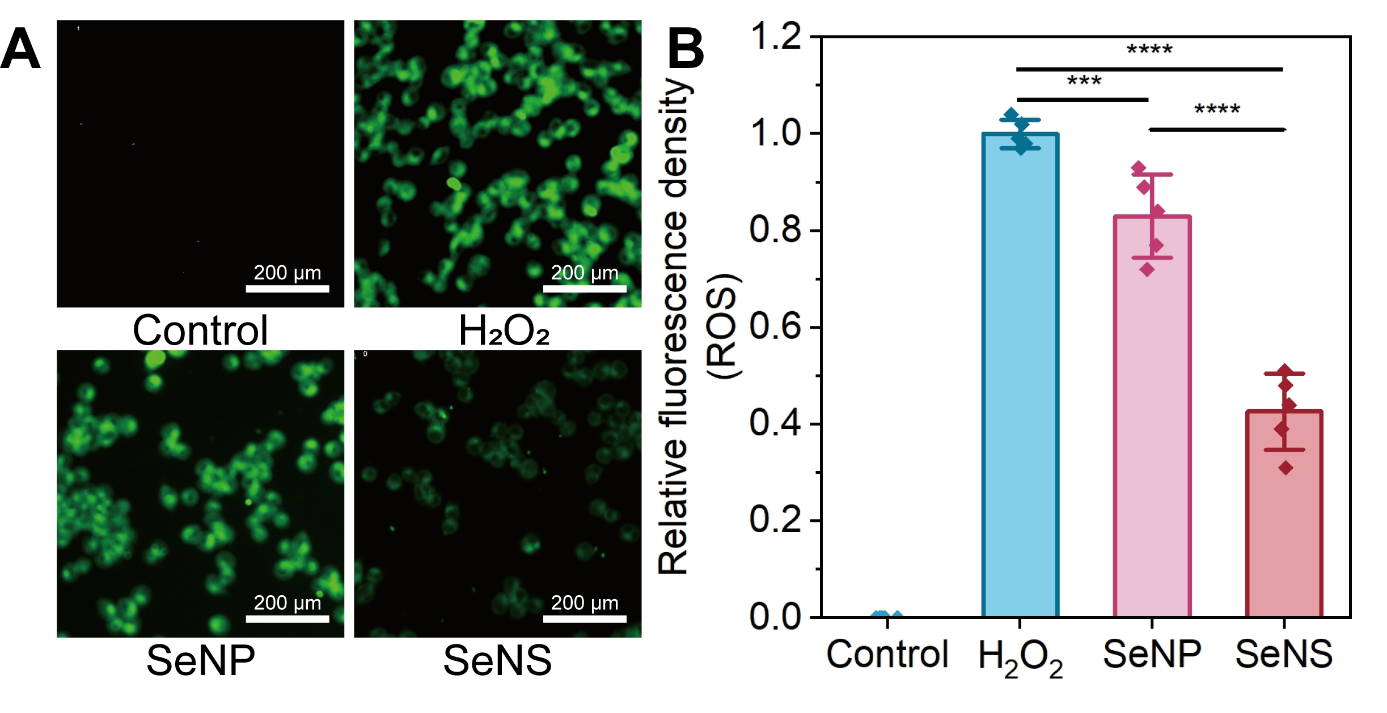


**Figure S13.** Intracellular ROS level evaluated by DCF-DA assay. A: The fluorescence inverted microscope images of ROS scavenging by SeNS or SeNP. B: Fluorescence intensity of ROS in RAW264.7 cells. (**P* < 0.05, ***P* < 0.01, ****P* < 0.001, *****P* < 0.0001; data are expressed as mean ± standard deviation, n = 5 independent experiments, One–way ANOVA).

**
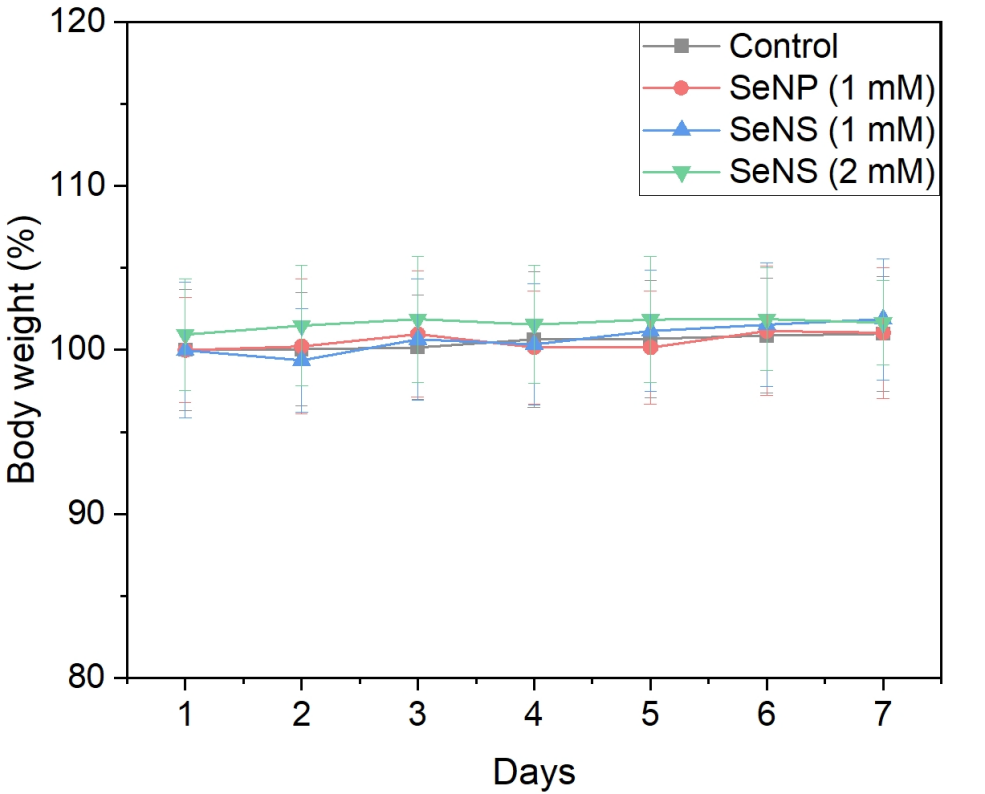
**

**Figure S14.** Body weight changes in mice subjected to different treatments. (Data are expressed as mean ± standard deviation, n = 5 independent experiments, One–way ANOVA).


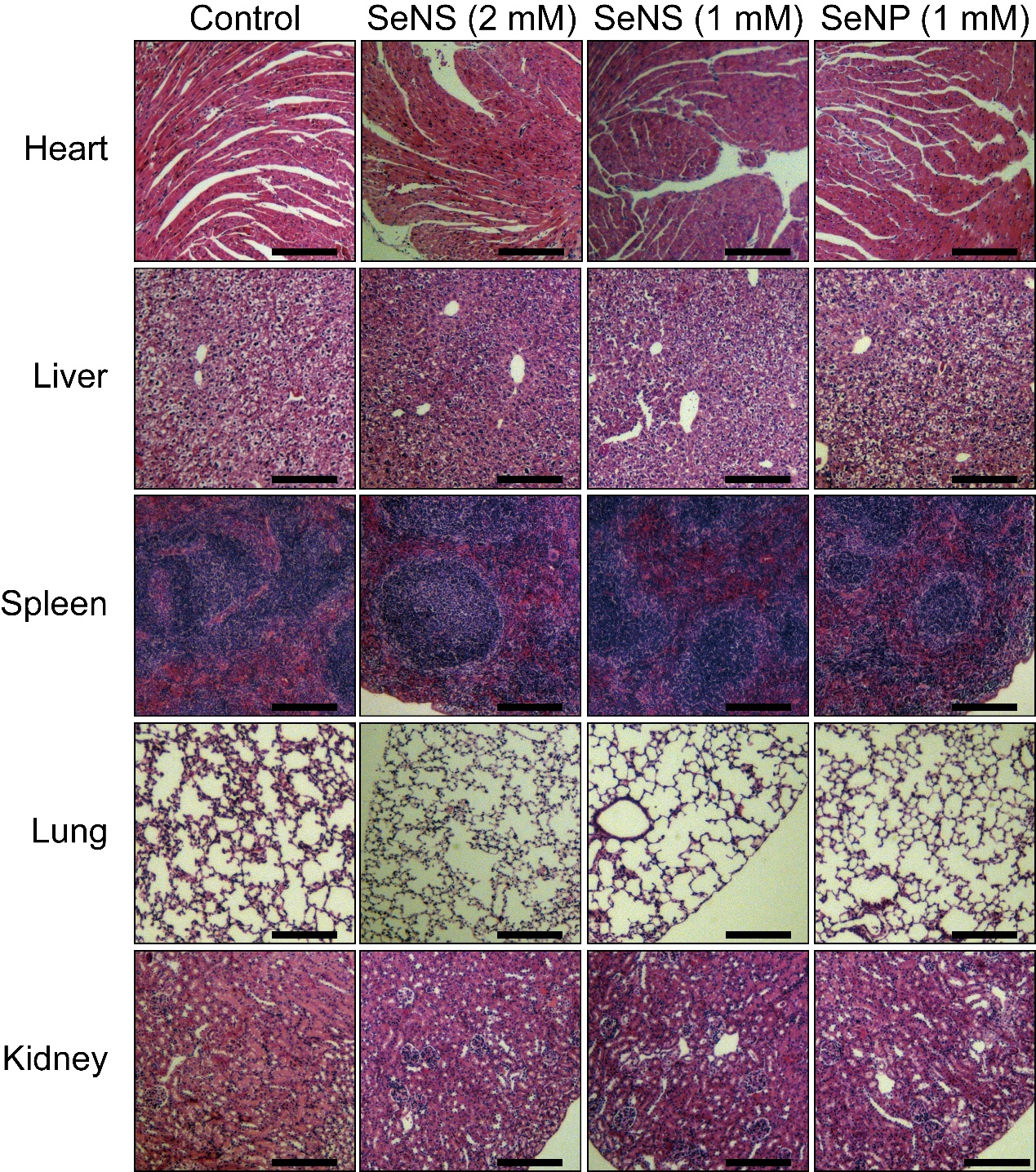


**Figure S15.** Representative H&E-stained histological sections of the main organs after treatment. Scale bar: 200 μm. (n = 5 independent experiments).


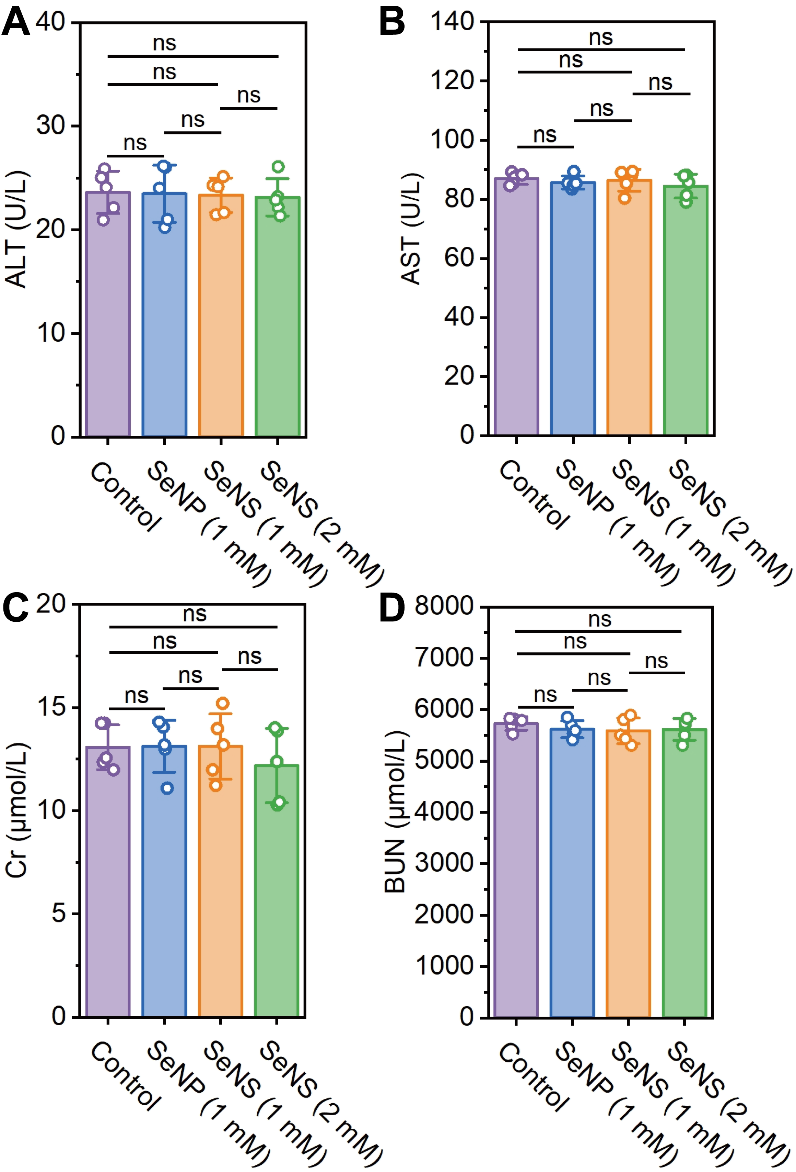


**Figure S16.** Effect of SeNSs and SeNPs on the activity of alanine aminotransferase (ALT) and aspartate aminotransferase (AST) and the content of creatinine (Cr) and blood urea nitrogen (BUN). A: ALT; B: AST; C: Cr; D: BUN. (**P* < 0.05, ***P* < 0.01, ****P* < 0.001, *****P* < 0.0001; data are expressed as mean ± standard deviation, n = 5 independent experiments, One–way ANOVA).

**
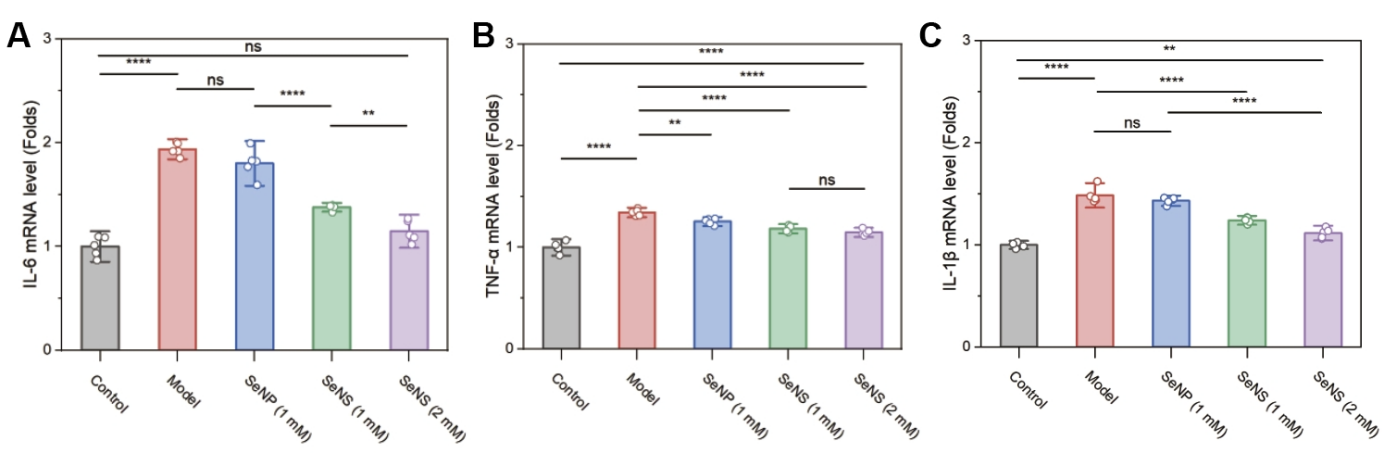
Figure S17.** The mRNA expression levels of cytokines in colon tissue: A: IL-6; B: TNF-α; and C: IL-1β. (**P* < 0.05, ***P* < 0.01, ****P* < 0.001, *****P* < 0.0001; data are expressed as mean ± standard deviation, n = 5 independent experiments, One–way ANOVA).


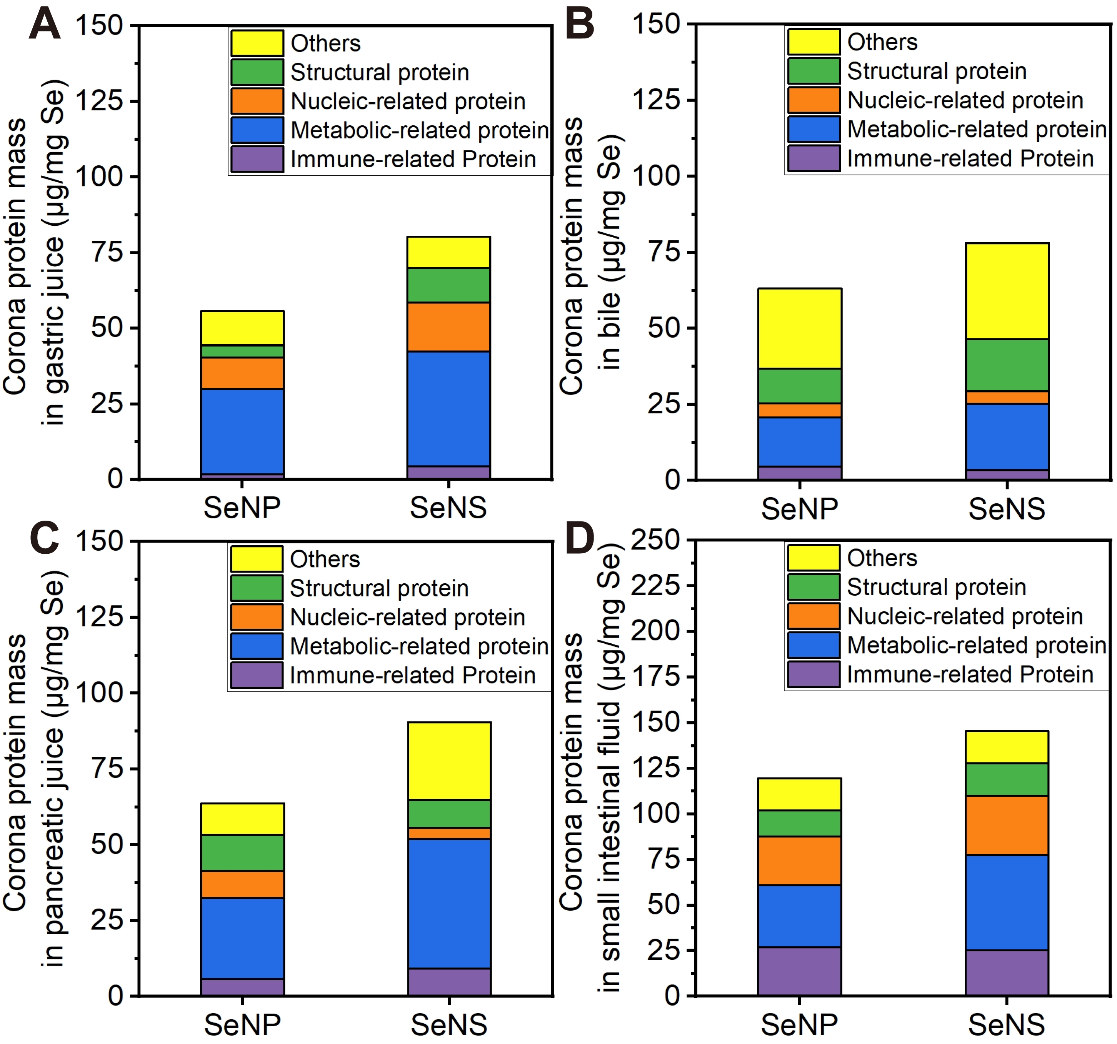


**Figure S18.** The protein corona composition of SeNS and SeNP in gastric juice (A), bile (B), pancreatic juice (C), and small intestinal fluid (D). (n = 3 independent experiments).

**
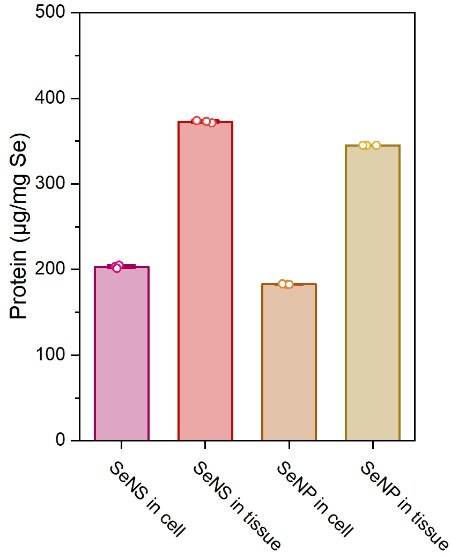
Figure S19.** Quantification of proteins adsorbed on nanovesicles isolated from RAW264.7 cells and colon tissues. (n = 3 independent experiment).


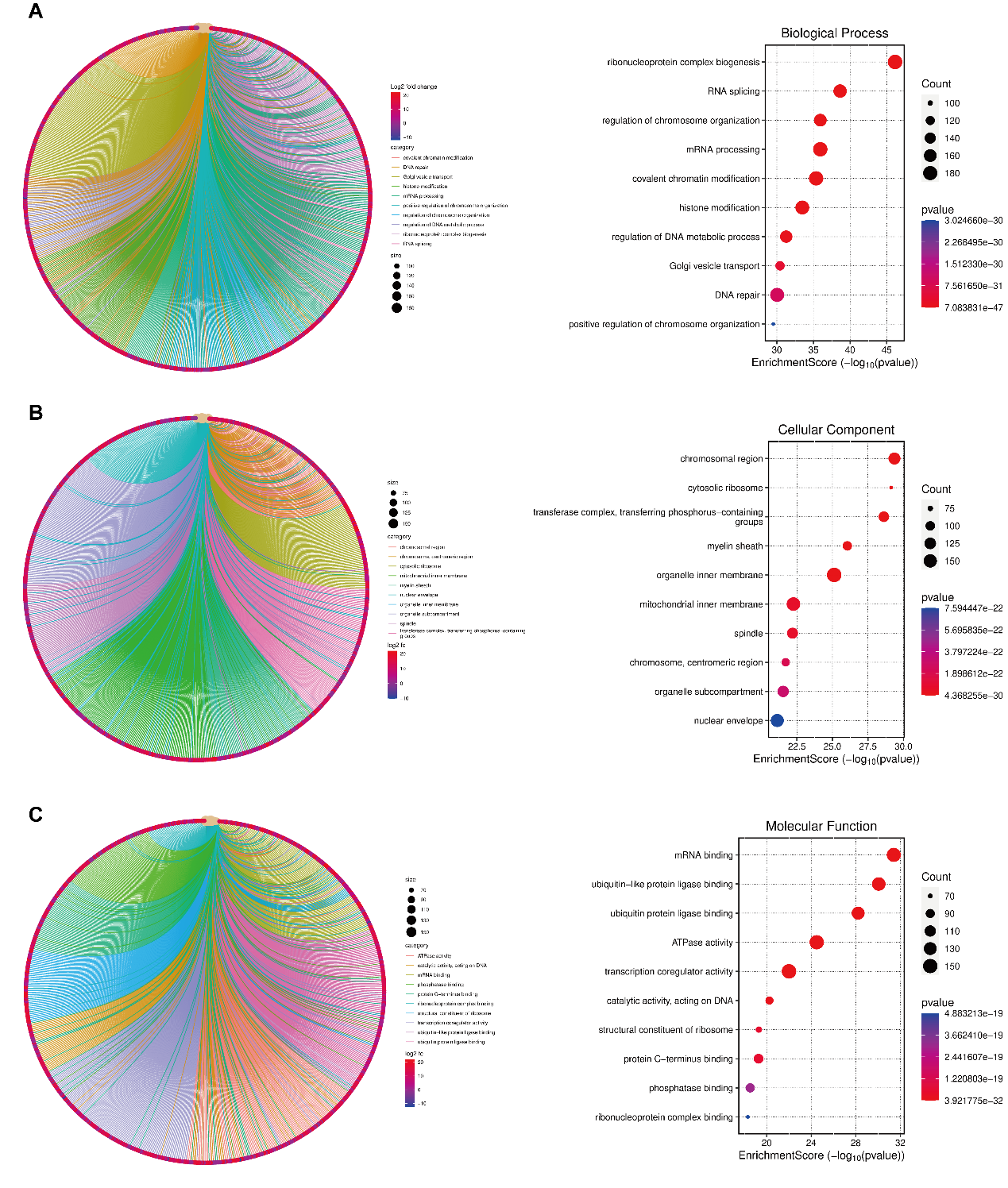


**Figure S20.** GO pathway enrichment analysis of differentially expressed proteins in the SeNS protein corona compared to the SeNP protein corona in cells. A: Biological process; B: Cellular component; and C: Molecular function. (n = 3 independent experiments).


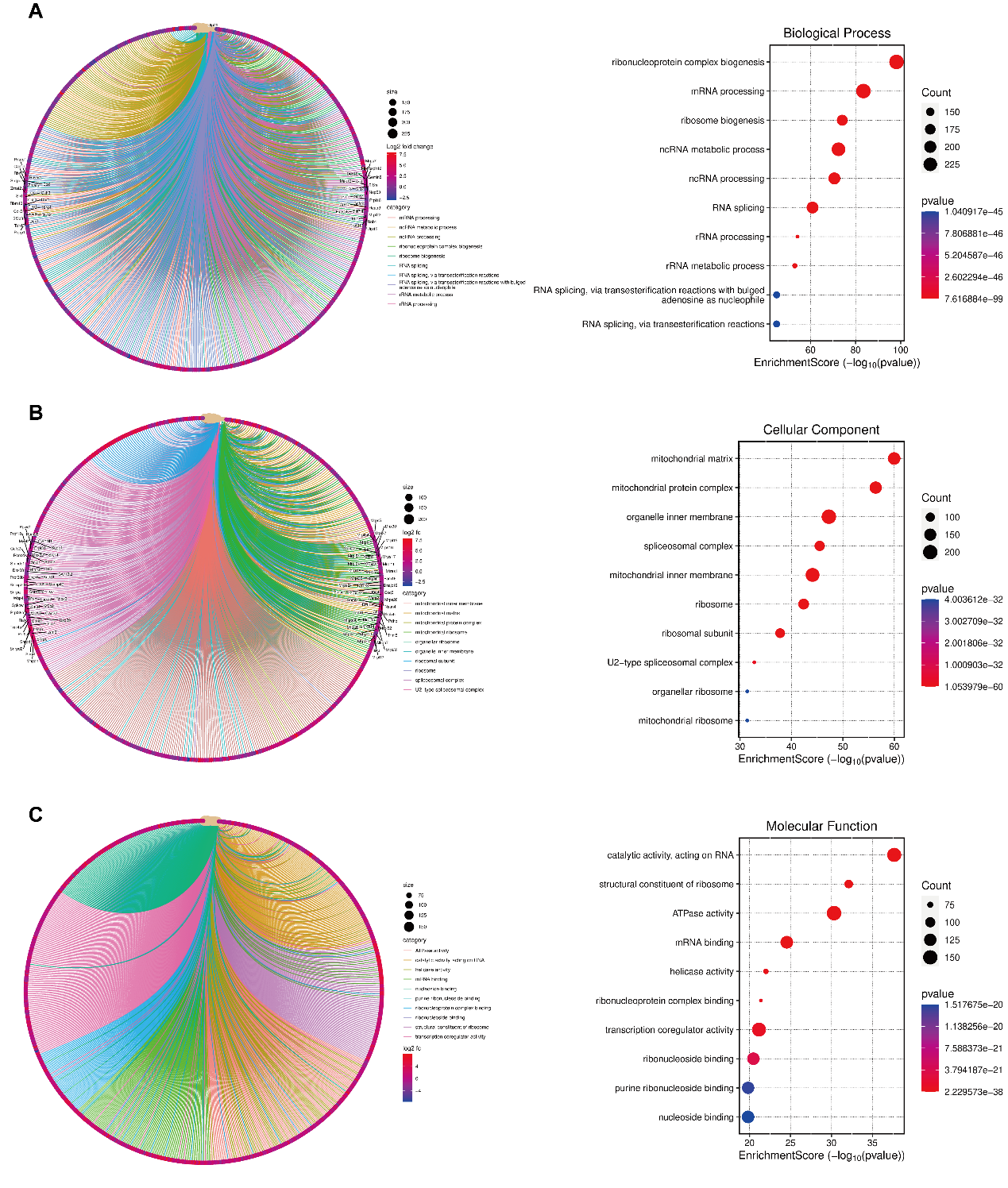


**Figure S21.** GO pathway enrichment analysis of differentially expressed proteins in the SeNS protein corona compared to the SeNP protein corona in colon tissues. A: Biological process; B: Cellular component; and C: Molecular function. (n = 3 independent experiments).


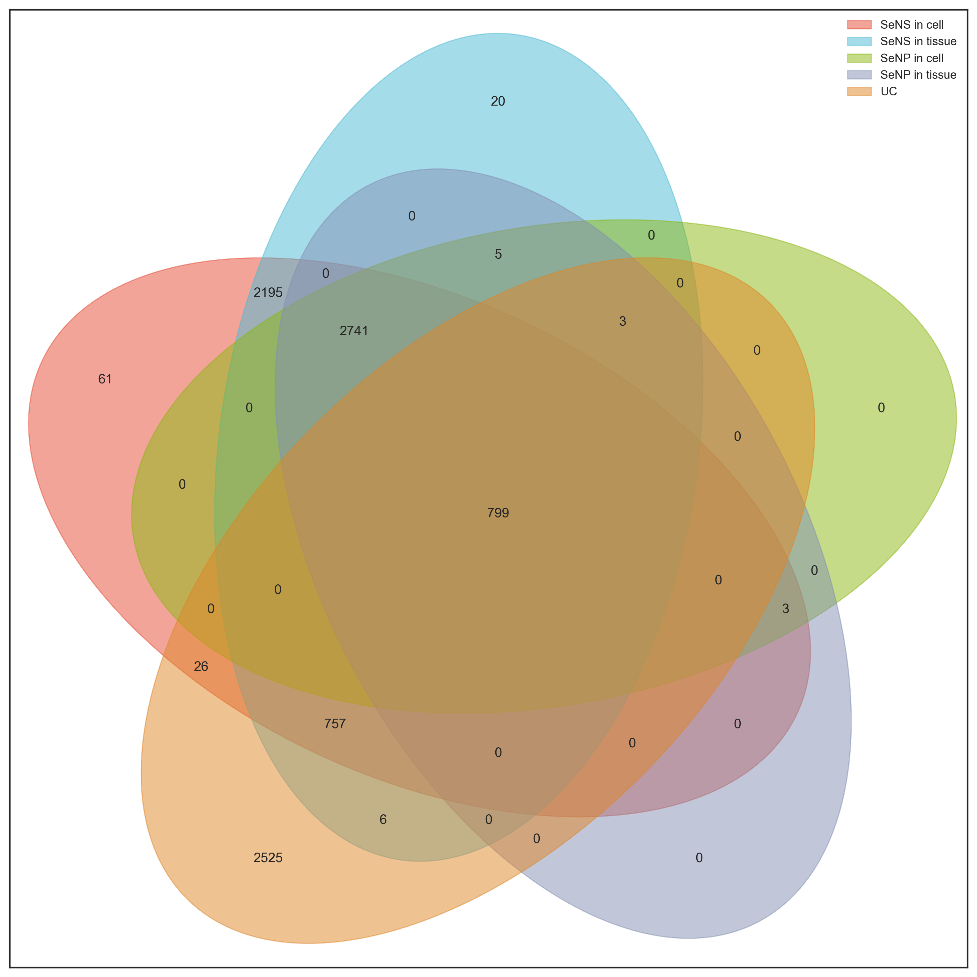


**Figure S22.** Venn diagram showing shared proteins in the SeNS and SeNP protein coronas within cells or tissues. (n = 3 independent experiments).

**
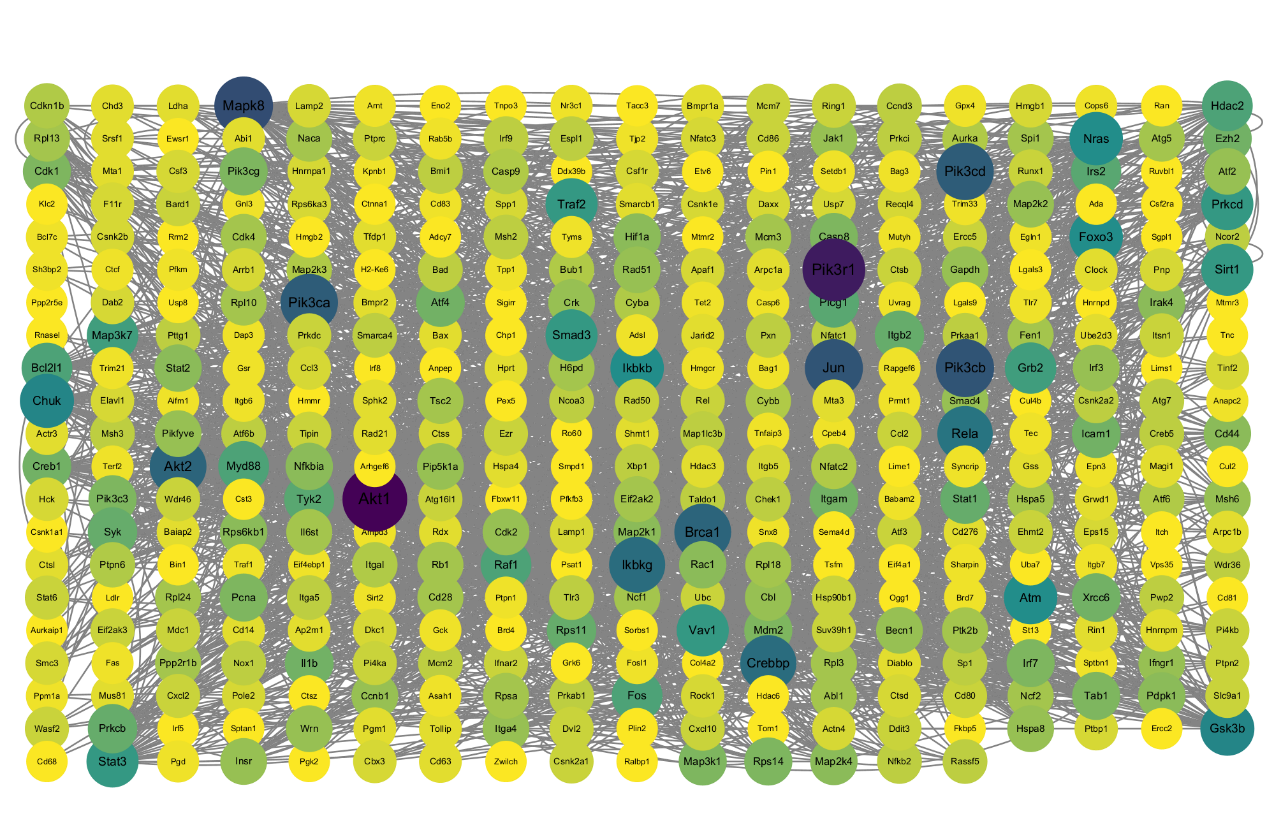
Figure S23.** PPI network of UC-related proteins identified exclusively in the protein corona of SeNSs in cells but absent in SeNPs. (n = 3 independent experiments).

**
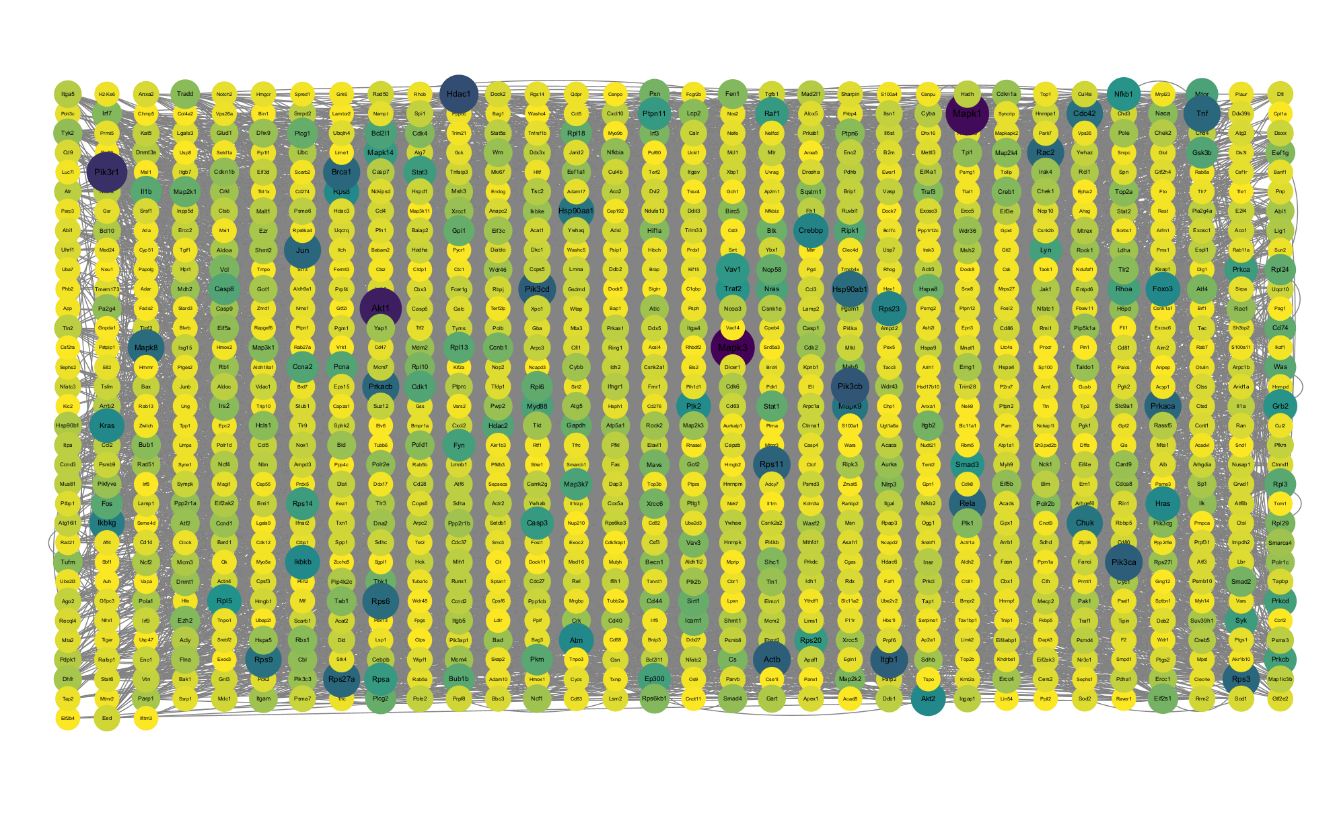
Figure S24.** PPI network of UC-related proteins identified in the protein corona of SeNSs in cells. (n = 3 independent experiments).

**
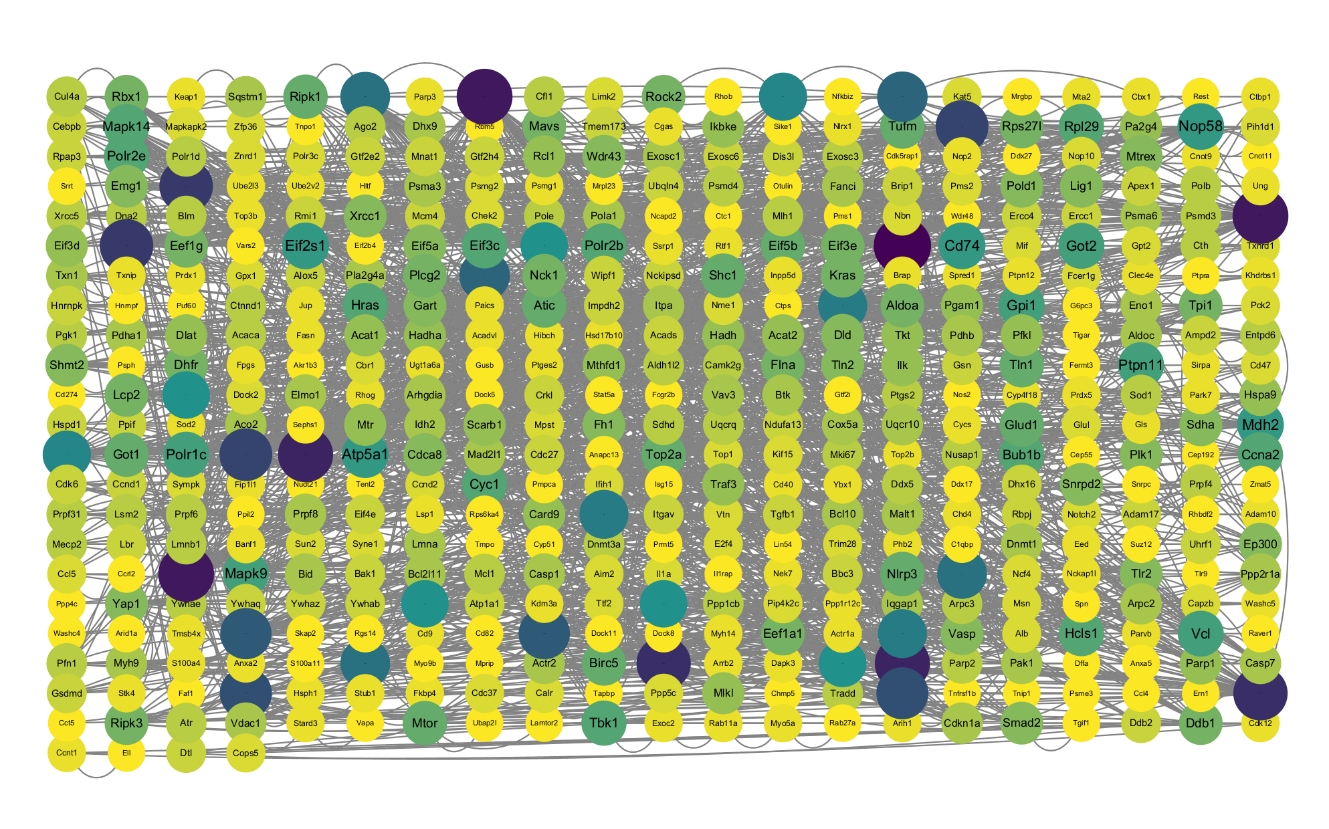
Figure S25.** PPI network of UC-related proteins identified in the protein corona of SeNPs in cells. (n = 3 independent experiments).

**
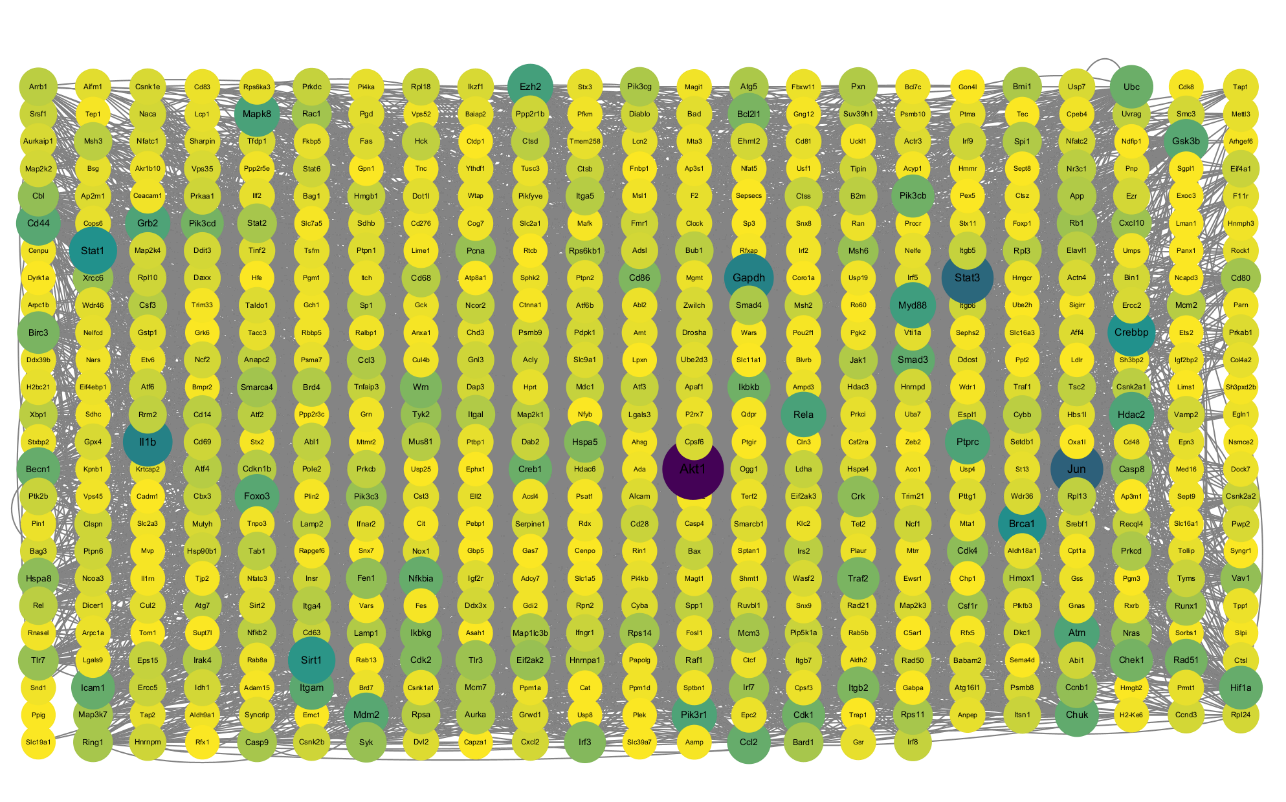
Figure S26.** PPI network of UC-related proteins identified exclusively in the protein corona of SeNSs in tissues but absent in SeNPs. (n = 3 independent experiments).


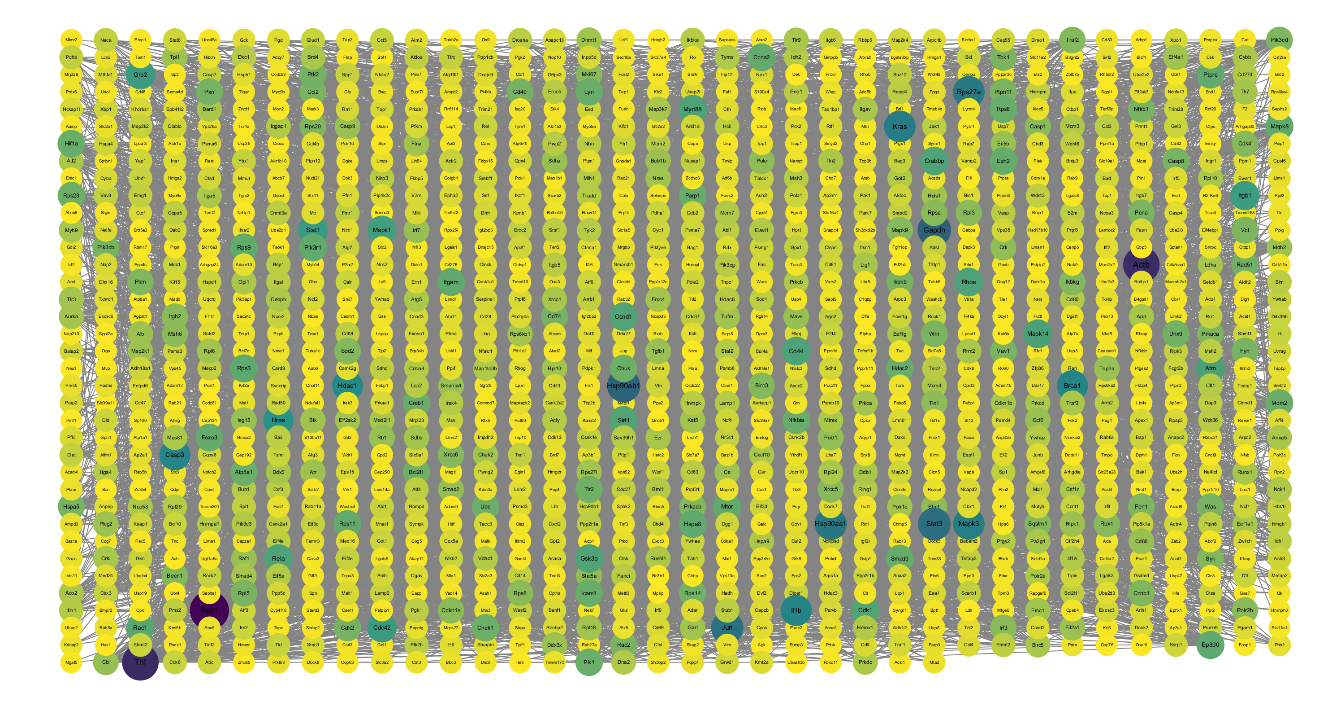
**Figure S27.** PPI network of UC-related proteins identified in the protein corona of SeNSs in tissues. (n = 3 independent experiments).

**
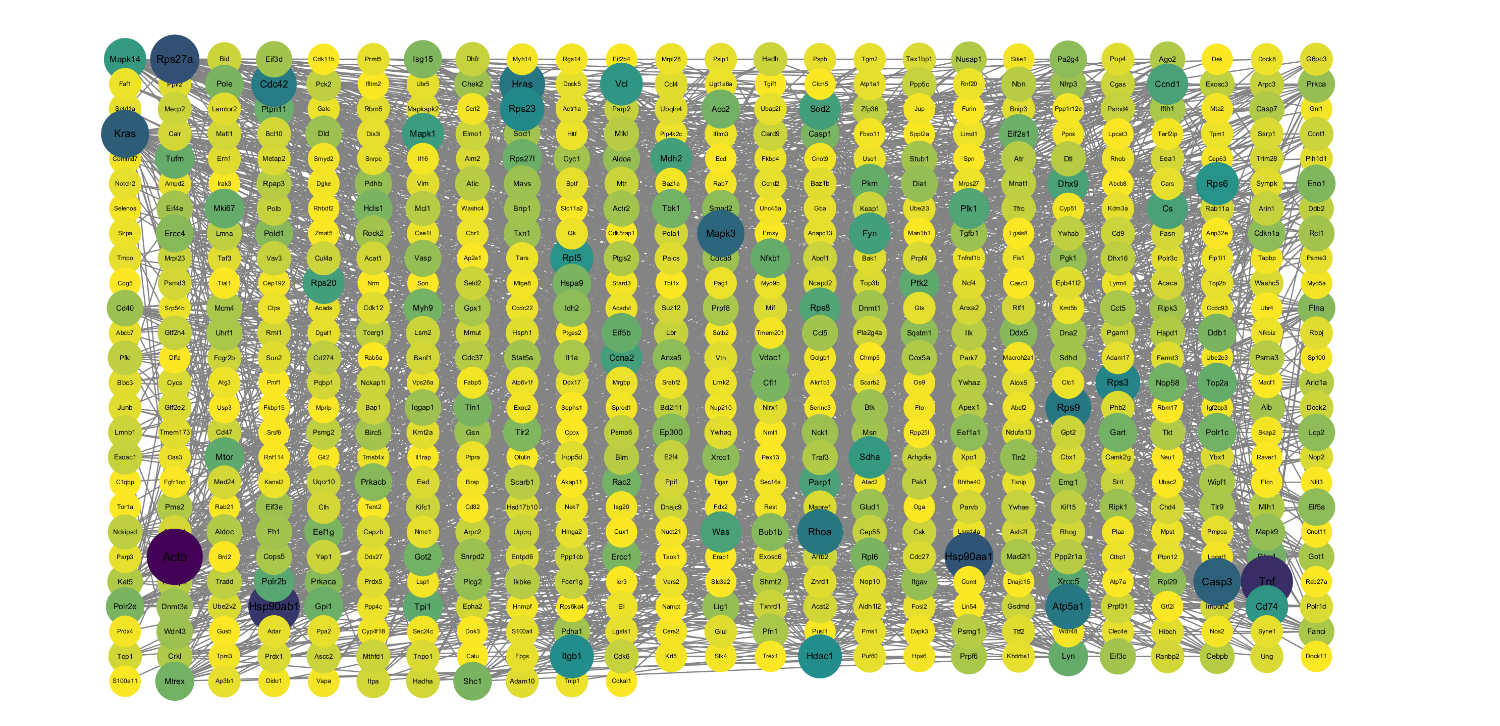
Figure S28.** PPI network of UC-related proteins identified in the protein corona of SeNPs in tissues. (n = 3 independent experiments).


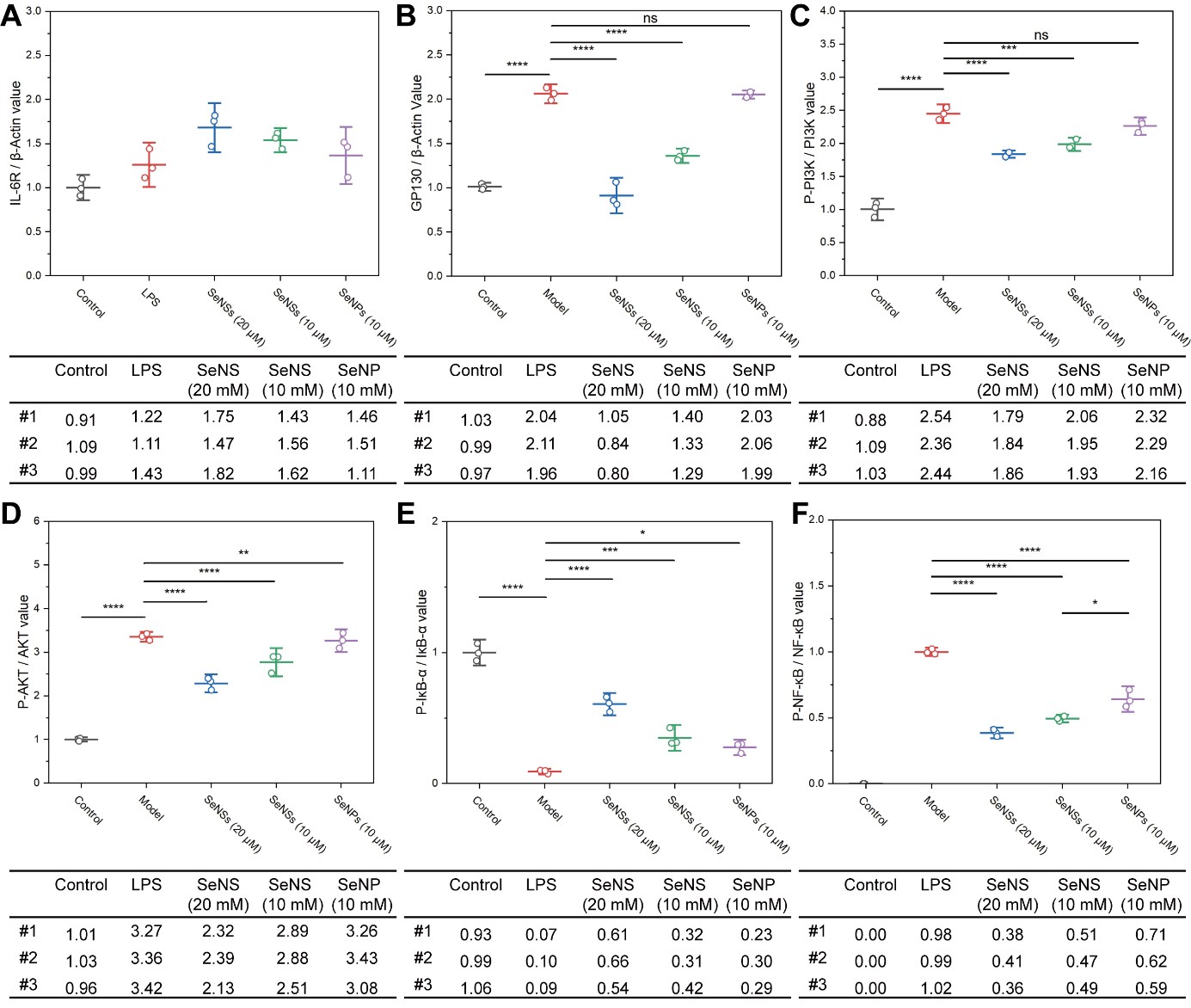
**Figure S29.** Quantitative analysis of Western blot results for the following proteins: A: IL-6R; B: GP130; C: Phosphorylated PI3K/PI3K; D: Phosphorylated AKT/AKT; E: Phosphorylated IκB-α/IκB-α; and F: Phosphorylated NF-κB/NF-κB. (**P* < 0.05, ***P* < 0.01, ****P* < 0.001, *****P* < 0.0001; data are expressed as mean ± standard deviation, n = 3 independent experiments, One–way ANOVA).


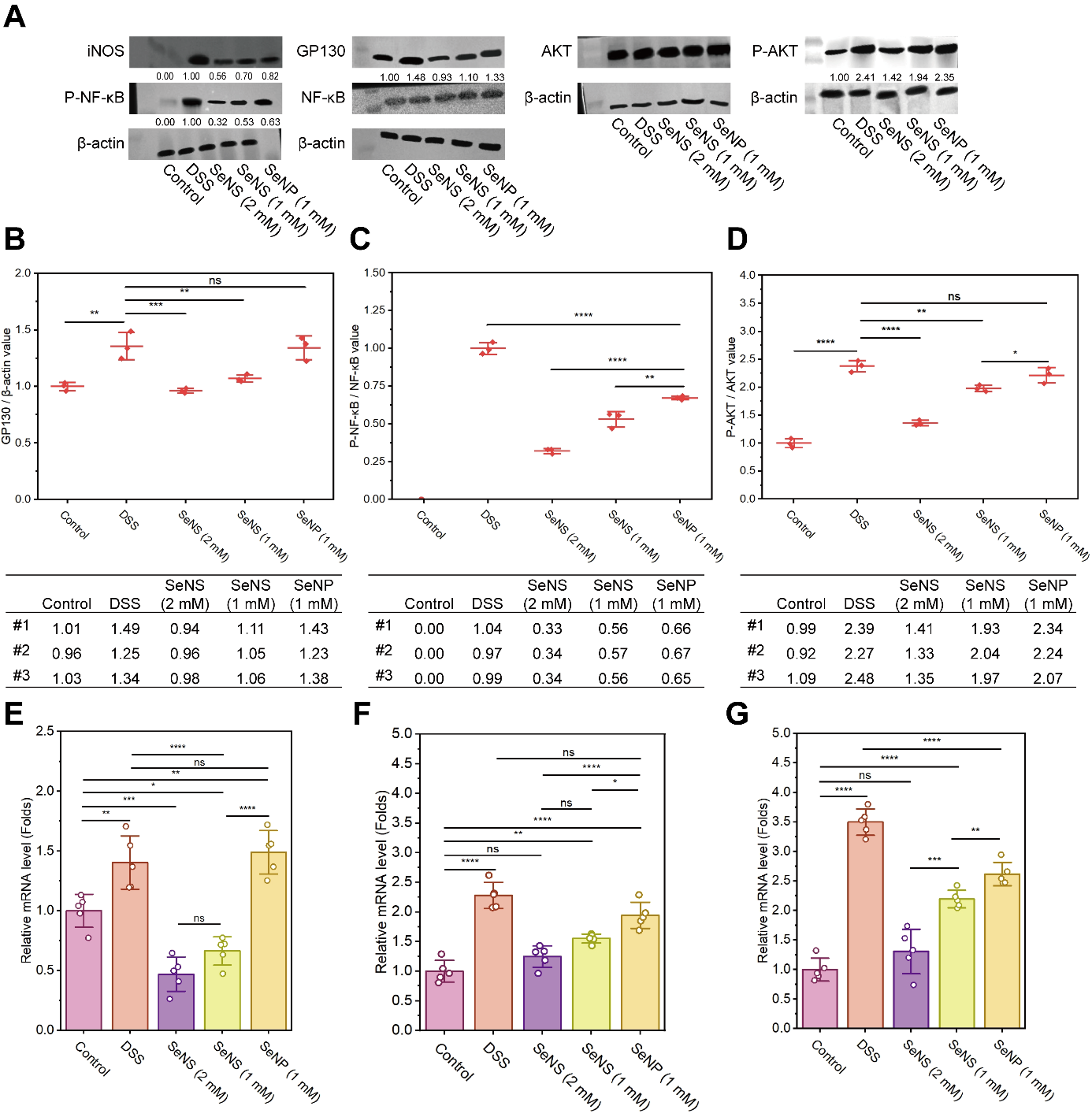


**Figure S30.** Anti-inflammatory mechanism of the SeNSs in tissues. A: Raw western blot analyses of PI3K/AKT and NF-κB signaling pathway components in colon tissues. B-D: Quantification of the Western blot results for GP130 (B), phosphorylated AKT (C), and phosphorylated NF-κB (D). E-G: mRNA expression levels of GP130 (E), AKT (F), and NF-κB (G). (**P* < 0.05, ***P* < 0.01, ****P* < 0.001, *****P* < 0.0001; data are expressed as mean ± standard deviation, n = 3 independent experiments, One–way ANOVA).

** The protein bands are presented in a segmented format because the Western blot results were obtained from different gels (membranes). The p-NF-κB (Figure S30A) and iNOS (Figure 5J) proteins were detected from the same sample batch on the same SDS-PAGE gel. The gel was cut by molecular weight (~65 kDa for p-NF-κB, ~130 kDa for iNOS), and each part was transferred to separate membranes. Both used the same β-actin as the internal control.*


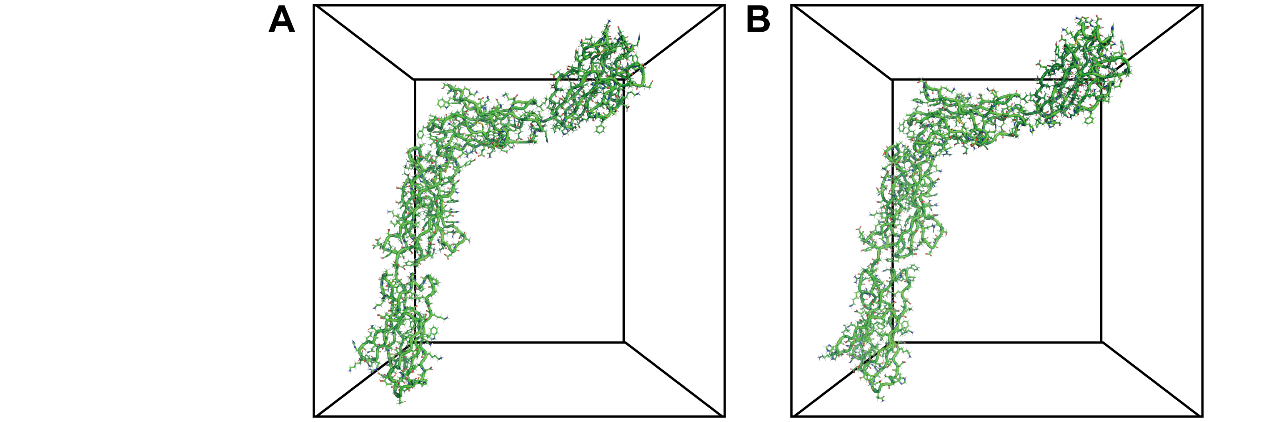
**Figure S31.** Visualized structural changes in GP130 from 0 ps (A) to 1000 ps (B) during molecular dynamics simulations.

**
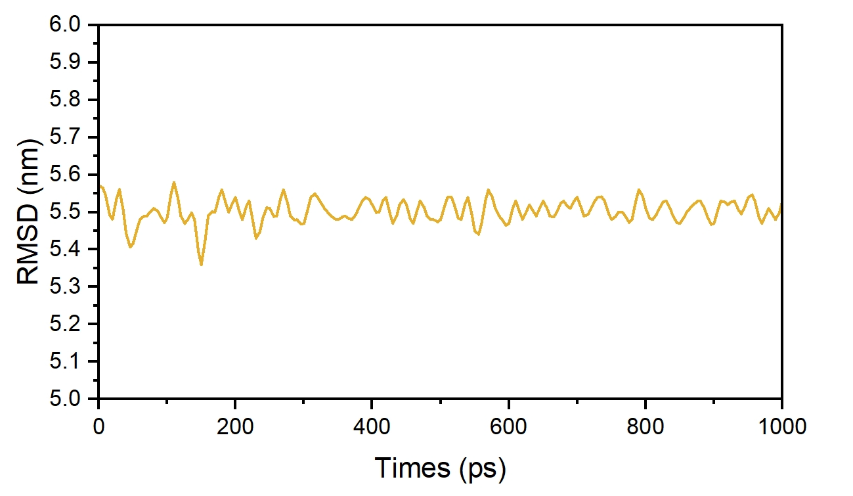
**

**Figure S32.** RMSD values of GP130 as a function of simulation time.


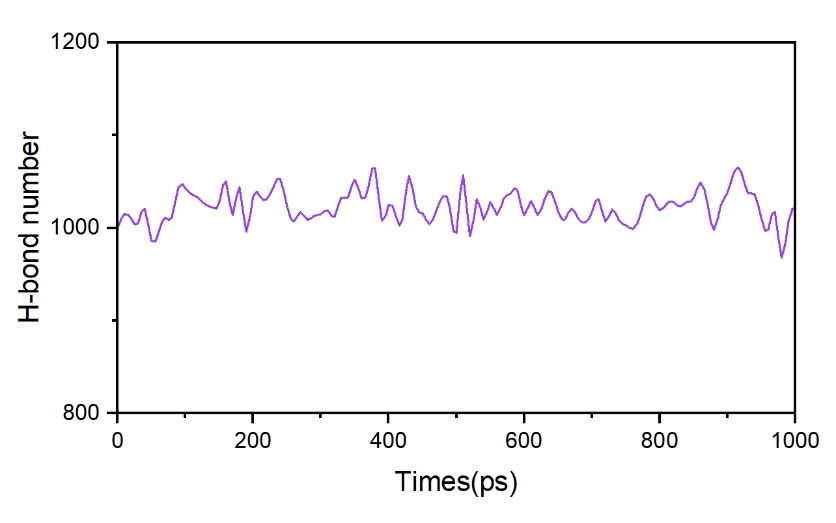


**Figure S33.** Time evolution of hydrogen bond numbers during simulations.


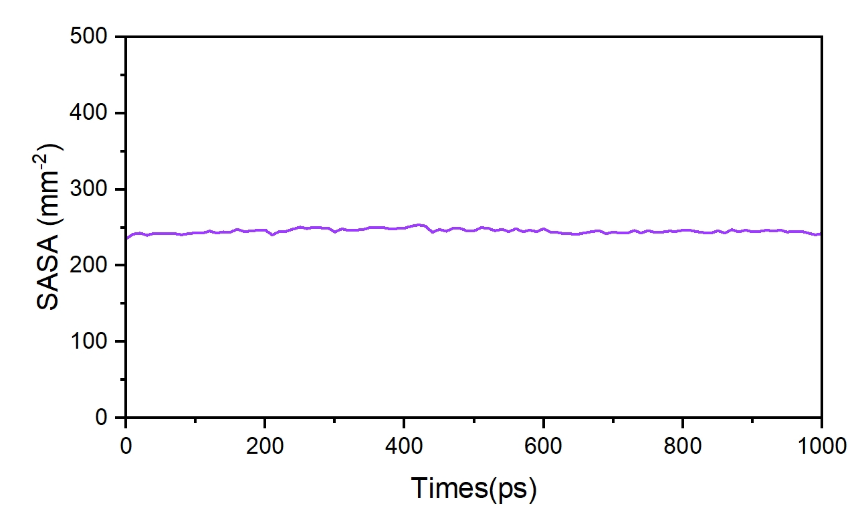
**Figure S34.** Solvent-accessible surface area (SASA) values of GP130 over time.

**
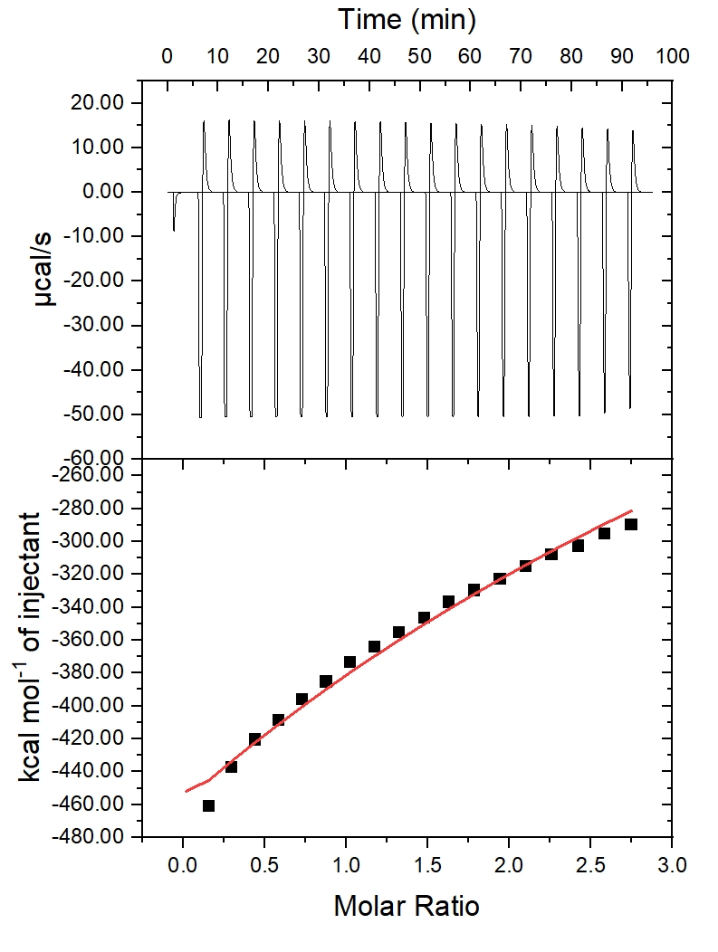
**

**Figure S35.** (A) The binding isotherm generated by plotting the areas under the peaks in (B) against the SeNP-to-GP130 molar ratio.


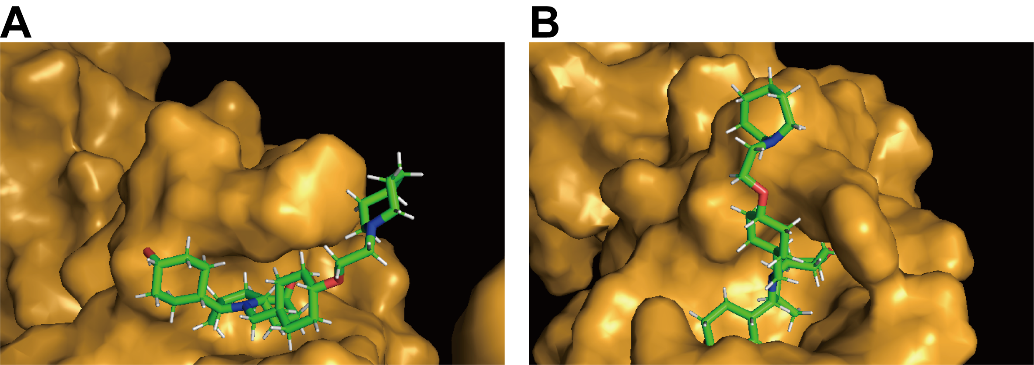


**Figure S36.** Interaction between GP130 and BZA. A-B: Visualized structures of the BZA and GP130 at 0 ps (A) and 10,000 ps (B).


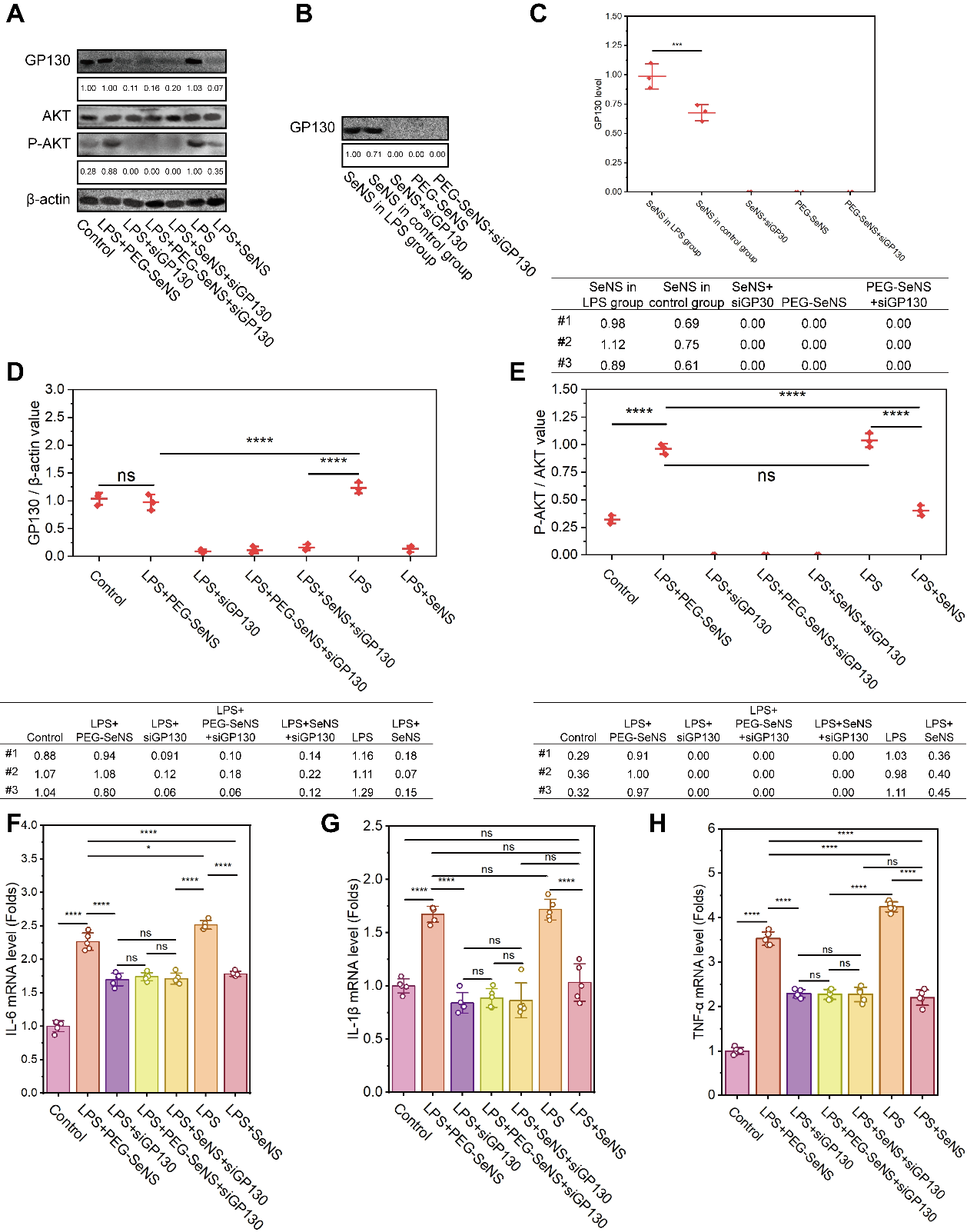


**Figure S37.** Anti-inflammatory mechanism of interaction between GP130 and SeNS. A: Western blot analyses of GP130 and AKT under different treatments. B-C: Western blot analyses (B) and quantification (C) of GP130 in corona proteins. D-E: Quantification of the Western blot results for GP130 (D) and phosphorylated AKT (E). F-H: The mRNA expression level of IL-6 (F) and IL-1β (G), TNF-α (H). (**P* < 0.05, ***P* < 0.01, ****P* < 0.001, *****P* < 0.0001; data are expressed as mean ± standard deviation, n = 3 independent experiments, One–way ANOVA).


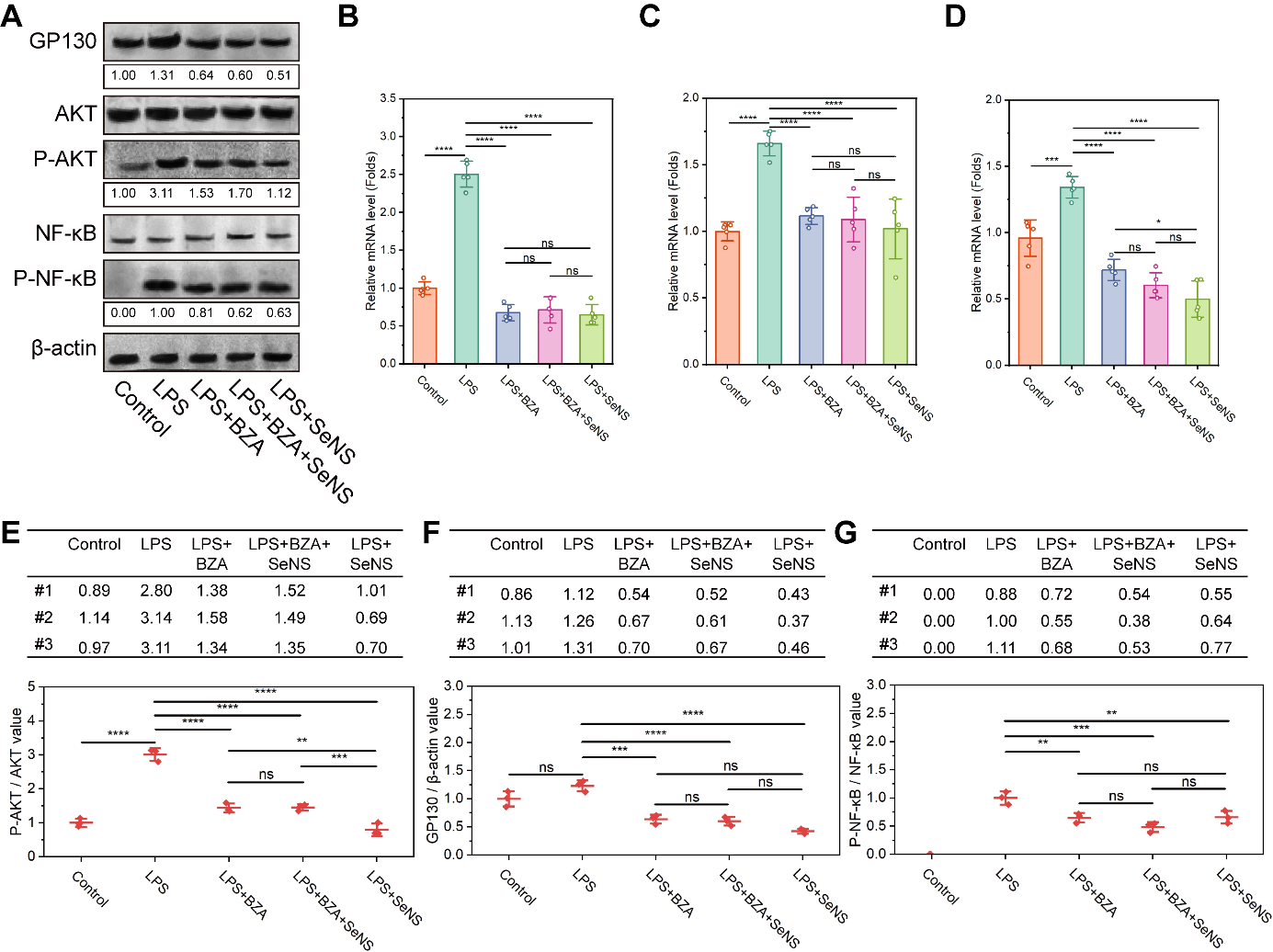


**Figure S38.** Anti-inflammatory mechanism of interaction between GP130 and SeNS. A: Western blot analyses of GP130, AKT, and NF-κB under different treatments. B-D: The mRNA expression level of TNF-α (B), IL-6 (C), and IL-1β (D). E-F: Quantification of the Western blot results for phosphorylated AKT (E), GP130 (F), and phosphorylated NF-κB (G). (**P* < 0.05, ***P* < 0.01, ****P* < 0.001, *****P* < 0.0001; data are expressed as mean ± standard deviation, n = 3 independent experiments, One–way ANOVA).


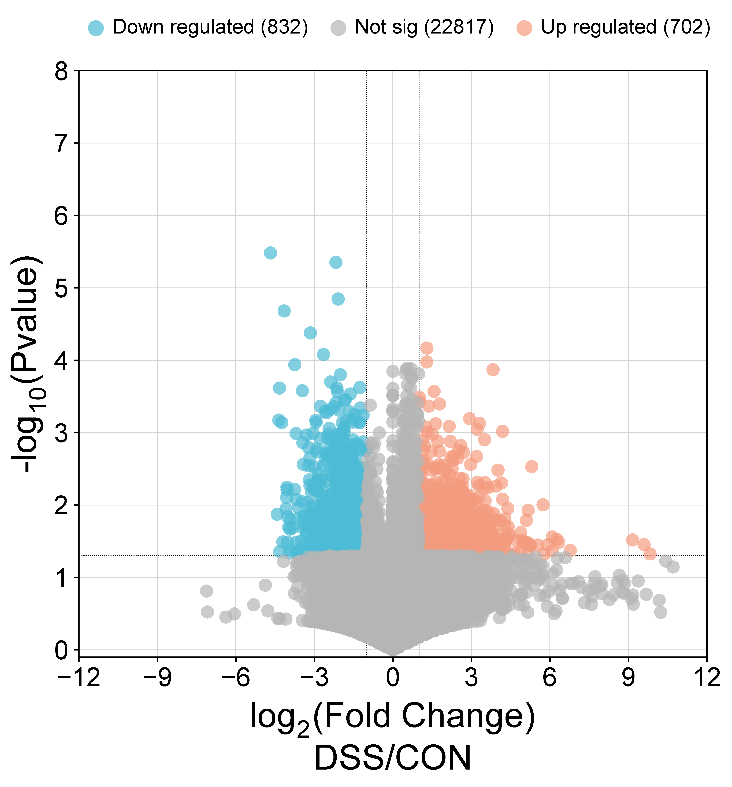
**Figure S39.** Volcano plot comparing gene expression between DSS-induced colitis and control groups. (n = 5 independent experiments).

**
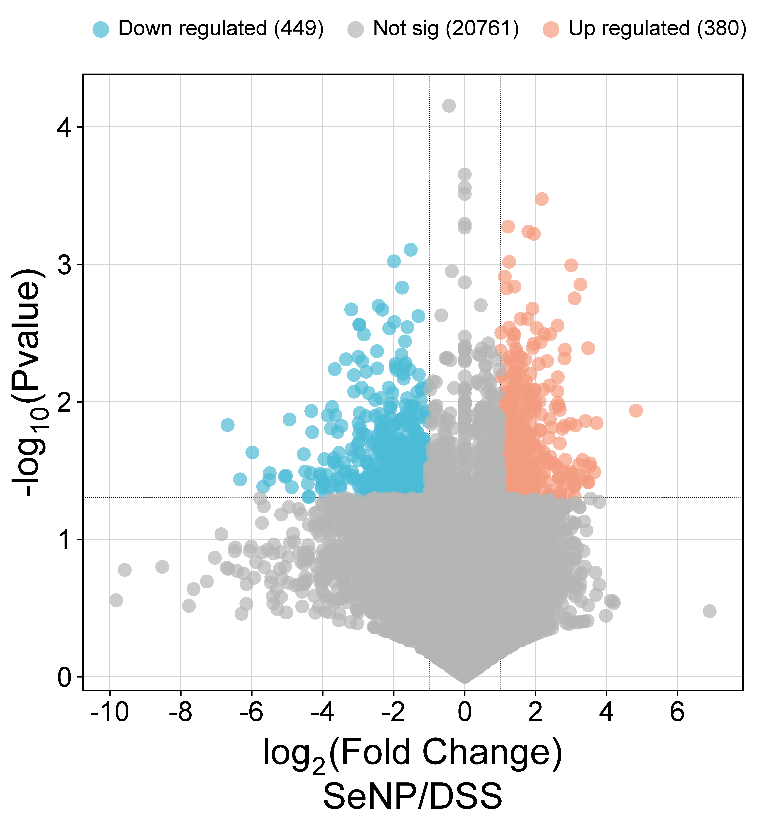
Figure S40.** Volcano plot comparing gene expression between DSS-induced colitis and SeNP-treated groups. (n = 5 independent experiments).

**
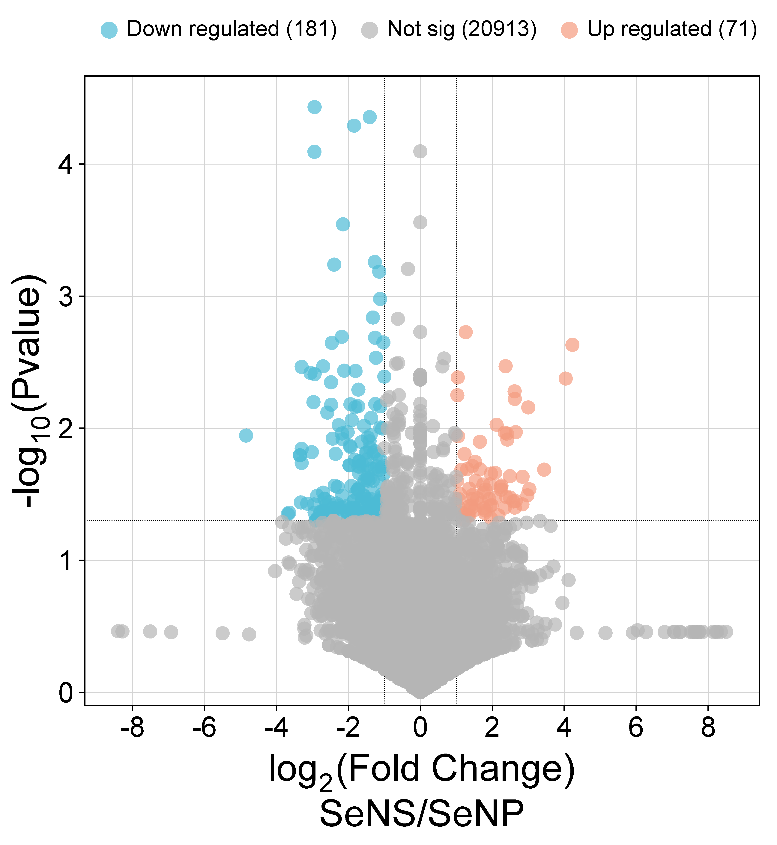
Figure S41.** Volcano plot comparing gene expression between SeNS- and SeNP-treated groups. (n = 5 independent experiments).


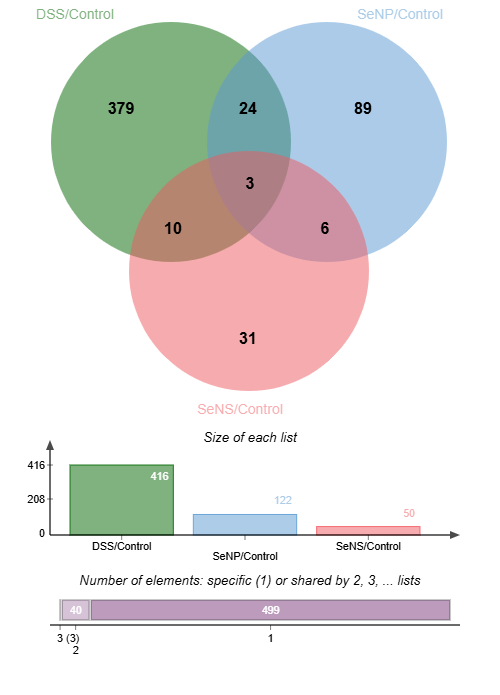
**Figure S42.** Venn diagram showing abnormally expressed gene combinations across DSS, SeNS, and SeNP groups compared to the control group. (n = 5 independent experiments).


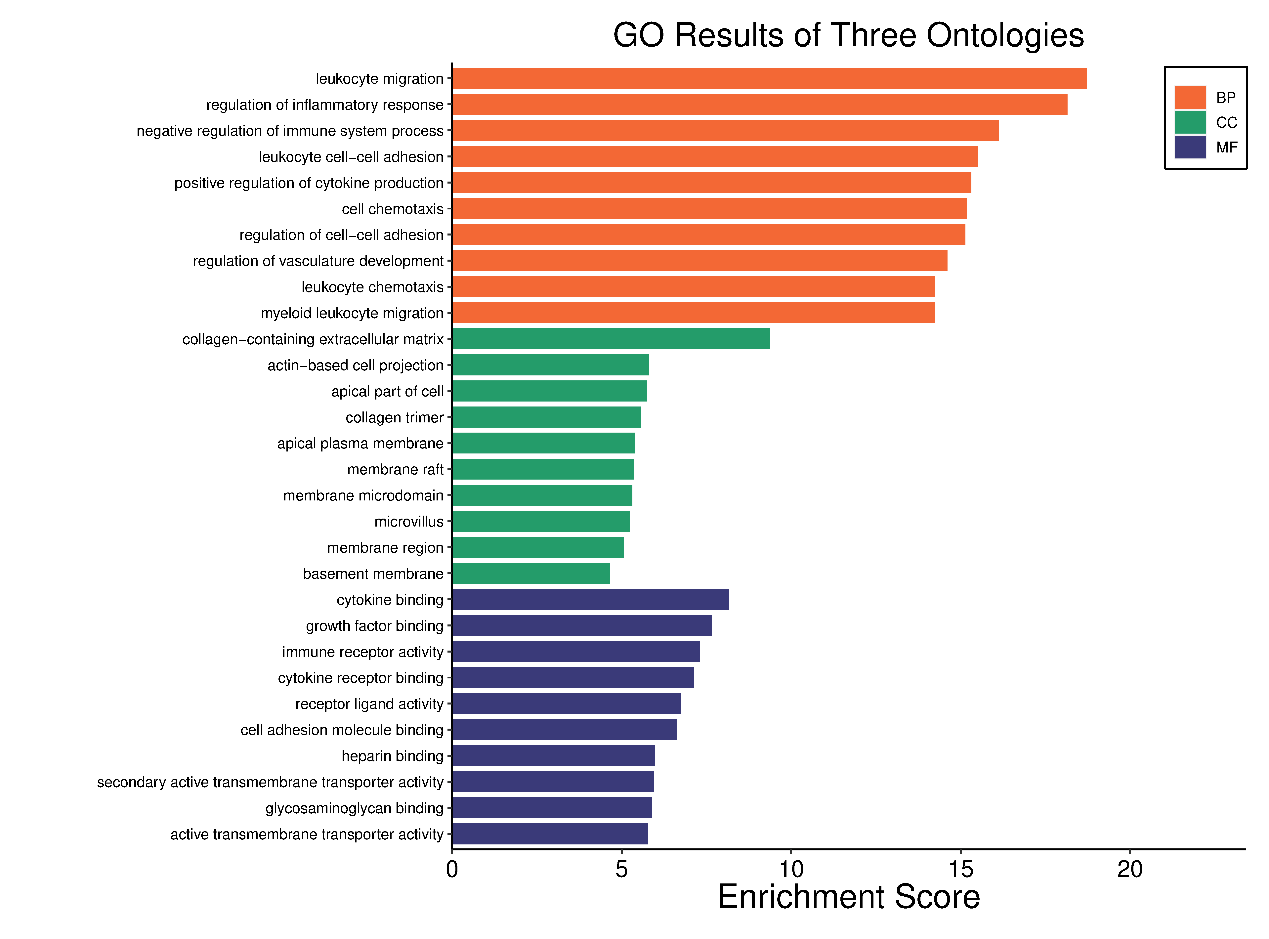
**Figure S43.** GO pathway enrichment analysis of differentially expressed mRNAs in the DSS group compared to the SeNP group. (n = 5 independent experiments).


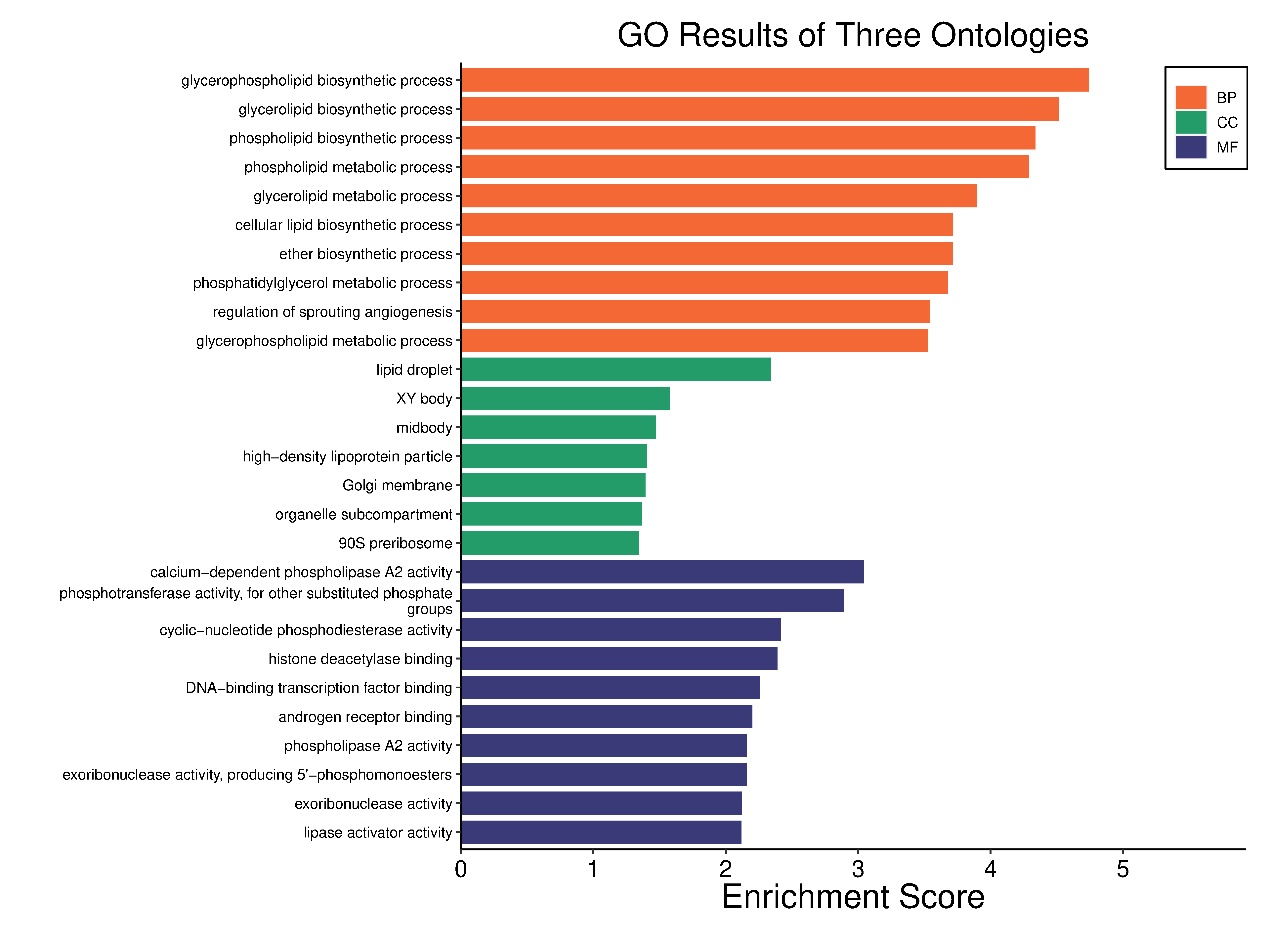
**Figure S44.** GO pathway enrichment analysis of differentially expressed mRNAs in the SeNS group compared to the SeNP group. (n = 5 independent experiments).


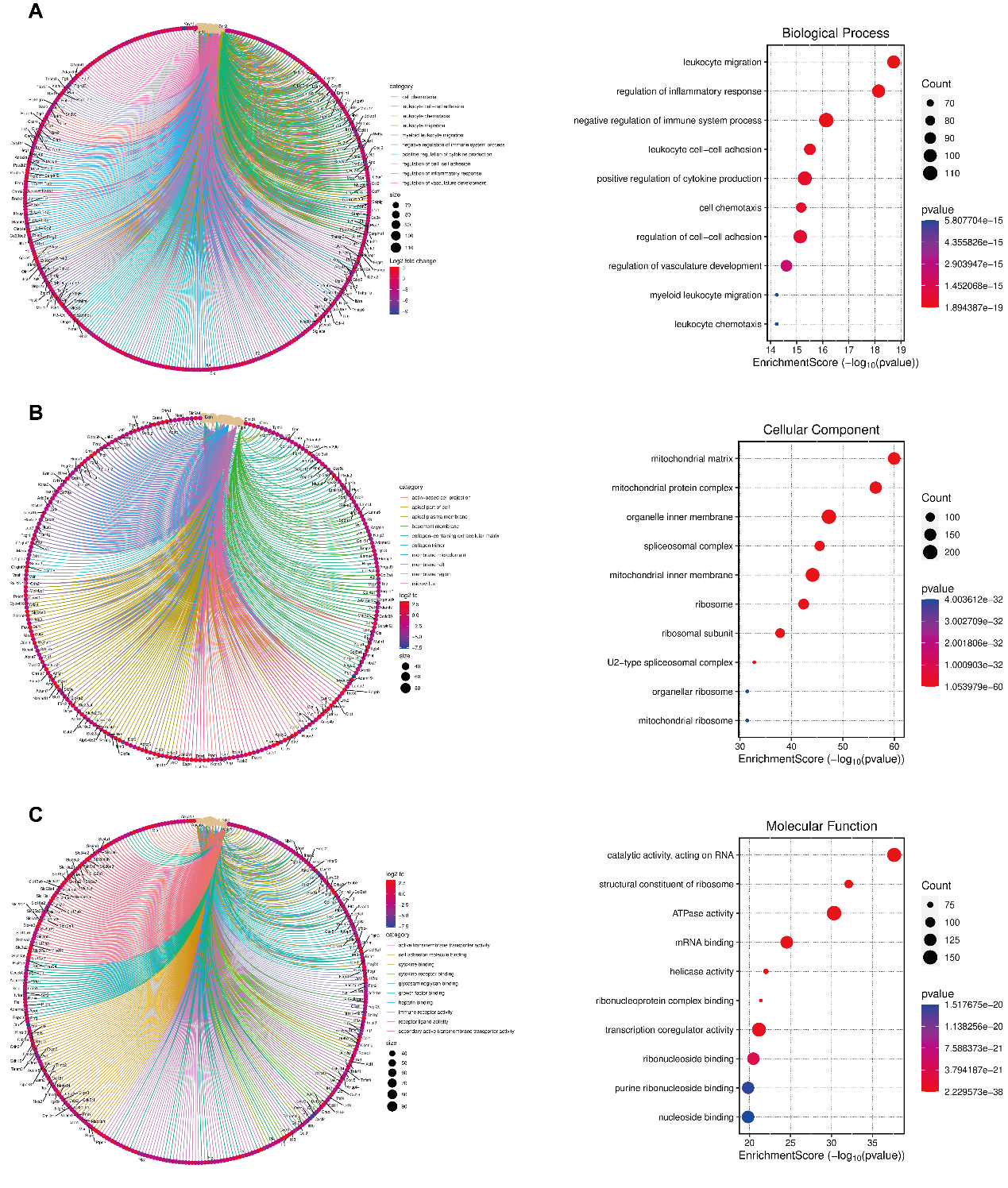
**Figure S45.** GO pathway enrichment results for differentially expressed mRNAs in the DSS group compared to the SeNP group. A: Biological process; B: Cellular component; and C: Molecular function. (n = 5 independent experiments).

**
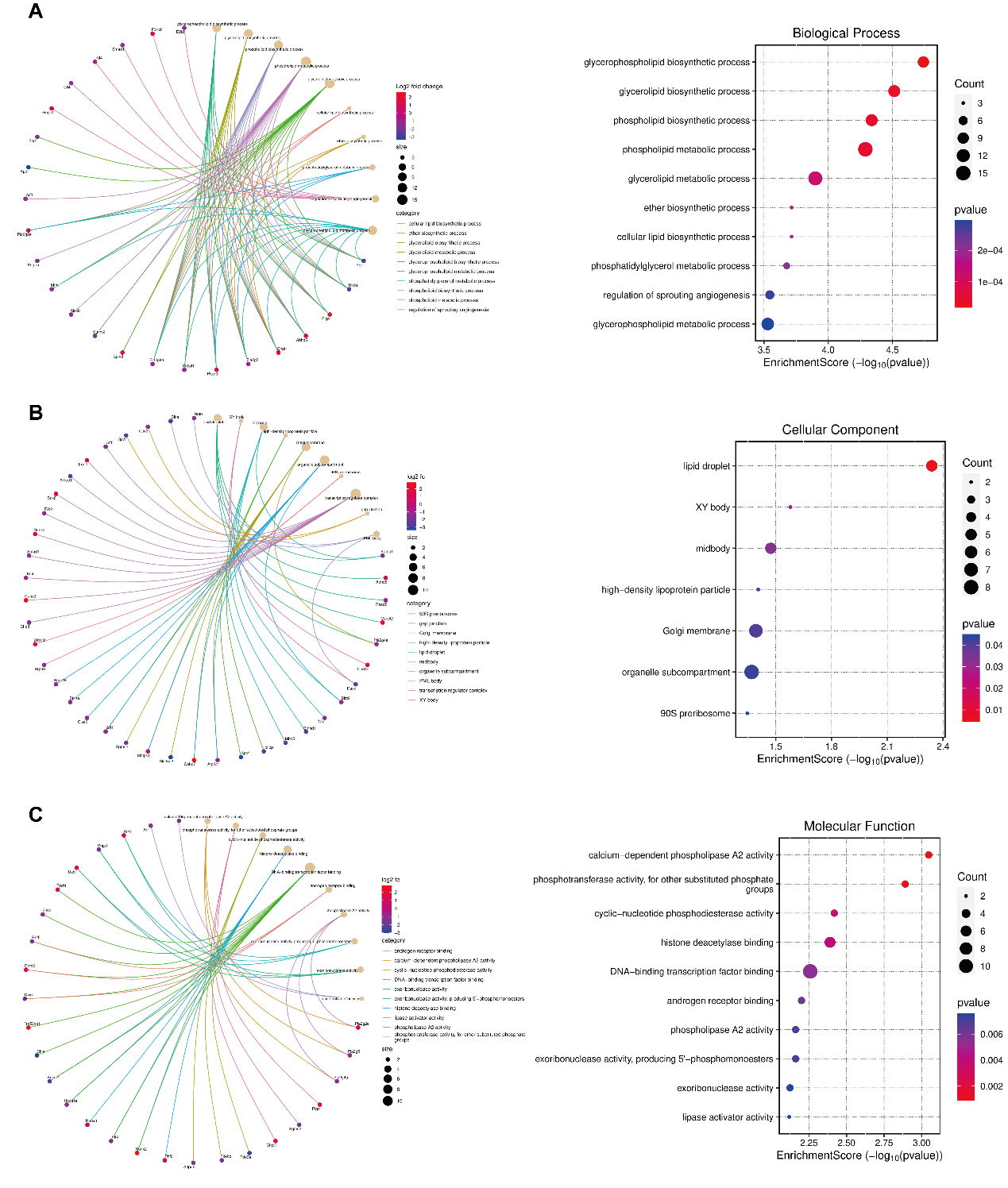
Figure S46.** GO pathway enrichment results for differentially expressed mRNAs in the SeNS group compared to the SeNP group. A: Biological process; B: Cellular component; and C: Molecular function. (n = 5 independent experiments).


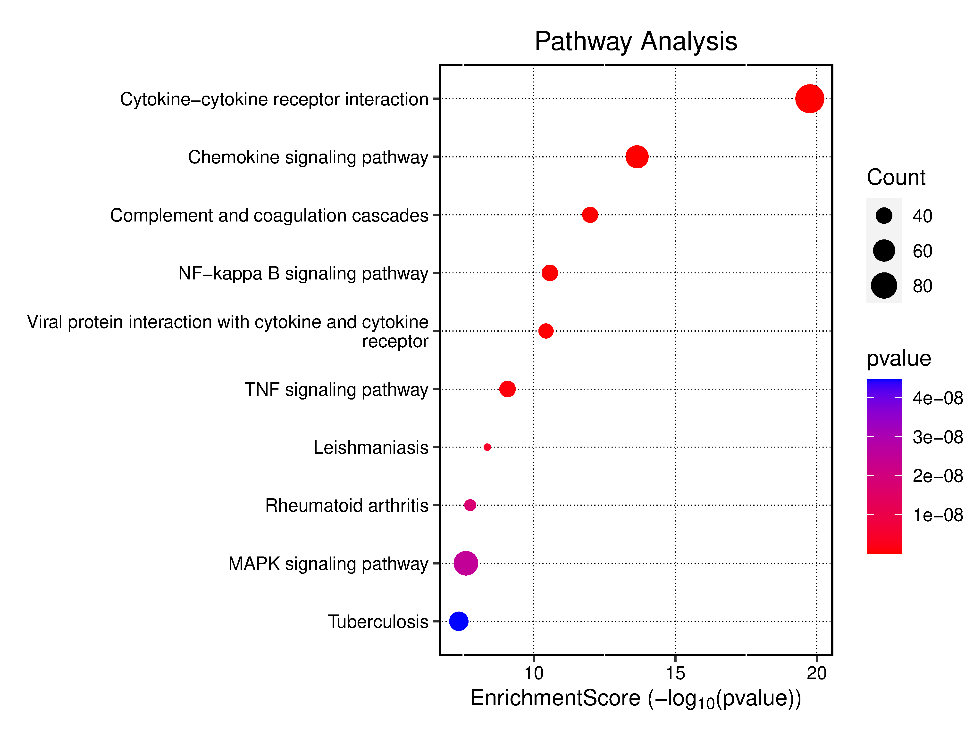
**Figure S47.** KEGG pathway enrichment analysis of differentially expressed mRNAs in the DSS group compared to the control group. (n = 5 independent experiments).


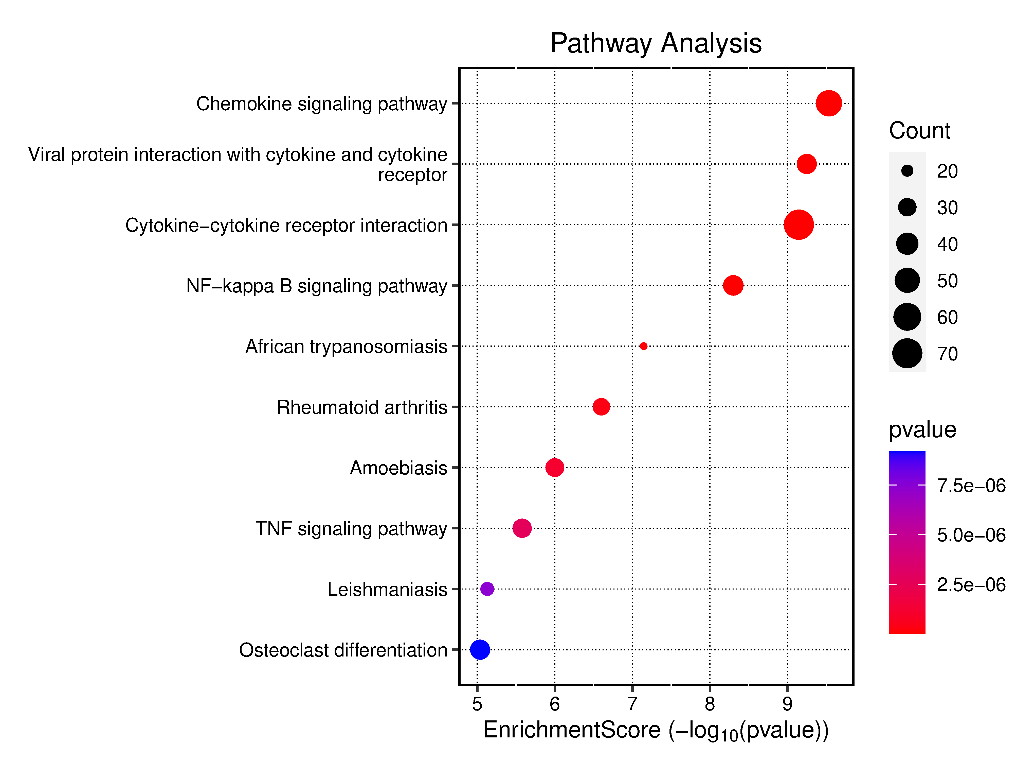
**Figure S48.** KEGG pathway enrichment analysis of differentially expressed mRNAs in the DSS group compared to the SeNP group. (n = 5 independent experiments).


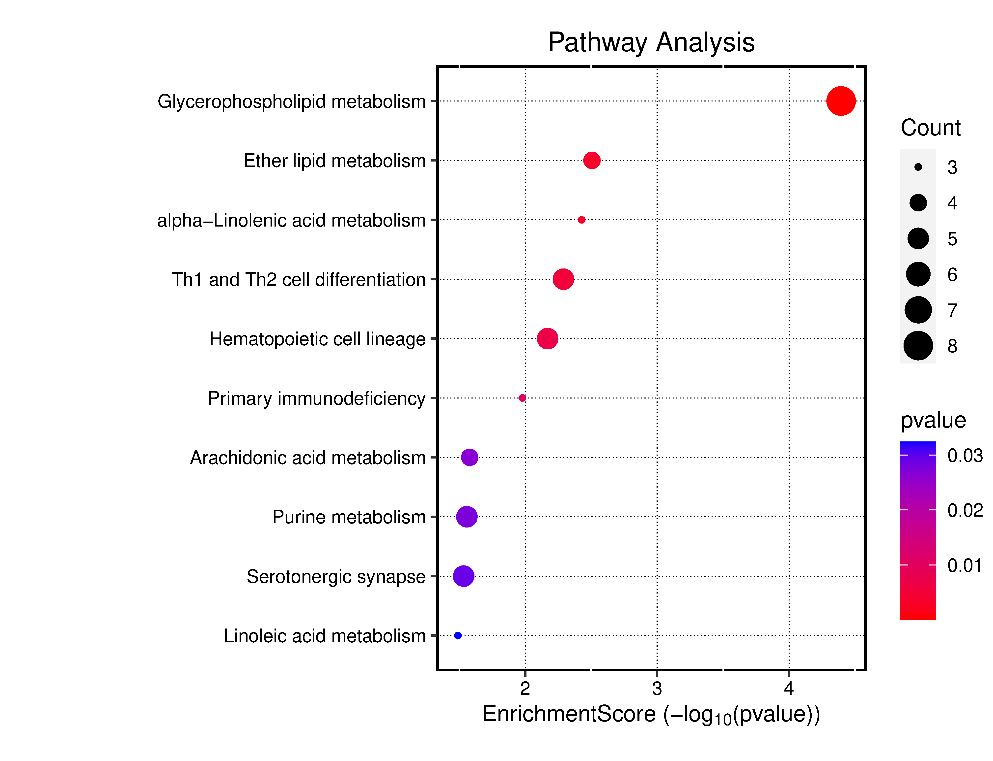
**Figure S49.** KEGG pathway enrichment analysis of differentially expressed mRNAs in the SeNS group compared to the SeNP group. (n = 5 independent experiments).


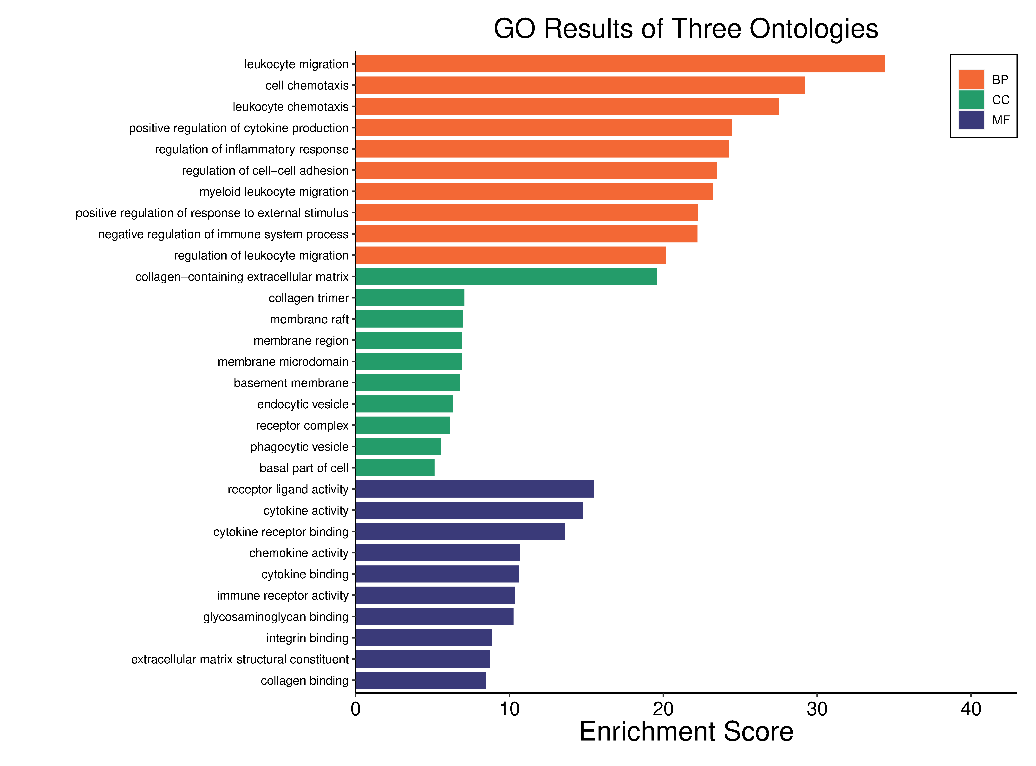
**Figure S50.** GO pathway enrichment analysis of differentially expressed mRNAs in the DSS group compared to the control group. (n = 5 independent experiments).

**
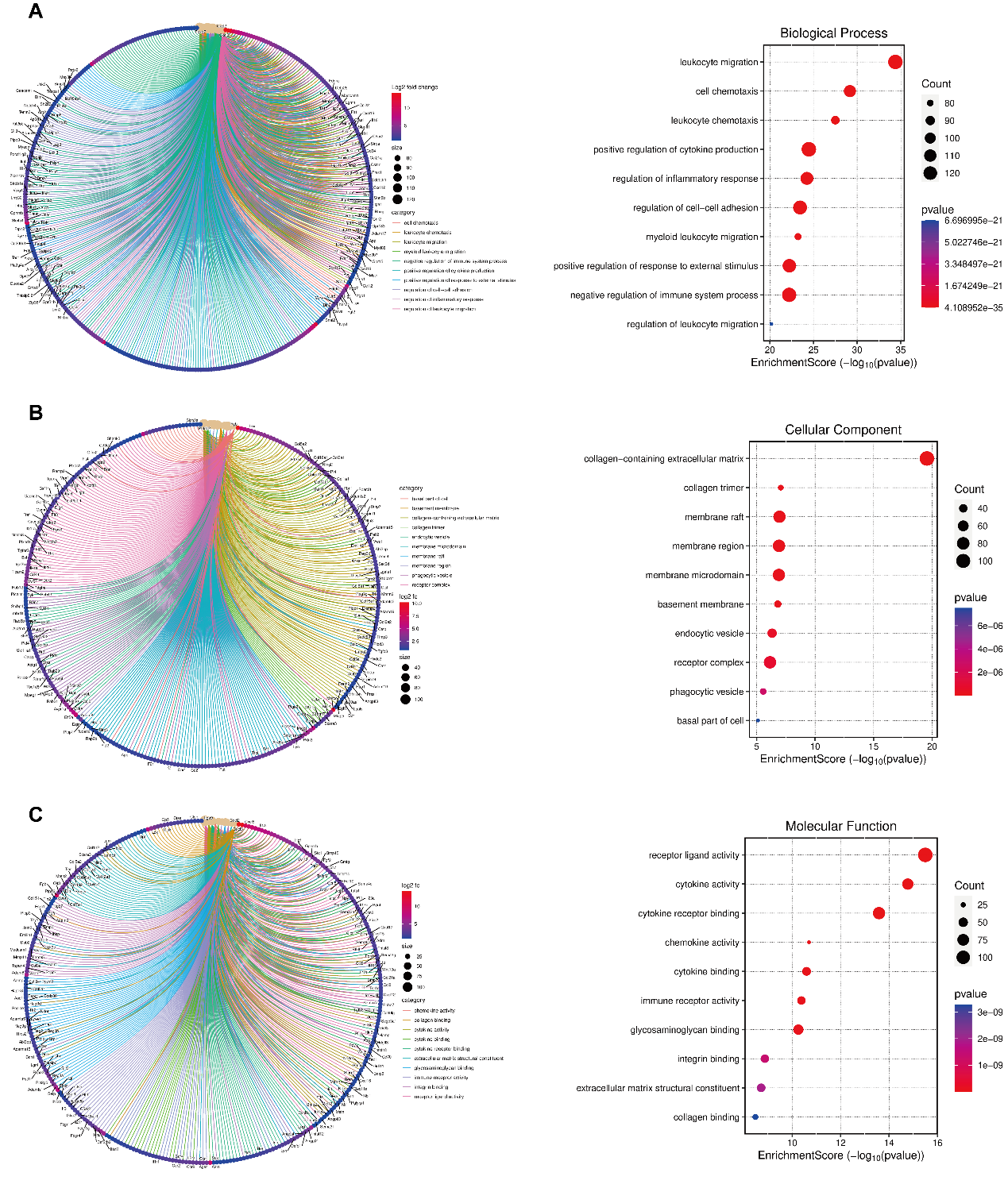
Figure S51.** GO pathway enrichment results for differentially expressed mRNAs in the DSS group compared to the control group. A: Biological process; B: Cellular component; and C: Molecular function. (n = 5 independent experiments).


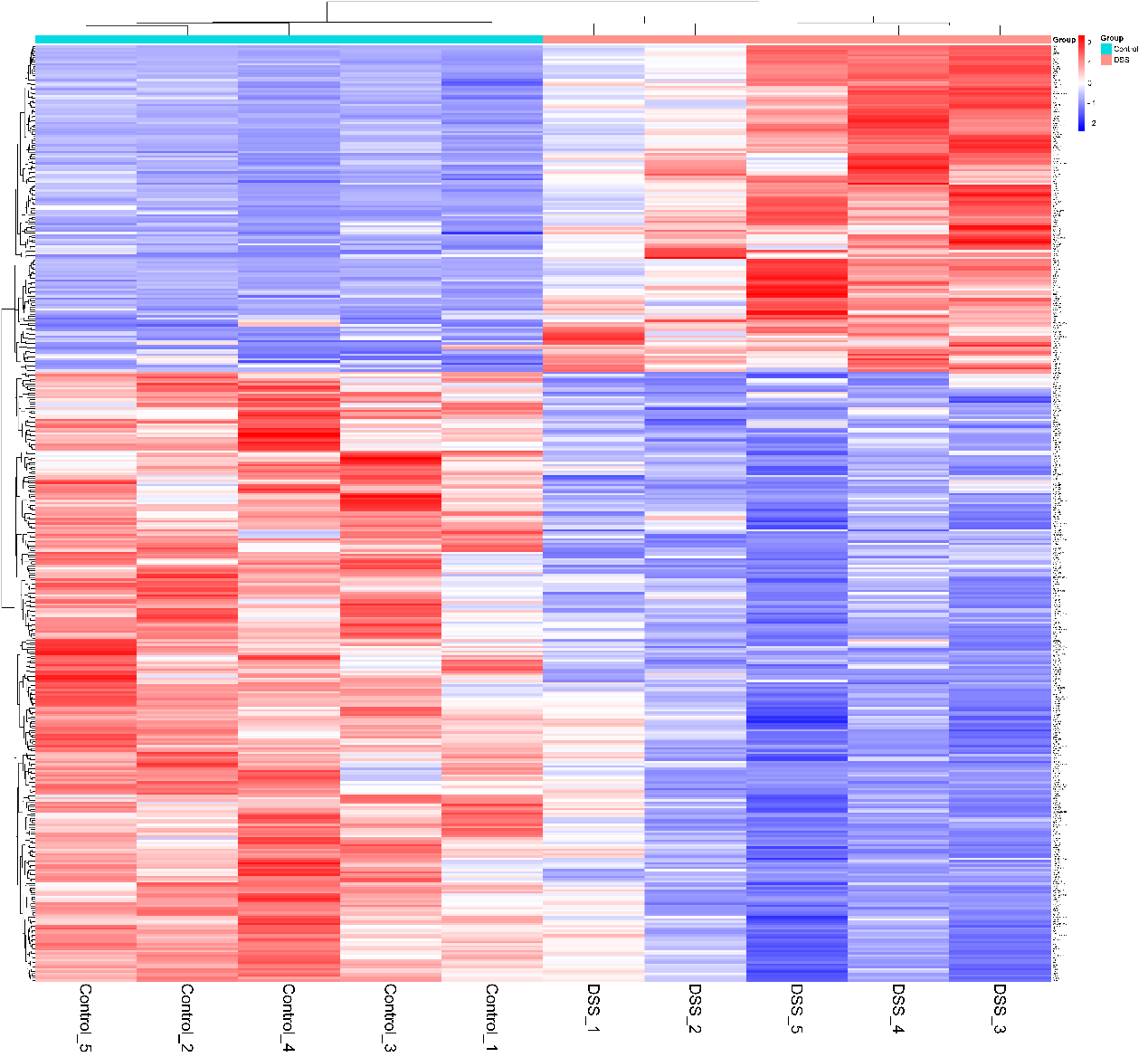
**Figure S52.** Heatmap showing differential gene expression between DSS-induced colitis and control groups. (n = 5 independent experiments).


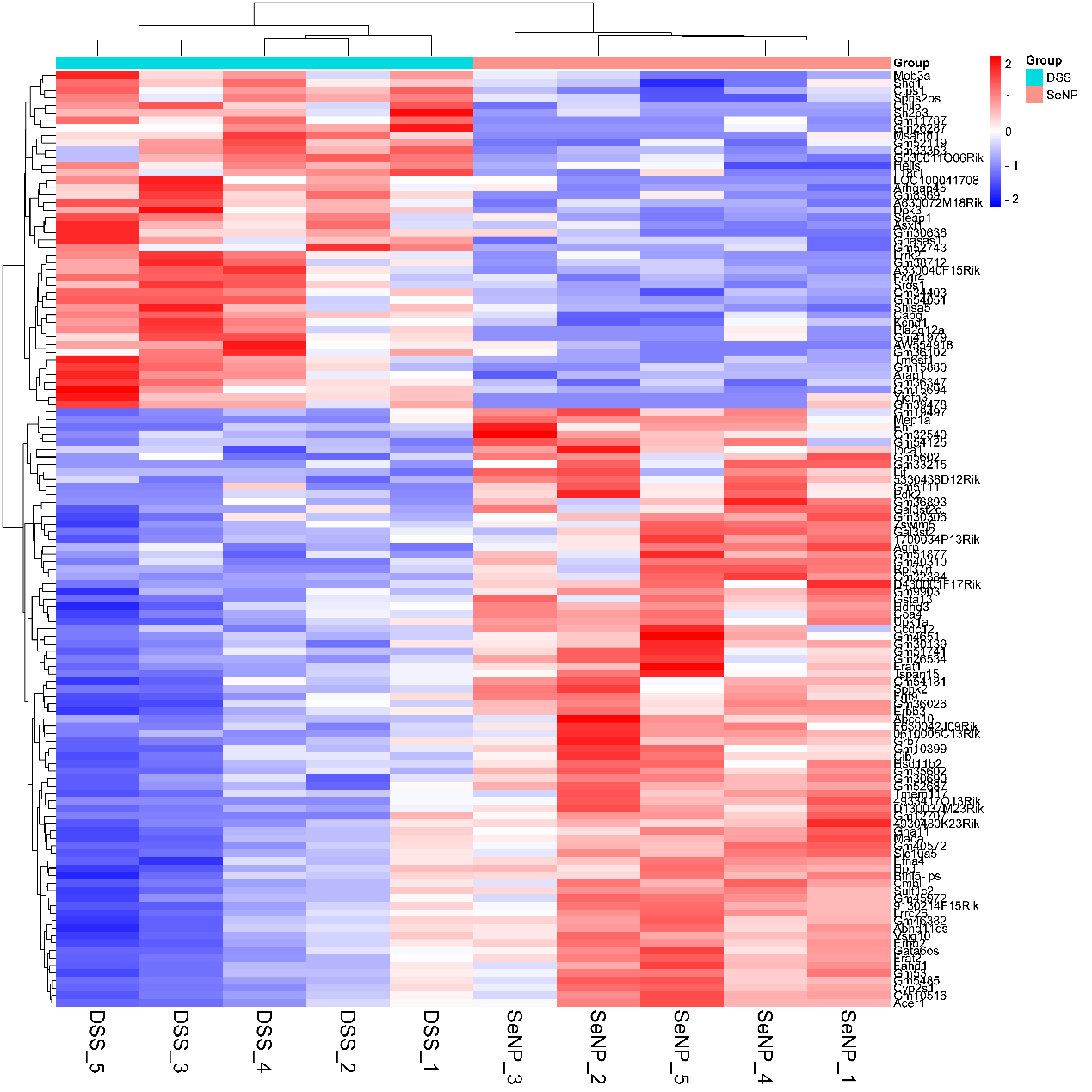
**Figure S53.** Heatmap showing differential gene expression between DSS-induced colitis and SeNP-treated groups. (n = 5 independent experiments).


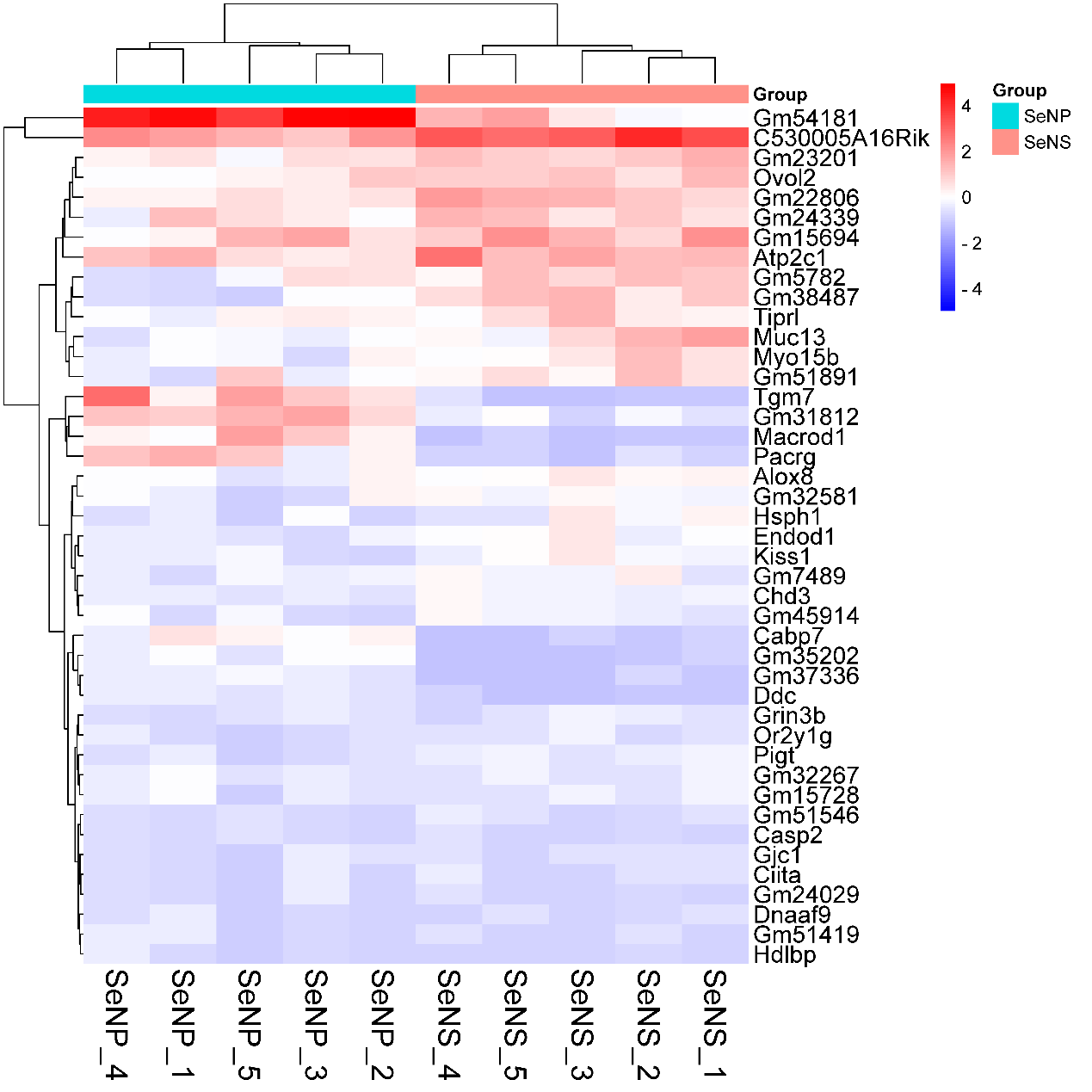
**Figure S54.** Heatmap showing differential gene expression between SeNS- and SeNP-treated groups. (n = 5 independent experiments).

**
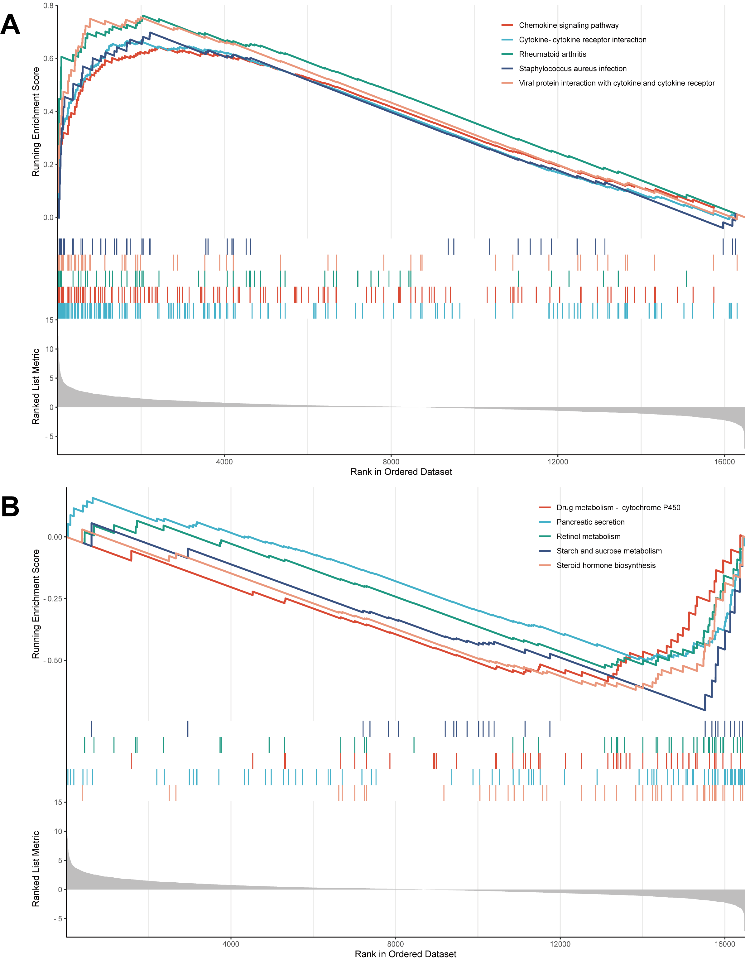
**

| GeneSet | NES | FDR-p |
| --- | --- | --- |
| Cytokine-cytokine receptor interaction | 2.16 | <0.001 |
| Chemokine signaling pathway | 2.05 | <0.001 |
| Rheumatoid arthritis | 2.17 | <0.001 |
| Viral protein interaction with cytokine and cytokine receptor | 2.15 | <0.001 |
| Staphylococcus aureus infection | 2.08 | <0.001 |
| Steroid hormone biosynthesis | -2.23 | <0.001 |
| Pancreatic secretion | -2.02 | <0.001 |
| Drug metabolism - cytochrome P450 | -2.11 | <0.001 |
| Starch and sucrose metabolism | -2.19 | <0.001 |
| Retinol metabolism | -1.91 | <0.001 |

**Figure S55.** GSEA results comparing the DSS and control groups. (n = 5 independent experiments).

**
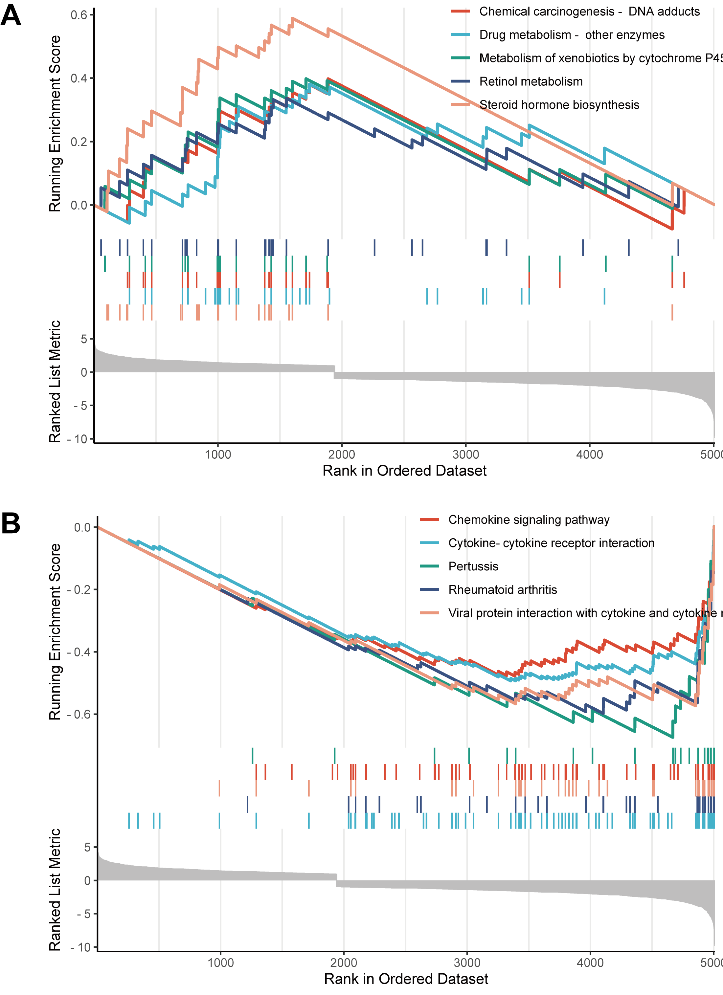
**

| GeneSet | NES | FDR-p |
| --- | --- | --- |
| Steroid hormone biosynthesis | 3.06 | <0.001 |
| Drug metabolism - cytochrome P450 | 2.50 | <0.001 |
| Drug metabolism - other enzymes | 2.16 | <0.001 |
| Chemical carcinogenesis - DNA adducts | 2.13 | <0.001 |
| Metabolism of xenobiotics by cytochrome P450 | 1.99 | <0.001 |
| Cytokine-cytokine receptor interaction | -2.86 | <0.001 |
| Rheumatoid arthritis | -2.73 | <0.001 |
| Viral protein interaction with cytokine and cytokine receptor | -2.72 | <0.001 |
| Chemokine signaling pathway | -2.55 | <0.001 |
| Pertussis | -2.60 | <0.001 |

**Figure S56.** GSEA results comparing the DSS and SeNP groups. (n = 5 independent experiments).


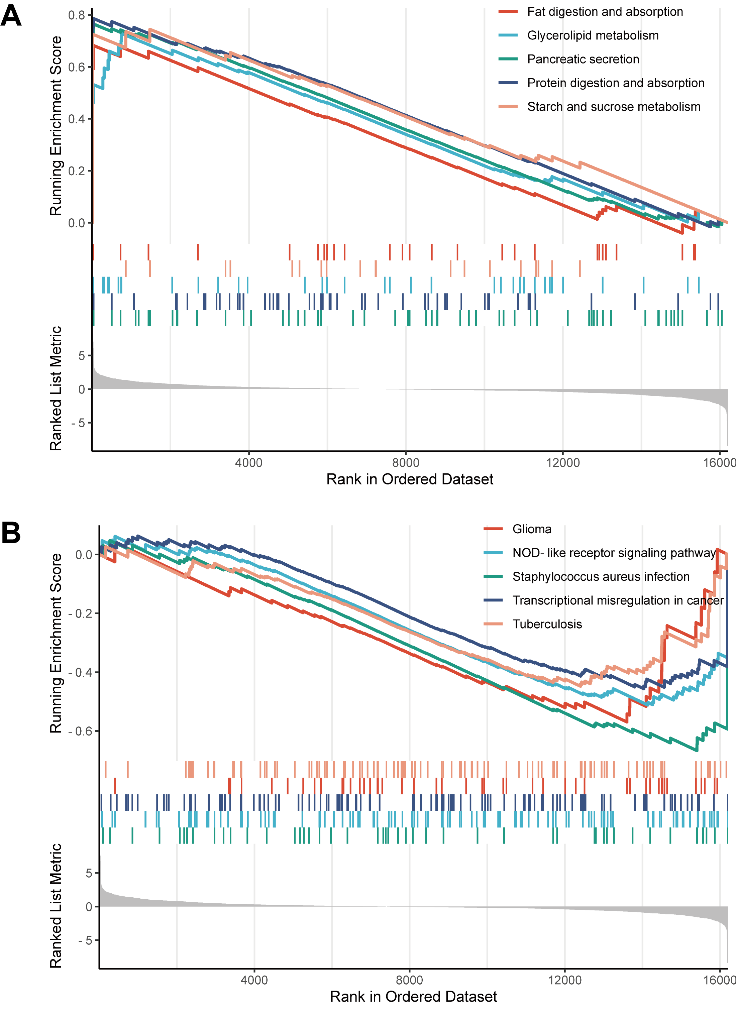


| GeneSet | NES | FDR-p |
| --- | --- | --- |
| Pancreatic secretion | 2.76 | <0.001 |
| Protein digestion and absorption | 2.66 | <0.001 |
| Glycerolipid metabolism | 2.25 | <0.001 |
| Starch and sucrose metabolism | 2.00 | <0.001 |
| Fat digestion and absorption | 1.94 | <0.001 |
| Staphylococcus aureus infection | -2.11 | <0.001 |
| NOD-like receptor signaling pathway | -1.83 | <0.001 |
| Transcriptional misregulation in cancer | -1.64 | <0.001 |
| Glioma | -1.76 | <0.001 |
| Tuberculosis | -1.59 | <0.001 |

**Figure S57.** GSEA results comparing the SeNS and SeNP groups. (n = 5 independent experiments).

**Table S1.** Optimization of the synthesis conditions for SeNS and SeNP.

|  | Reaction conditions | Particles size |
| --- | --- | --- |
| SeNS | 0.5-hour ultrasound, 0 °C, pH = 5.5 | >1000 nm |
|  | 1-hour ultrasound, 0 °C, pH = 5.5 | >1000 nm |
|  | 2-hour ultrasound, 0 °C, pH = 5.5 | >1000 nm |
|  | 4-hour ultrasound, 0 °C, pH = 5.5 | 824.24 ± 134.13 nm |
|  | 6-hour ultrasound, 0 °C, pH = 5.5 | 415.14 ± 174.44 nm |
|  | 0.5-hour ultrasound, 0 °C, pH = 6.5 | >1000 nm |
|  | 1-hour ultrasound, 0 °C, pH = 6.5 | >1000 nm |
|  | 2-hour ultrasound, 0 °C, pH = 6.5 | 564.47 ± 168.69 nm |
|  | 4-hour ultrasound, 0 °C, pH = 6.5 | 362.99 ± 203.13 nm |
|  | 6-hour ultrasound, 0 °C, pH = 6.5 | 163 ± 154.56 nm |
|  | 0.5-hour ultrasound, 0 °C, pH = 7.4 | >1000 nm |
|  | 1-hour ultrasound, 0 °C, pH = 7.4 | 801.25 ± 200.36 nm |
|  | 2-hour ultrasound, 0 °C, pH = 7.4 | 345.15 ± 122.78 nm |
|  | 4-hour ultrasound, 0 °C, pH = 7.4 | 134.45 ± 41.48 nm |
|  | 6-hour ultrasound, 0 °C, pH = 7.4 | 63.58 ± 15.14 nm |
| SeNP | 0.5-hour stirring, 0 °C, pH = 5.5 | >1000 nm |
|  | 1-hour stirring, 0 °C, pH = 5.5 | >1000 nm |
|  | 2-hour stirring, 0 °C, pH = 5.5 | >1000 nm |
|  | 4-hour stirring, 0 °C, pH = 5.5 | 500 ±146.99 nm |
|  | 6-hour stirring, 0 °C, pH = 5.5 | 366.48 ±78.38 |
|  | 0.5-hour stirring, 0 °C, pH = 6.5 | >1000 nm |
|  | 1-hour stirring, 0 °C, pH = 6.5 | >1000 nm |
|  | 2-hour stirring, 0 °C, pH = 6.5 | 852.85 ± 154.47 nm |
|  | 4-hour stirring, 0 °C, pH = 6.5 | 340.54 ± 181.14 nm |
|  | 6-hour stirring, 0 °C, pH = 6.5 | 166.78 ± 78.49 nm |
|  | 0.5-hour stirring, 0 °C, pH = 7.4 | >1000 nm |
|  | 1-hour stirring, 0 °C, pH = 7.4 | >1000 nm |
|  | 2-hour stirring, 0 °C, pH = 7.4 | 488.54 ± 144.54 nm |
|  | 4-hour stirring, 0 °C, pH = 7.4 | 135.48 ± 74.99 nm |
|  | 6-hour stirring, 0 °C, pH = 7.4 | 65.54 ± 19.31 nm |

**Table S2**. Isothermal titration calorimetric data for the binding of SeNS or SeNP to GP130.

|  | N  (Sites) | K_a_  (M^-1^) | ΔH  (kcal•mol^-1^) | -TΔS  (kcal•mol^-1^) | ΔG  (kcal•mol^-1^) |
| --- | --- | --- | --- | --- | --- |
| SeNS | 0.15 | 2.13 × 10^-3^ | -4.14 | -2.15 | -6.29 |
| SeNP | 0.01 | 1.35 × 10^-3^ | -1.98 | -0.16 | -2.15 |

**Table S3.** Comparison of binding energy and Gibbs free energy (ΔG) for SeNS-GP130 and BZA-GP130 complexes.

| System | Binding energy  (kcal•mol^-1^) | ΔG  (kcal•mol^-1^) |
| --- | --- | --- |
| SeNS-GP130 | -5.97 | -7.87 |
| BZA-GP130 | -4.47 | -5.51 |

**Table S4.** Primer sequences used for RT-PCR

| Gene | Sequences |
| --- | --- |
| *Tnf-α* ^[1]^ | Forward: GCTCCCTCTCATCAGTTCCA |
|  | Reverse: GCTTGGTGGTTTGCTACGAC |
| *Il-1β* ^[1]^ | Forward: TCTGAAGCAGCTATGGCAAC |
|  | Reverse: TCAGCCTCAAAGAACAGGTCA |
| *Il-6* ^[1]^ | Forward: AACGAAAGTCAACTCCATCTG |
|  | Reverse: GGTATCCTCTGTGAAGTCTCC |
| *Beta-actin* ^[2]^ | Forward: AGATGTGGATCAGCAAGCA |
|  | Reverse: GCGCAAGTTAGGTTTTGTCA |
| *Pik3cd* | Forward: GATGAGGTGAGGAACGAAG |
|  | Reverse: GCAGAGGACTTGTTGCC |
| *Akt1* | Forward: TTCTCAGTGGCACAATGTCAG |
|  | Reverse: TCCATCTCCTCAGCACCTG |
| *Il6st* ^[3]^ | Forward: CTCTGTTGACAAGCAATGAGACG |
|  | Reverse: TCTTCAGTATGTCTAGCCCCTG |
| *Il6ra* ^[4]^ | Forward: CCTCTGCATTCATTGTTC |
|  | Reverse: GAGATGAGAGGAACAAGCAC |
| *Nfkbia* ^[2]^ | Forward: TGCACTTGGCCATCATCCAT |
|  | Reverse: TCTCGGAGCTCAGGATCACA |
| *Rela* ^[2]^ | Forward: ATGTGGAGATCATTGAGCAGC |
|  | Reverse: CCTGGTCCTGTGTAGCCATT |

[1] Wang, S., Liu, Y., Sun, Q., Zeng, B., Liu, C., Gong, L., Wu, H., Chen, L., Jin, M., Guo, J., Gao, Z., Huang, W. Triple cross-linked dynamic responsive hydrogel loaded with selenium nanoparticles for modulating the inflammatory microenvironment via PI3K/Akt/NF-κband MAPK signaling pathways. *Advanced science* **2023**, 10 (31), e2303167.

[2] Liang, W. J., Yang, H. W., Liu, H. N., Qian, W., Chen, X. HMGB1 upregulates NF-κB by inhibiting IκB-α and associates with diabetic retinopathy. *Life sciences* **2020**, 241, 117146.

[3] Tian, Y., Xu, J., Li, Y., Zhao, R., Du, S., Lv, C., Wu, W., Liu, R., Sheng, X., Song, Y., Bi, X., Li, G., Li, M., Wu, X., Lou, P., You, H., Cui, W., Sun, J., Shuai, J., Ren, F., Zhang, B., Guo, M., Hou, X., Wu, K., Xue, L., Zhang, H., Plikus, M. V., Cong, Y., Lengner, C. J., Liu. Z., Yu. Z. MicroRNA-31 Reduces inflammatory signaling and promotes regeneration in colon epithelium, and delivery of mimics in microspheres reduces colitis in mice. *Gastroenterology* **2019**, 156 (8), 2281-2296.

[4] Xu, L., Yao, Y., Lu, T., Jiang, L. miR-451a targeting IL-6R activates JAK2/STAT3 pathway, thus regulates proliferation and apoptosis of multiple myeloma cells. *Journal of Musculoskeletal & Neuronal Interactions* **2022**, 22 (2), 251-260.
